# An umbrella review of the surgical performance of Harmonic ultrasonic devices and impact on patient outcomes

**DOI:** 10.1186/s12893-023-02057-9

**Published:** 2023-06-29

**Authors:** Robert Kloosterman, George W. J. Wright, Elizabeth M. Salvo-Halloran, Nicole C. Ferko, John Z. Mennone, Jeffrey W. Clymer, Crystal D. Ricketts, Giovanni A. Tommaselli

**Affiliations:** 1 EVERSANA, 113-3228 South Service Rd., Burlington, ON L7N 3H8 Canada; 2 Ethicon, Inc, Cincinnati, OH USA

**Keywords:** Umbrella review, Ultrasonic scalpel, Harmonic, General Surgery, Breast Surgery, Lower Gastrointestinal Surgery, Hepato-Pancreato-Biliary Surgery, Upper Gastrointestinal Surgery, Endocrine

## Abstract

**Background:**

For thirty years, the Harmonic scalpel has been used for precise dissection, sealing and transection. There are numerous meta-analyses on individual surgical procedures with Harmonic, but no overarching review covering all the areas. This umbrella review seeks to summarize the clinical results from the use of Harmonic across surgical fields and broadly quantify its effects on patient outcomes.

**Methods:**

MEDLINE, EMBASE, and Cochrane Databases were searched for meta-analyses (MAs) of randomized controlled trials (RCTs) comparing Harmonic devices to conventional techniques or advanced bipolar (ABP) devices. For each procedure type, the most comprehensive MAs were evaluated. RCTs not already analysed in a MA were also included. Operating time, length of stay, intraoperative blood loss, drainage volume, pain, and overall complications were evaluated, and the methodological quality and certainty of evidence were assessed.

**Results:**

Twenty-four systematic literature reviews were identified on colectomy, hemorrhoidectomy, gastrectomy, mastectomy, flap harvesting, cholecystectomy, thyroidectomy, tonsillectomy, and neck dissection. There were also 83 RCTs included. In every MA evaluated, Harmonic devices were associated with either statistically significant or numerical improvements in every outcome compared with conventional techniques; most MAs reported a reduction in operating time of ≥ 25 min. Harmonic versus ABP device MAs in colectomy and thyroidectomy showed no significant differences in outcomes.

**Conclusion:**

Across surgical procedures, Harmonic devices demonstrated improved patient outcomes for operating time, length of stay, intraoperative bleeding, drainage volume, pain, and overall complications compared to conventional techniques. Additional studies are required to assess differences between Harmonic and ABP devices.

**Supplementary Information:**

The online version contains supplementary material available at 10.1186/s12893-023-02057-9.

## Introduction

As early as the 1970’s, ultrasonic cauterization methods have been used for hemostasis through application of high vibrational frequency rather than electrical energy [1]. Over the last several decades, ultrasonic device-technology has advanced considerably and their use has increased steadily across a wide range of surgical specialties including, but not limited to, colorectal, gynecological, general, thoracic, breast, and bariatric surgery [2]. As a frequently relied upon tool of the surgeon’s armamentarium, surgeons and patients alike have benefitted greatly from ultrasonic devices use for their versatile cutting and coagulating effects [3].

Over the past thirty years Harmonic® devices (Ethicon Inc., Cincinnati, OH) have led the evolution of ultrasonic technology development [4]. Harmonic devices are capable of simultaneous cutting and coagulation using high frequency vibrations in the range of 55 000 Hz [4]. Numerous studies have shown that Harmonic devices, compared to conventional electrosurgery, are associated with superior coagulation with less thermal damage, reduced production of surgical smoke, and improved surgical outcomes [3]. Advanced bipolar (ABP) devices are also available for use in a wide variety of surgical procedures and employ enhanced compression to aid tissue sealing, algorithmic energy control to adjust the current applied to the target tissue, and an integrated cutting blade to seal and dissect soft tissue [5]. Harmonic devices in some studies have shown improved operating times compared to ABP devices [6–8].

Systematic literature reviews (SLRs) and meta-analyses of randomized controlled trials (RCTs) are generally considered the most rigorous and highest level of evidence, but their scope can sometimes be narrow and only focus on a single patient population or intervention/specialty. Additionally, with the growing volume of these types of studies, and the potential for conflicting results, clinicians and decision-makers may be overwhelmed on how to arrive at conclusions when so many SLRs exist on a topic. In the past decade, studies summarizing multiple SLRs, termed “umbrella reviews”, have been published more commonly and have become increasingly influential [9]. Umbrella reviews have the advantage of summarizing large volumes of evidence in a succinct manner, particularly when dozens of meta-analyses exist on a topic.

A 2018 umbrella review on the use of Harmonic devices compared to conventional techniques in surgical oncology showed favorable results across outcomes [10]. However, SLRs on several additional non-oncological procedure types (e.g., cholecystectomy) were beyond the scope of that review. Also, the previous review did not include SLRs comparing Harmonic to ABP devices. Given the growing breadth of SLRs reporting on Harmonic devices, the aim of this study was to conduct an umbrella review spanning *all* surgical procedures for which an SLR on Harmonic versus conventional or ABP comparators was available.

## Methods

An umbrella review was conducted in accordance with the Preferred Reporting Items for Systematic Reviews and Meta-Analyses (PRISMA) 2009 statement (see Supplementary Table 1 for PRISMA checklist) [11]. The umbrella review was based on a periodically updated systematic literature review that started in 2016. Review methods including the question, search strategy, eligibility criteria, and planned risk of bias assessments were developed a priori. However, observational studies were later excluded given the increasing number of RCTs being published in recent years. Systematic reviews and meta-analyses of RCTs were the focus since they are inherently lower risks of bias than observational studies. For completeness, RCTs of Harmonic devices published after included SLRs or from specialties where no published SLRs were identified were also evaluated.

### Search strategy

The search strategy was developed by a medical information specialist in consultation with the review team. MEDLINE, EMBASE, Cochrane Central Register of Controlled Trials, and Cochrane Database of Systematic Reviews were systematically searched for RCTs, systematic reviews and meta-analyses published from January 1, 2010, to January 31, 2022. Database searches were updated on January 31, 2022 (see Supplementary Appendix A for additional details). All searches were limited to English-language articles on human patients. The complete search strategy is provided in (Supplementary Appendix A). Search results were informed by a broad search strategy that also included non-randomized studies. Only RCTs and systematic reviews with meta-analyses were considered for inclusion in the umbrella review.

### Eligibility criteria and data extraction

Two independent reviewers individually assessed the eligibility of retrieved records using Distiller SR [12]. Inclusion conflicts were resolved through discussion, and when necessary, by a third reviewer. All RCTs and systematic reviews of patient populations where Harmonic devices were used according to manufacturer specifications and recommendations were eligible for inclusion. The following PICOS (population, intervention and comparator, outcome, and study design) criteria were used to assess identified records for inclusion. Studies using Harmonic devices as an intervention compared to any conventional techniques or advanced energy devices (including ultrasonic and ABP surgical devices) were included. Conventional techniques include basic monopolar (Bovie) and bipolar electrosurgery and manual techniques such as clamp, cut, and tie. Only studies reporting at least one of the following outcomes were included in the qualitative synthesis of meta-analyses: operating time (min), length of stay (days), intraoperative blood loss (mL), drainage volume (mL), pain (visual analogue scale score), or overall complications (number/%), as reported. Effect measures reported for all continuous outcomes were mean differences and those for overall complications were either odds ratios or relative risk. Systematic reviews were eligible for inclusion in the umbrella review if they summarized RCTs with a meta-analysis component. Studies that combined outcomes for Harmonic devices with other interventions and did not stratify results were excluded. Systematic reviews that included both non-randomized studies and RCTs and did not stratify results by study design were also excluded.

For completeness, recent RCTs were included that were not included by the identified systematic reviews. Recent RCTs are defined as those published after the latest search date for the most recent meta-analysis of a specialty or procedure type. RCTs from procedure types for which no systematic review was found are referred to as orphan RCTs. Recent RCTs or RCTs not captured in any of the eligible systematic review are referred to as additional RCTs. The RCTs were then assessed for eligibility using the same PICOS criteria as the systematic reviews.

Data from each study were extracted by one reviewer, with a quality check performed by a second reviewer. Discrepancies were resolved through discussion, and when necessary, by a third reviewer. The following were extracted from each study: first author, publication year, surgical procedure(s), comparator type, number of included RCTs and participants, and outcomes.

When multiple systematic reviews on the same surgical procedure were available, the most comprehensive review was selected for inclusion in the qualitative summaries to avoid double counting patients, to improve the rigor of the assessment, and simplify the results summary. The most comprehensive review for each procedure type was determined based on the year of publication, number of included RCTs and patients, and assessment of outcome certainty based on GRADE assessments [1]. In situations where an outcome of interest was not reported in the most comprehensive systematic review but was available in another systematic review, outcome data from that systematic review was included and summarized. The effect estimates and significance level for each outcome across procedure types were plotted to summarize the available evidence. Only meta-analyses of two or more RCTs were included in the qualitative summaries.

### Study quality assessment

The methodological quality of each review was assessed and summarized using AMSTAR-2 which contains 16 items and provides an overall quality scored based on weaknesses in key domains [13]. Grading of Recommendations, Assessment, Development, and Evaluation (GRADE) was used to assess the certainty of results reported by included systematic reviews [1]. Where possible, author-reported GRADE assessments were used. For the RCTs, the National Institute for Health and Care Excellence (NICE) checklist was used to assess study quality [14].

## Results

### Characteristics of included reviews and overall results

**Fig. 1:**
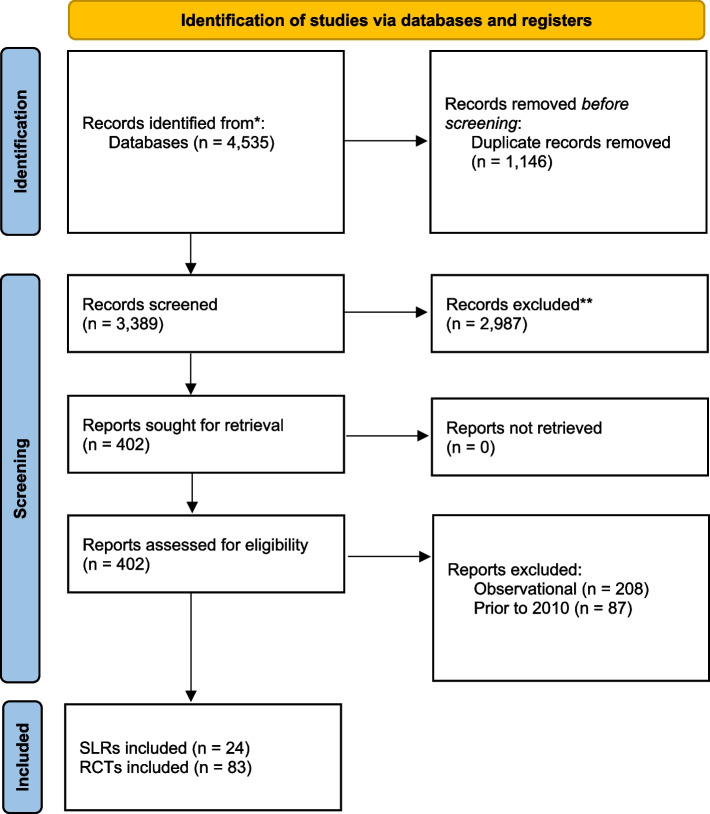
PRISMA flow diagram of study selection. Abbreviations: PRISMA = Preferred Reporting Items for Systematic Reviews and Meta-Analyses; RCT = randomized controlled trial; SLR = systematic literature review

**Fig. 2:**
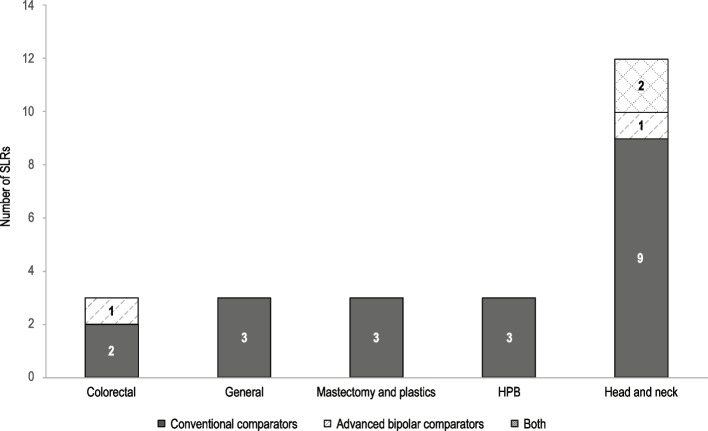
Distribution of Harmonic SLRs across surgical specialties, stratified by comparator device type. Abbreviations: HPB = hepato-pancreatico-biliary; SLR = systematic literature review

Excluding duplicates, 3389 studies were screened for inclusion in the umbrella review. Of those, 24 systematic reviews and 83 RCTs were included. The study screening and selection process is shown in the PRISMA diagram Fig. 1. Eligible systematic reviews that compared Harmonic to conventional methods were identified for nine procedures including cholecystectomy, colectomy, flap harvesting, gastrectomy, hemorrhoidectomy, mastectomy, neck dissection, thyroidectomy, and tonsillectomy (Fig. 2). Table 1 summarizes the details of included systematic reviews. Two meta-analyses were identified that compared Harmonic to ABP devices for colectomy and thyroidectomy (Table 2) [15, 16].

**Table 1:** Characteristics of included systematic reviews

**Review**	**No. of RCTs**	**No. of patients**	**Comparator**	**Surgical Procedure**	**Outcomes Reported**	**GRADE rating**
**Colectomy**						
Tou, 2011 [15]	6	417	Conventional and ABP	Laparoscopic Colectomy	Operating time, length of stay, conversion to alternative technique, postoperative blood loss, overall complications, total cost	Low to Moderate
**Hemorrhoidectomy**						
Mushaya, 2014 [17]	8	468	Conventional	Hemorrhoidectomy	Operating time, length of stay, pain, time to return to work, overall complications	Low to High
Balciscueta, 2021 [18]	4	278	Conventional	Hemorrhoidectomy (grade III-IV)	Pain	Low
**Gastrectomy**						
Chen, 2014 [19]	7^a^	1930	Conventional	Open gastrectomy for gastric cancer	Operating time, length of stay, drainage volume, intraoperative blood loss, transfusion, overall complications, number of lymph nodes dissected	Moderate
Cheng, 2015 [20]	10	935	Conventional	Gastrectomy and D2 Lymphadenectomy for Gastric Cancer	Operating time, length of stay, drainage volume, intraoperative blood loss, postoperative death, hospital costs	Low to Moderate
Sun, 2015 [21]	5	489	Conventional	Open gastrectomy for gastric cancer	Operating time, length of stay, drainage volume, transfusion, hemorrhage, overall complications	Low to Moderate
**Mastectomy**						
Cheng, 2016 [22]	12	965	Conventional	Mastectomy and BCS with LND	Operating time, length of stay, drainage volume, intraoperative blood loss, hematoma, seroma, overall complication, wound infection	Low to Moderate
Zhang, 2018 [23]	10^b^	NR	Conventional	Mastectomy and BCS with LND	Operating time, length of stay, drainage volume, drain time, intraoperative blood loss, hematoma, seroma, overall complication, wound infection, other complications	Low to Moderate
**Flap Harvesting**						
Kim, 2022 [24]	8	505	Conventional	Flap surgery for various flap types	Operating time, drainage volume, Intraoperative blood loss, hematoma, wound infection, additional complication, overall complication	Low to Moderate
**Cholecystectomy**						
Sasi, 2010 [25]	7	695	Conventional	Laparoscopic Cholecystectomy	Operating time, length of stay, conversion to alternative technique, pain, time to return to work, overall complications, bile leak, gallbladder perforation	Low to High
Xiong, 2012 [26]	11	1434	Conventional	Laparoscopic Cholecystectomy	Operating time, length of stay, conversion to an alternative technique, pain, intraoperative bleeding, nausea/vomiting, other complications, bile leak, gallbladder perforation	Low to High
Jiang, 2017 [27]	19	1955	Conventional	Laparoscopic Cholecystectomy	Operating time, length of stay, pain, intraoperative blood loss, gallbladder perforation	Low to Moderate
**Thyroidectomy**						
Cirocchi, 2010 [28]	7	608	Conventional	Total thyroidectomy	Operating time, drainage volume, intraoperative blood loss, vocal cord and RLN palsy/paralysis—temp, vocal cord and RLN palsy/paralysis—perm, hypocalcemia	Moderate
Ecker, 2010 [29]	10	485	Conventional and ABP	Total, hemi-, and subtotal thyroidectomy	Operating time, length of stay, drainage volume, pain, intraoperative blood loss, overall complications	Very Low to Low
Melck, 2010 [30]	9	822	Conventional	Total and subtotal thyroidectomy	Operating time, vocal cord and RLN palsy/paralysis—temp, hypocalcemia	Moderate
Cheng, 2015 [31]	14	2516	Conventional	Total thyroidectomy	Operating time, length of stay, drainage volume, pain, intraoperative blood loss, hematoma, seroma, vocal cord and RLN palsy/paralysis—temp, hypocalcemia	Low to Moderate
Cannizzaro, 2016 [16]	17	2596	Conventional and ABP	Total thyroidectomy	Operating time, length of stay, pain, intraoperative blood loss, overall complications	Very Low to Low
Cheng, 2016 [32]	7	954	Conventional	Total thyroidectomy	Total costs	NA
Revelli, 2016 [33]	21	3125	Conventional	Total thyroidectomy	Operating time, length of stay, drain placed, pain, intraoperative blood loss, additional wound complications, vocal cord and RLN palsy/paralysis—temp, hypocalcemia	Low to Moderate
Upadhyaya, 2016 [34]	7	981	ABP	Partial and total thyroidectomy	Operating time, length of stay, intraoperative blood loss, postoperative blood loss, mean thyroid weight, calcium levels	Low to Moderate
Aires, 2018 [35]	31	NR	Conventional	Total thyroidectomy	Operating time, intraoperative blood loss, hematoma, laryngeal nerve injury, number of lymph nodes removed	Moderate
Hua, 2019 [36]	23	5408	Conventional	Total thyroidectomy	Hematoma	NA
**Tonsillectomy**						
Alexiou, 2011 [37]	11^c^	3139	Conventional	Total tonsillectomy	Operating time, pain, intraoperative blood loss, postoperative blood loss	Very Low to Low
**Neck Dissection**						
Ren, 2015 [38]	7	406	Conventional	Neck dissection	Operating time, length of stay, drainage volume, intraoperative blood loss	Low to Moderate

For specific details of GRADE assessments refer to Supplementary Table 5

*Abbreviations*: *ABP* advanced bipolar, *BCS* breast conserving surgery, *GRADE* Grades of Recommendation, Assessment, Development and Evaluation, *LND* lymph node dissection, *RCT* randomized controlled trial, *RLN* recurrent laryngeal nerve

^a^Chen, 2014 included 19 studies (7 RCTs and 12 non-RCTs). Outcomes were stratified by study design and only data from RCTs were included in this review

^b^Zhang, 2018 included 20 studies (10 RCTs and 10 non-RCTs). Outcomes were stratified by study design and only data from RCTs were included in this review

^c^Alexiou, 2011 included 33 RCTs comparing various methods of tonsillectomy. Of these, 11 compared Harmonic devices to conventional techniques and were included in this review

**Table 2:** Summary systematic reviews comparing Harmonic to advanced bipolar devices

**Study**	**No. of patients**	**Effect estimate** **(95% CI)**	***p*-value**
**Operating Time**			
Colectomy (Tou, 2011) [15]	181	MD: -3.22 (-15.31, 8.87)	0.6
Thyroidectomy (Cannizzaro, 2016) [16]	474	WMD: -9.67 (-20.27, 0.92)	0.074
**Length of Stay**			
Colectomy (Tou, 2011) [15]	181	MD: 0.41 (-0.49, 1.31)	0.38
Thyroidectomy (Cannizzaro, 2016) [16]	284	WMD: -0.01 (NR)	0.778
**Blood Loss**			
Colectomy (Tou, 2011) [15]	181	MD: -3.74 (-19.04, 11.55)	0.63
Thyroidectomy (Cannizzaro, 2016) [16]	322	WMD: -3.61 (-13.6, 6.39)	0.48
**Overall Complications**			
Colectomy (Tou, 2011) [15]	208	RR: 0.81 (0.46, 1.4)	0.45
Thyroidectomy (Cannizzaro, 2016) [16]	474	OR: 1.47 (0.98, 2.12)	0.061

Mean difference is defined as (Harmonic value – comparator value) and relative risk is defined as (Harmonic value / comparator value)

*Abbreviations*: *CI* confidence interval, *MD* mean difference, *OR* odds ratio, *RR* risk ratio, *WMD* weighted mean difference

Of the 83 included RCTs, 60 reported on procedure types for which an eligible systematic review was identified (additional RCTs; Supplementary Table 2) and 23 reported on procedure types for which an eligible SLR was not identified (orphan RCTs; Supplementary Table 3).

AMSTAR-2 assessments of SLR quality found that the 24 included SLRs ranged from critically low to low quality (Supplementary Table 4). In general, more than two thirds of studies received positive assessments for questions 1 (24/24; inclusion of PICO in the inclusion criteria and research question), 6 (16/24; data extraction in duplicate), 9 (19/24; use satisfactory risk of bias tool), 13 (16/24; considered sources of bias in the discussion), and 14 (18/24; provided satisfactory explanation for heterogeneity). Most studies did not have a registered or published protocol (20/24; question 2), no studies provided specific rationale for the inclusion of RCTs (24/24; question 3), almost none reported a list of excluded studies (23/24; question 7), and most did not report the sources of funding for RCTs (22/24; question 10). None of the included systematic reviews performed sensitivity analyses to adjust for source of heterogeneity where present (24/24; question 11).

The GRADE assessments of each meta-analysis for the six included outcomes from the 24 included SLRs, most outcomes were moderate (*n* = 37) to low (*n* = 35) with some high (*n* = 3), and some very low (*n* = 7) certainty ratings (Supplementary Table 5). From the 82 outcomes assessed with GRADE, 61 were downgraded for inconsistency, 45 for imprecision, eight for risk of bias, and seven for publication bias. All of the most comprehensive SLRs apart from two included moderate to low certainty meta-analyses of the six outcomes of focus [16, 37].

The NICE checklist assessments of the orphan and additional RCTs showed that there was balance in dropouts between groups in almost all studies (79/83), all studies lacked evidence suggesting that more outcomes were assessed than were reported (83/83), and most studies used an intention to treat analysis (67/83; Supplementary Table 6). Issues identified with the RCTs were that randomization methods were not clear or inadequate in 51/83 studies, concealment of treatment allocation was not clear or inadequate in 52/83 studies, and blinding of participants, providers, and outcome assessors was unclear or inadequate in all RCTs (83/83).

#### Operating time

**Fig. 3:**
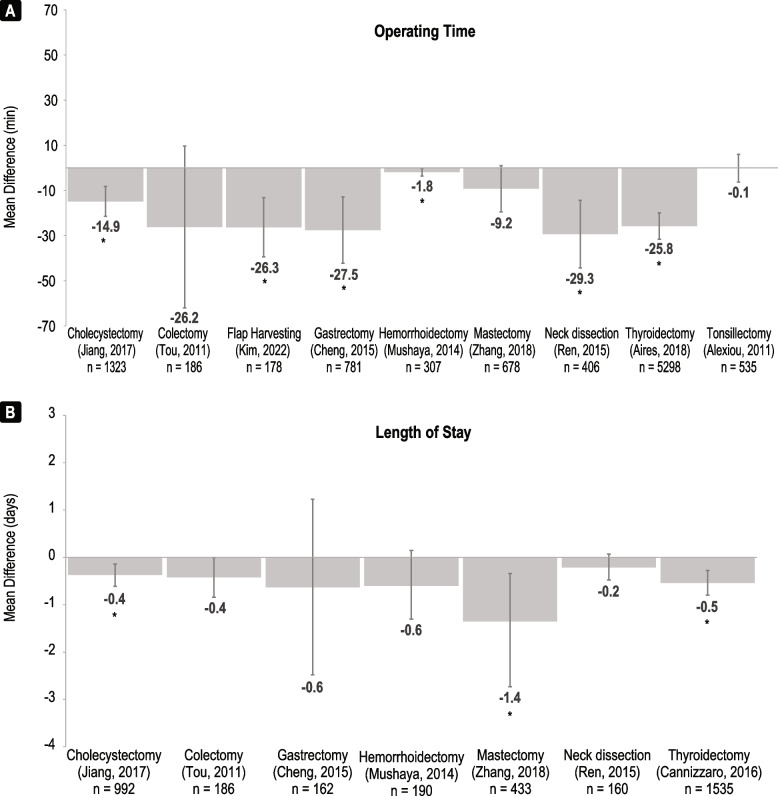
Summary of (**A**) operating time and (**B**) length of stay meta-analyses comparing Harmonic devices to conventional comparators. Mean difference is defined as (Harmonic value – comparator value). The n-values are n patients included. Asterisks (*) indicate a significant association (*p* < 0.05)

Operating time was reported for all nine procedures that compared Harmonic and conventional methods with an included systematic review (Fig. 3A, Supplementary Table 7). Harmonic devices were associated with reductions in operating time across all nine systematic reviews ranging from -0.10 min to -29.29 min compared to conventional methods [15, 17, 20, 23, 24, 27, 35, 37, 38]. A statistically significant reduction in operating time was reported for cholecystectomy [27], flap harvesting [24], gastrectomy [20], hemorrhoidectomy [17], neck dissection [38], and thyroidectomy [35]. Both systematic reviews showed lower operating time with Harmonic than ABP device comparators, but differences were not significant (Table 2) [15, 16].

Fifty of 60 additional RCTs that were not captured in the published meta-analyses reported similar trends to the meta-analyses with lower operating time with Harmonic than conventional techniques (Supplementary Table 2). A significant reduction in operating time was associated with Harmonic devices across 33 RCTs including three ABP device comparators and 35 conventional technique comparators (some RCTs had multiple comparators). Often, cholecystectomy [39–42], colectomy [43], hemorrhoidectomy [44–48], mastectomy [49–51], thyroidectomy [7, 8, 52–65], and tonsillectomy [66–68] RCTs demonstrated significantly shorter operating time with Harmonic devices compared to conventional techniques or ABP devices. Significantly longer operating time was demonstrated with Harmonic devices in only a minority of the RCTs that were not captured in the SLRs [7, 65].

All twenty-six orphan RCTs reported operating time (Supplementary Table 3). Seven RCTs reported operating times that were significantly shorter for Harmonic devices compared to conventional techniques including those focused on appendectomy [69], hepatectomy [70], uterine myomectomy [71], parathyroidectomy [72], and radial artery harvesting [73]. Two RCTs had significantly shorter operating times for comparator devices (one versus conventional techniques and one versus ABP devices) compared to Harmonic for appendectomy [74] and breast reduction procedures [75].

#### Length of stay

Seven systematic reviews reported the length of stay (LOS) after an operation that compared Harmonic and conventional methods (Fig. 3B, Supplementary Table 7). Harmonic devices were associated with a reduction in LOS in all seven systematic reviews ranging from -0.01 days to -1.35 days [15–17, 20, 23, 27, 38], with results being statistically significant for cholecystectomy [27], mastectomy [23], and thyroidectomy [35]. Both systematic reviews for colectomy and thyroidectomy that compared Harmonic to ABP devices showed no significant differences for LOS (Table 2) [15, 16].

LOS was reported by 33 of the additional RCTs (Supplementary Table 2). A significant reduction in LOS was observed with Harmonic devices across eight RCTs including one ABP device and seven conventional technique comparators. Cholecystectomy [40] and thyroidectomy [53, 56, 61, 63, 64, 76] RCTs demonstrated significantly shorter LOS for Harmonic devices compared to conventional techniques. Harmonic also demonstrated significantly reduced LOS compared to ABP devices for thyroidectomy [60]. None of the additional RCTs showed a significant increase in LOS for Harmonic devices.

Fourteen orphan RCTs reported on LOS (Supplementary Table 3). RCTs on hepatectomy [77] and uterine myomectomy [71] reported significantly shorter LOS following surgery with a Harmonic device compared to conventional techniques, while an RCT on appendectomy [74] reported a significantly shorter LOS for comparator ABP devices.

#### Intraoperative blood loss

**Fig. 4:**
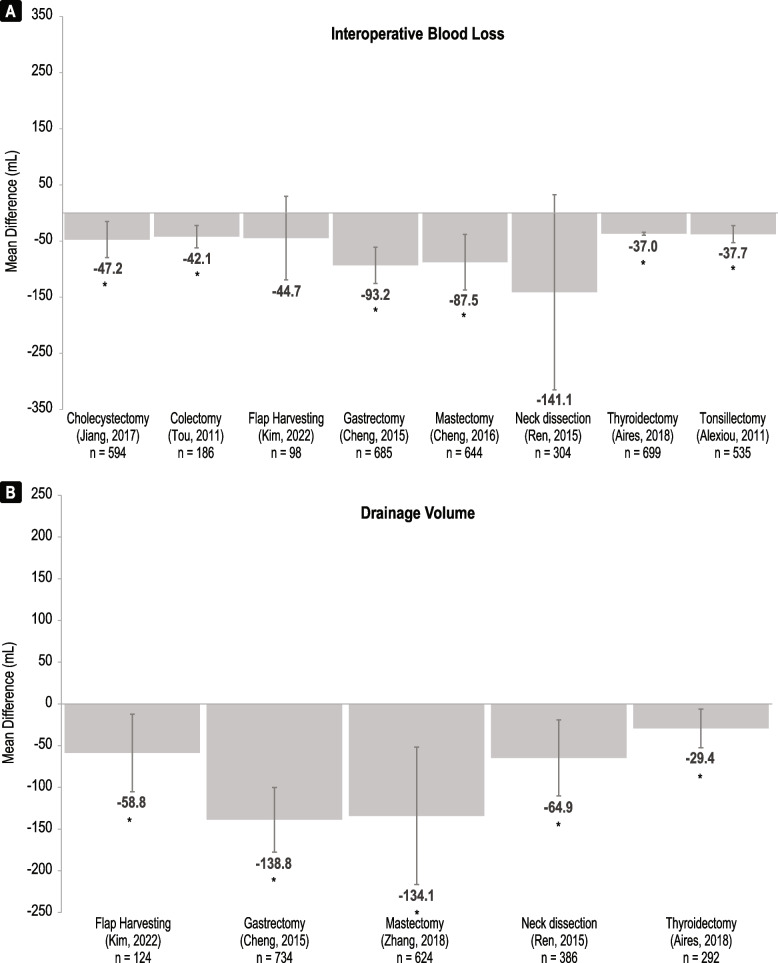
Summary of (**A**) intraoperative blood loss and (**B**) drainage volume meta-analyses comparing Harmonic devices to conventional comparators. Mean difference is defined as (Harmonic value – comparator value). The n-values are n patients included. Asterisks (*) indicate a significant association (*p* < 0.05)

Intraoperative blood loss was reported by systematic reviews across eight procedures that compared Harmonic and conventional methods (Fig. 4A, Supplementary Table 7). Harmonic devices were associated with a reduction in blood loss across all eight systematic reviews, ranging from -3.22 mL to -141.13 mL [15, 20, 23, 24, 27, 35, 37, 38]. A statistically significant reduction in intraoperative blood loss for Harmonic compared to conventional was reported for cholecystectomy [27], colectomy [15], gastrectomy [20], mastectomy [23], thyroidectomy [35], and tonsillectomy [37]. Both systematic reviews that compared Harmonic with ABP devices showed lower intraoperative blood loss with Harmonic, but differences were not significant (Table 2) [15, 16].

Interoperative blood loss was reported by 27 of the additional RCTs (Supplementary Table 2). A significant reduction in intraoperative blood loss was associated with Harmonic devices across 18 RCTs including 21 conventional technique comparators. Cholecystectomy [39, 41, 42], gastrectomy [78], mastectomy [49, 50, 79–81], neck dissection [82, 83], thyroidectomy [52, 56, 58, 63, 84], and tonsillectomy [66, 67], RCTs demonstrated significantly lower intraoperative blood loss with Harmonic devices compared to conventional techniques. Significantly higher intraoperative blood loss was associated with Harmonic devices compared to conventional techniques in one hemorrhoidectomy RCT [85]. None of the additional RCTs that compared Harmonic and ABP devices reported intraoperative blood loss.

Fifteen orphan RCTs reported on intraoperative blood loss (Supplementary Table 3). Six orphan RCTs reported intraoperative blood loss that was significantly lower for Harmonic devices compared to conventional techniques, including those focused on appendectomy [69], hepatectomy [77], hysterectomy [86], uterine myomectomy [71], parathyroidectomy [72], and oral surgery [87]. Only one RCT, focused on abdominoplasty, showed significantly lower intraoperative blood loss with conventional techniques compared to Harmonic [88]. There were two orphan RCTs that compared intraoperative blood loss between Harmonic and ABP devices for prostatectomy [89] and thoracoscopic lobectomy [90], but neither reported significant differences.

#### Drainage volume

Drainage volume was reported by systematic reviews across five procedure types that compared Harmonic and conventional methods (Fig. 4B, Supplementary Table 7). Harmonic devices were associated with a reduction in drainage volume across all five systematic reviews ranging from -29.38 to -138.83 mL [20, 23, 24, 35, 38]. A statistically significant reduction in drainage volume with Harmonic compared to conventional methods was reported for all five systematic reviews including flap harvesting [24], gastrectomy [20], mastectomy [23], neck dissection [38], and thyroidectomy [35]. Neither of the systematic reviews that compared Harmonic with ABP devices reported drainage volume outcomes [15, 16].

Drainage volume was reported by 24 of the additional RCTs (Supplementary Table 2). A significant reduction in drainage volume was associated with Harmonic devices across 14 RCTs including 16 conventional technique comparators. Cholecystectomy [41], colectomy [43], mastectomy [49–51, 79, 80, 91], and thyroidectomy [53, 54, 56, 57, 61, 92] RCTs demonstrated significantly lower drainage volume with Harmonic devices compared to conventional techniques. Significantly higher drainage volume was associated with Harmonic devices compared to an ABP device in one thyroidectomy RCT [93].

Seven orphan RCTs reported on drainage volume (Supplementary Table 3). Two orphan RCTs, focused on parathyroidectomy [72] and radial artery harvesting [73], reported a significant reduction in drainage volume associated with Harmonic devices, compared to conventional techniques, while none reported a significant reduction for conventional techniques or ABP devices compared to Harmonic.

#### Pain

**Fig. 5:**
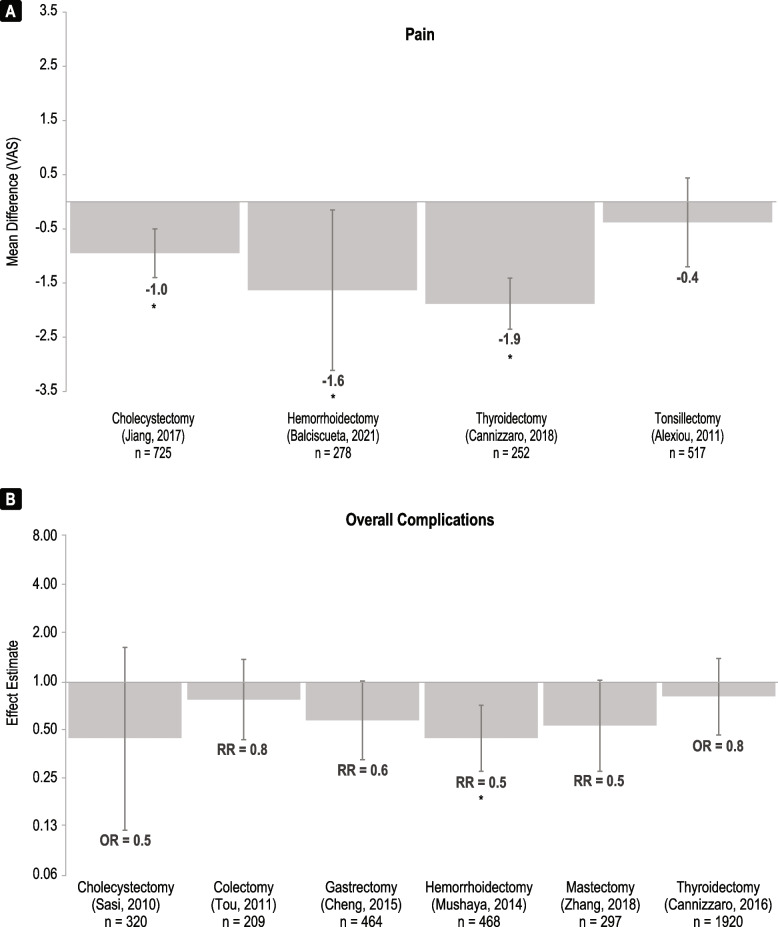
Summary of (**A**) pain and (**B**) overall complications meta-analyses comparing Harmonic devices to conventional comparators. Mean difference is defined as (Harmonic value – comparator value) and relative risk is defined as (Harmonic value / comparator value) . The n-values are n patients included. Asterisks (*) indicate a significant association (*p* < 0.05). *Abbreviations*: *OR* odds ratio, *RR* relative risk, *VAS *visual analogue scale

Pain was reported using the visual analogue scale by systematic reviews across four procedure types that compared Harmonic and conventional methods (Fig. 5A, Supplementary Table 7). Harmonic devices were associated with a reduction in pain in all four systematic reviews, with mean differences ranging from -0.38 to -1.88 [16, 18, 27, 37]. A statistically significant reduction in pain was reported for cholecystectomy [27], hemorrhoidectomy [17], and thyroidectomy [35]. Neither of the systematic reviews that compared Harmonic with ABP devices reported meta-analyses of more than one study for pain outcomes [15, 16].

Pain was reported by 21 of the additional RCTs (Supplementary Table 2). A significant reduction in pain was associated with Harmonic devices across five RCTs including five conventional technique comparators. Cholecystectomy [94], hemorrhoidectomy [46, 47], neck dissection [95], and tonsillectomy [96] RCTs demonstrated significantly lower pain with Harmonic devices compared to conventional techniques. Only one of the additional RCTs showed significantly higher pain for Harmonic devices compared to conventional techniques in hemorrhoidectomy [45].

Five orphan RCTs reported on pain (Supplementary Table 3). Two RCTs, focused on uterine myomectomy [71] and parathyroidectomy [72], reported a significant reduction in pain associated with Harmonic devices, compared to conventional techniques, while none reported a significant reduction for conventional techniques or ABP devices compared to Harmonic.

#### Overall complications

Overall complications were reported by systematic reviews across six procedure types that compared Harmonic and conventional methods (Fig. 5B, Supplementary Table 7). Harmonic devices were associated with a lower odds ratio or relative risk compared to conventional in all six systematic reviews, ranging from 0.82 to 0.45 [15–17, 20, 23, 25]. A statistically significant reduction was reported for hemorrhoidectomy [17]. The systematic reviews that compared Harmonic with ABP devices showed lower overall complications for colectomy but higher complications for thyroidectomy, but neither difference was significant (Table 2) [15, 16].

Overall complications were reported by eight of the additional RCTs (Supplementary Table 2). In one RCT focused on colectomy, a significant reduction in overall complications was associated with Harmonic devices compared to conventional techniques [43]. None of the additional RCTs showed a significant increase in overall complications for Harmonic devices.

Five orphan RCTs reported on overall complications (Supplementary Table 3). Two RCTs, on hepatectomy [77] and parenchymal liver transection [97], reported a significant reduction in overall complications associated with Harmonic devices, compared to conventional techniques, while none reported a significant reduction for comparator techniques or devices compared to Harmonic.

## Discussion

This umbrella review evaluated surgical outcomes for Harmonic devices across 24 SLRs and 83 additional RCTs above and beyond these SLRs. Both conventional techniques and ABP device comparators were included to provide a comprehensive overview of the highest-level evidence for surgical outcomes with Harmonic devices. Given the large volume of SLRs spanning nine procedure types, the most comprehensive review for each procedure was evaluated and described in-depth. Six outcomes were evaluated, and 39 effect estimates for different outcome types comparing Harmonic devices to conventional techniques were available. All effect estimates for every outcome studied showed numerical improvements associated with the use of Harmonic devices, with the majority (24 estimates) significantly favoring Harmonic. The volume of evidence comparing Harmonic to ABP devices was remarkably lower and results were generally similar between these comparators. Outside of the SLRs, the inclusion of 83 additional RCTs provide a comprehensive overview of nearly all high-quality evidence available to date for Harmonic devices. In general, results from the more recently published RCTs aligned with those from the SLRs, for procedures that were evaluated in SLRs for both conventional technique and ABP device comparators.

AMSTAR-2 and GRADE assessments were also employed to critically assess the methodological quality and certainty of evidence of the included studies. GRADE assessments were mostly moderate and low with some high and very low ratings. Almost all the most comprehensive SLRs and meta-analyses summarized in this review were moderate or low evidence certainty with only two exceptions being very low [16, 37]. The maturity of Harmonic technology and associated volume of studies allows for robust certainty of evidence for many outcomes across procedure types as determined using GRADE. For the AMSTAR-2 assessment many of the SLRs and meta-analyses were missing at least some components identified as important for complete systematic reviews. Regarding adjusting for heterogeneity (question 11), none of the studies included these sensitivity analyses. Heterogeneity is often a problem in meta-analyses and sometime low samples sizes preclude more restrictive analysis to adjust for these issues. A potential source for this may be surgical RCT design and may be difficult to control, however, future meta-analyses should include these sensitivities where possible. PRISMA reporting guidelines are evolving [11, 98] and assessment requirements are becoming more detailed with AMSTAR-2 including 16 questions compared to the original version with only 11 [13, 99]. The most critical issues relate to comprehensive searches and appropriate analysis methods, but systematic reviews can easily be docked for minor omissions such not explicitly stating why RCTs alone were the focus or not including a list of excluded studies. Low ratings on AMSTAR-2 should be interpreted with caution because they do not speak to the validity of the analyses and certainty of evidence but rather the completeness of reporting. The NICE checklist assessments showed that the 83 included RCTs were generally good quality with issues regarding reporting of allocation concealment and randomization in some studies. The key issue identified was blinding which was inadequate because it is not possible to blind physicians in surgical or medical device RCTs; blinding of patients and outcome assessors is seldomly reported in the Harmonic literature.

### Operating time

Operating time was the most studied outcome across the systematic review and RCTs evaluated. Across procedure types, Harmonic devices showed reductions, often close to 30 min, compared with conventional techniques. In comparison to ABP devices, there was a trend toward improved operating time with Harmonic devices in thyroidectomy. Reductions in operating time with Harmonic devices could be attributed with combined hemostasis, dissection, and cutting with a single instrument and reduced instrument exchanges [100]. Also, higher temperatures associated with electrosurgical devices produce smoke that can reduce surgical field visibility, whereas Harmonic devices operate at lower temperatures, thus producing less smoke [100–103]. Notably, the operating time for tonsillectomy was essentially the same for Harmonic devices and conventional techniques based on one available SLR that was published in 2011 [37]. Six additional tonsillectomy RCTs comparing Harmonic devices to conventional techniques that included operating time have since been published. Three RCTs reported significantly shorter operating times with Harmonic devices [66–68], one reported statistically significant shorter time with conventional techniques [104], and one reported no difference in operating time [105]. While these recent data seem to show that Harmonic devices are usually associated with shorter operating times, tonsillectomy is a quick procedure and the potential for reduced operating time is lower in magnitude relative to more time-consuming procedures. The Mushaya et al., 2014 study showed a 1.8 min significantly lower OR time with Harmonic than conventional techniques for hemorrhoidectomy [17]. This difference is relatively small compared to some of the reductions shown in other specialties and may not be very impactful. The time difference illustrates that using Harmonic is not slower than conventional hemorrhoidectomy and may be cost neutral from an OR time perspective. However, hemorrhoidectomy is a relatively short procedure, and a 1.8 min difference represents a 6.5% reduction in OR time based on the average OR time of the conventional technique studies included of 27.58 min. Additionally, small time savings across multiple procedures add up and can contribute to overall time and cost savings.

### Length of stay and pain

The consistent observations for reductions in pain and length of stay with Harmonic devices, across many procedure types, may be partially attributed to less thermal tissue damage associated with ultrasonic methods. Monopolar electrosurgical devices cut and coagulate using current to produce high temperatures (150ºC – 400ºC) that results in explosion of cells and subsequent hemostasis [106]. Conversely, Harmonic devices employ an end effector blade vibrating around 55 000 Hz across a range of 50 – 100 μm, producing frictional heat at much lower temperatures (50ºC – 100ºC) [4]. This heat is sufficient to break tertiary hydrogen bonds and induce protein denaturation, subsequently resulting in hemostasis. Lower heat may not be the only factor impacting length of stay as this outcome often varies by region and can be impacted by hospital policies regarding mandatory overnight stays, which can flatten potential differences between surgical methods. Indeed, the three procedure types where differences in length of stay significantly favored Harmonic compared to conventional techniques had SLRs with the largest sample sizes of the summarized effect estimates (cholecystectomy *n* = 992 [27], mastectomy *n* = 433 [23], and thyroidectomy *n* = 1 535 [16]). Despite this, all summarized SLRs showed directionally or significantly lower length of stay for Harmonic compared to conventional techniques. This was also the observation for pain outcomes. Most studies estimated less than one day of hospital stay saved, which may not make a large impact on a per patient basis, however, small differences can add up when considering procedures on an annual basis. For the specialties where the confidence intervals were wide, additional studies are required to determine whether there are indeed differences in hospital stay with Harmonic compared to conventional techniques.

### Harmonic versus ABP devices

In comparison to ABP devices, Harmonic devices generally showed similar outcomes for colectomy and thyroidectomy [15, 16]. Of note, the colectomy and thyroidectomy SLRs included only three (181 to 208 patients) and five (284 to 474 patients) RCTs comparing Harmonic to ABP devices, respectively. Given the modest evidence comparing these devices, as well as the variability in the type of ABP comparator it is difficult to draw conclusions regarding superiority of one device over another. However, both SLRs did report numerically reduced operating times associated with Harmonic devices [15, 16], a finding that has been well-substantiated versus conventional techniques. A potential explanation for this observation is that Harmonic devices combine hemostasis and cutting in a single instrument, whereas some earlier ABP devices are only used for hemostasis and do not have a cutting blade, which would necessitate time-consuming instrument exchange [100]. Conversely, in thyroidectomy, Harmonic devices were associated with a numerically increased rate of overall complications compared to ABP devices [16]. RCTs are often not powered to assess differences in complications given that the types of events vary and do not occur frequently. However, a recently published retrospective study investigating surgical outcomes between Harmonic and combination ABP/ultrasonic devices in thyroidectomy found that Harmonic devices were associated with significantly fewer cases of recurrent laryngeal nerve injury [107]. A possible reason for reduced laryngeal nerve injury may be lower thermal spread with Harmonic devices using algorithmic energy control [107]. Lower time on tissue with Harmonic than ABP/ultrasonic devices would result in less energy delivery to surrounding structures, which could also contribute to a lower frequency recurrent laryngeal nerve injury. Given these conflicting results, additional investigations should be conducted if there is a difference in the rate of complications associated with Harmonic and ABP devices.

### Orphan RCTs

Procedure types for which RCTs exist but no SLR has been conducted were also summarized in this umbrella review. In general, compared to conventional techniques, Harmonic devices were also associated with statistically significant or numerical improvements in surgical outcomes across these RCTs. The surgical specialty with the largest volume of RCTs, but never synthesized into a meta-analysis, was gynecological procedures including three on hysterectomy [86, 108, 109] and one on uterine myomectomy [71]. In addition, there were four RCTs comparing Harmonic devices to conventional techniques in various liver surgeries [70, 77, 97, 110]. For both gynecological and liver specialty areas, the only statistically significant differences that existed between outcomes including operating time [70, 71], length of stay [71, 77], intraoperative bleeding [71, 77, 86], pain [71], and overall complications [77, 97] showed benefit for Harmonic. As the number of RCTs evaluating gynecological and liver surgeries grow, SLRs and meta-analyses will be warranted.

### Comparison to the literature

To our knowledge, this is the second published umbrella review evaluating Harmonic devices. The first umbrella review was published in 2018 and focused on surgical oncology including breast, colon, gastric, and head and neck cancers [10]. The majority of SLRs included in the 2018 review showed statistically significant or numerical improvements in outcomes with the use of Harmonic devices compared with conventional techniques [10]. These finding align with the results of the current umbrella review which showed consistent improvements across all surgical outcomes and SLRs comparing Harmonic devices to conventional techniques, with the majority of associations being statistically significant. The 2018 review also performed rigorous quality assessments including AMSTAR and GRADE where studies received seven to ten out of a possible 11 “yes” answers for AMSTAR and of 41 outcomes assessed most received moderate to low GRADE certainty ratings. The distribution of GRADE certainty ratings was similar in this umbrella review compared to that of surgical oncology studies, but there was a lower proportion of high and very low ratings in this study [10]. While the 2018 umbrella review evaluated all SLRs across four surgical oncology types in detail [10], our current review examined only the most comprehensive SLRs spanning nine procedures. This approach ensures that RCTs included in multiple SLRs are not overrepresented, therefore skewing the results, and provides a more digestible overview of current high-quality evidence on surgical outcomes for Harmonic devices. Additionally, the current umbrella review included orphan RCTs for which an SLR has never been published. This approach increased the comprehensiveness of the review and allowed for the identification of procedure types where an SLR may be warranted, such as in liver and gynecological surgery. In general, the orphan RCTs showed the same trend in outcome benefits as the SLRs.

The focus of this study was on clinical and hospital resource use outcomes, which may have an impact on costs. Procedure costs associated with using Harmonic devices compared to conventional techniques were assessed by two systematic reviews, one on thyroidectomy alone [32] and another on a variety of surgeries (including gastrectomy, thyroidectomy, colectomy, cholecystectomy, Nissen fundoplication, and pancreaticoduodenectomy) [111]. In thyroidectomy, the use of Harmonic devices significantly reduced total procedure costs by approximately 10% ($229 USD per procedure, *P* = 0.007) compared to conventional techniques [32]. In the study evaluating a variety of surgeries, Harmonic devices were associated with a significant 8.7% reduction ($227.77 USD per procedure, *P* = 0.029) in costs relative to conventional techniques [111]. Additionally, a US hospital budget impact analysis showed cost savings of $101 USD per procedure when using Harmonic devices as a part of a portfolio of electrosurgery devices compared to other electrosurgery devices from multiple manufacturers [112]. Together these data show significant cost advantages for Harmonic devices compared to conventional techniques. While some evidence is available comparing Harmonic to ABP comparators [112], more comparative studies assessing hospital costs in additional specialties are required to assess potential cost differences and better inform electrosurgical device selection.

### Limitations

Umbrella reviews rely on the authors of the included SLRs to justifiably combine RCTs in meta-analyses, select appropriate statistical tests, and accurately report results. Errors in this process are difficult to identify while preparing an umbrella review, but potential biases were mitigated by performing AMSTAR-2 and GRADE assessments to critically assess the methodological quality and certainty of evidence of the included studies. The AMSTAR-2 assessments of the 24 SLRs and meta-analyses were consistently rated as critically low quality. Many of the SLRs were missing several minor components and none reported sensitivity analyses to address causes of heterogeneity. These data should be interpreted considering the AMSTAR-2 assessments, but this was balanced by focusing on the most comprehensive SLRs that achieved GRADE assessments that were generally moderate to low certainty for the outcomes assessed. Another limitation is the disproportionate distribution of SLRs across surgical procedures. For example, a considerable amount of evidence was available for cholecystectomy, gastrectomy, and thyroidectomy, whereas common procedures such as hysterectomy and liver surgery have never been summarized in an SLR. As such, these results may be more applicable to some procedures than others. Additionally, there was a lack of studies comparing Harmonic devices to ABP devices, making it difficult to draw definitive conclusions about efficacy and surgical outcomes. Given the increased popularity of Harmonic and ABP devices, future RCTs and SLRs should aim to compare these devices for procedure types for which they are both commonly used, controlling for heterogeneity in the type of ABP comparator. Also, this umbrella review did not stratify results by the Harmonic device model used which could increase heterogeneity due to differences in device accuracy and efficacy. However, this stratification would be difficult to achieve given that several SLRs also combined multiple different Harmonic devices in their analyses. Furthermore, RCTs synthesized in SLRs do not always specify the device models that were used or correctly report the brand name of the device used. Finally, heterogeneity was observed among several SLRs included in this umbrella review and can be attributed to a variety of factors. For example, differences in how each RCT defined the beginning and end of a surgery can have a significant impact on operating time [38]. Regional and local variation in hospital policy on length of stay could also impact results outside of the surgical methods used. Studies included in this review measured blood loss through various methods including weighing or squeezing out surgical sponges, measurements from the aspirator container, or surgeon’s appraisal of blood loss [23, 37, 38]. Drainage volume may vary depending on the location and number of drains placed, duration of drain placement, and variations in measurement methods [23, 24, 38]. Assessing the effect of these factors is challenging when synthesizing literature, but outcomes and assessment techniques should be defined as best as possible in study methods.

This umbrella review summarized and evaluated evidence on the use of Harmonic devices compared to conventional techniques and ABP devices. All procedure types for which an SLR was available were summarized and additional RCTs were also included to ensure a comprehensive overview of surgical outcomes associated with the use of Harmonic devices. AMSTAR-2 and GRADE assessments were performed to assess methodological quality and strength of evidence. Compared to conventional techniques, the use of Harmonic devices consistently resulted in improved operating time, length of stay, blood loss and drainage volume, pain, and complications across a wide breadth of procedure types. The volume of meta-analyses comparing Harmonic versus ABP devices is more limited than those comparing Harmonic versus conventional techniques, therefore, more studies on Harmonic versus ABP devices are warranted so that comparisons for additional specialties can be made. The summary of evidence presented in this review may help clinicians, health economists, and hospital procurement personnel make evidenced-based decisions regarding surgical device selection.

## Supplementary Information

**Additional file 1.**

## Data Availability

The data used to prepare this manuscript are available in the supplementary materials. Additional information is available upon request from the Corresponding author.
