# Supplementary material for: An umbrella review of the surgical performance of Harmonic ultrasonic devices and impact on patient outcomes
Source: BMC Surg. 2023 Jun 29;23:180. doi: 10.1186/s12893-023-02057-9 (PMC10308659; doi:10.1186/s12893-023-02057-9)
Supplement: Supplementary file 1 — Additional file 1. [file 12893_2023_2057_MOESM1_ESM.docx]

An umbrella review of the surgical performance of Harmonic ultrasonic devices and impact on patient outcomes

Authors: Robert Kloosterman^1^, George W.J. Wright^1^, Elizabeth M. Salvo-Halloran^1^, Nicole C. Ferko^1^, John Z. Mennone^2^, Jeffrey W. Clymer^2^, Crystal D. Ricketts^2^, Giovanni A. Tommaselli^2^

Author Affiliations:

^1^EVERSANA, Burlington, ON, Canada

^2^Ethicon, Inc., Cincinnati, OH, USA

Corresponding Author: George Wright, EVERSANA, 204-3228 South Service Rd., Burlington, ON, L7N 3H8 [george.wright@eversana.com](mailto:george.wright@eversana.com) ORCID ID: **0000-0002-7089-1203**

**Supplementary Materials - Index**

| **Supplementary Appendixes** |  |
| --- | --- |
| Appendix A1: Search Strategy for January 31, 2022 search | *pag. 2* |
| Appendix A2: Search Strategy for September 16, 2019 search | *pag. 7* |
| Appendix A3: Search Strategy for June 2, 2018 search | *pag. 13* |
| Appendix A4: Search Strategy for October 1, 2016 search | *pag. 19* |
| **Supplementary Tables** |  |
| Supplementary Table 1: PRISMA checklist | *pag. 29* |
| Supplementary Table 2: Summary of additional RCTs | *pag. 31* |
| Supplementary Table 3: Summary of included orphan RCTs | *pag. 34* |
| Supplementary Table 4: Methodological quality assessment of included systematic reviews using AMSTAR-2 | *pag. 36* |
| Supplementary Table 5: Grade assessment for included meta-analyses | *pag. 38* |
| Supplementary Table 6: NICE Checklist (2022) Assessment of Included RCTs | *pag. 52* |
| Supplementary Table 7: Surgical outcomes from most comprehensive SLRs reporting on Harmonic versus conventional | *pag. 55* |
| **References** | *pag. 57* |
|  |  |

1. Supplementary Appendixes
   1. Search strategy for January 31, 2022, search

**MULTIFILE SEARCH**

Database(s): **EBM Reviews - Cochrane Central Register of Controlled Trials**December 2021**, EBM Reviews - Cochrane Database of Systematic Reviews**2005 to January 26, 2022**, Embase**1974 to 2022 January 28**, Ovid MEDLINE(R) and Epub Ahead of Print, In-Process, In-Data-Review & Other Non-Indexed Citations and Daily**1946 to January 28, 2022
Search Strategy:

| **#** | **Searches** | **Results** |
| --- | --- | --- |
| 1 | (harmonic* adj2 (blade? or dissect* or hook? or incis* or scalpel? or shear? or scalpel?)).tw,kw. | 3366 |
| 2 | (ultrasonic* adj2 (blade? or dissect* or hook? or incis* or scalpel? or shear? or scalpel?)).tw,kw. | 3116 |
| 3 | (ultrasound* adj2 (blade? or dissect* or hook? or incis* or scalpel? or shear? or scalpel?)).tw,kw. | 2295 |
| 4 | ultracision?.tw,kw. | 473 |
| 5 | ultra-cision?.tw,kw. | 7 |
| 6 | (harmonic* adj (ACE* or Focus* or Synergy* or Wave*)).tw,kw. | 1315 |
| 7 | "ACE+ 7".tw,kw. | 39 |
| 8 | "Harmonic 7".tw,kw. | 4 |
| 9 | "CS 14-C".tw,kw. | 7 |
| 10 | ("HD 1000i Shears" or "HD 1100i Shears").tw,kw. | 0 |
| 11 | HF005.tw,kw. | 1 |
| 12 | Ultrasonic Surgical Procedures/ | 1050 |
| 13 | ((ultrason* or ultrasound*) adj2 surg* adj3 (device* or instrument* or procedur* or technique*)).tw,kw. | 627 |
| 14 | or/1-13 | 10718 |
| 15 | exp Animals/ not (exp Animals/ and Humans/) | 17071157 |
| 16 | 14 not 15 | 8059 |
| 17 | (comment or editorial or interview or news or newspaper article).pt. | 2313847 |
| 18 | (letter not (letter and randomized controlled trial)).pt. | 2371562 |
| 19 | 16 not (17 or 18) | 7920 |
| 20 | limit 19 to systematic reviews [Limit not valid in CCTR,CDSR,Embase; records were retained] | 4462 |
| 21 | meta analysis.pt. | 152333 |
| 22 | exp meta-analysis as topic/ | 72242 |
| 23 | (meta-analy* or metanaly* or metaanaly* or met analy* or integrative research or integrative review* or integrative overview* or research integration or research overview* or collaborative review*).tw,kw. | 560732 |
| 24 | (systematic review* or systematic overview* or evidence-based review* or evidence-based overview* or (evidence adj3 (review* or overview*)) or meta-review* or meta-overview* or meta-synthes* or rapid review* or "review of reviews" or technology assessment* or HTA or HTAs).tw,kw. | 669776 |
| 25 | exp Technology assessment, biomedical/ | 27463 |
| 26 | (cochrane or health technology assessment or evidence report).jw. | 56513 |
| 27 | ((indirect* or mixed or multi-treatment*) adj2 compar*).tw,kw. | 18089 |
| 28 | ((network* or network-based) adj (MA or MAs)).kw,tw. | 52 |
| 29 | or/21-28 | 1070057 |
| 30 | 19 and 29 | 192 |
| 31 | 20 or 30 [SYSTEMATIC REVIEWS] | 4497 |
| 32 | (controlled clinical trial or randomized controlled trial).pt. | 1278336 |
| 33 | clinical trials as topic.sh. | 232329 |
| 34 | exp Randomized Controlled Trials as Topic/ | 383629 |
| 35 | (randomi#ed or randomly or RCT$1 or placebo*).tw,kw. | 3880334 |
| 36 | ((singl* or doubl* or trebl* or tripl*) adj (mask* or blind* or dumm*)).tw,kw. | 783547 |
| 37 | trial.ti. | 975860 |
| 38 | or/32-37 | 4773505 |
| 39 | 19 and 38 [RCTs] | 1491 |
| 40 | controlled clinical trial.pt. | 187559 |
| 41 | Controlled Clinical Trial/ or Controlled Clinical Trials as Topic/ | 575744 |
| 42 | (control* adj2 trial*).tw,kw. | 1440830 |
| 43 | or/40-42 | 2026843 |
| 44 | 19 and 43 [NON-RCTs] | 704 |
| 45 | 31 or 39 or 44 [ALL STUDY DESIGNS] | 4884 |
| 46 | (201909* or 201910* or 201911* or 201912* or 2020* or 2021*).dt. | 3459428 |
| 47 | 45 and 46 [UPDATE PERIOD] | 47 |
| 48 | limit 47 to yr="2019 -Current" | 47 |
| 49 | 48 use ppez | 47 |
| 50 | harmonic.dv,dm,my. | 899 |
| 51 | (harmonic* adj2 (blade? or dissect* or hook? or incis* or scalpel? or shear? or scalpel?)).tw,kw. | 3366 |
| 52 | (ultrason* adj2 (blade? or dissect* or hook? or incis* or scalpel? or shear? or scalpel?)).tw,kw. | 3457 |
| 53 | (ultrasound* adj2 (blade? or dissect* or hook? or incis* or scalpel? or shear? or scalpel?)).tw,kw. | 2295 |
| 54 | ultracision?.tw,kw. | 473 |
| 55 | ultra-cision?.tw,kw. | 7 |
| 56 | (harmonic* adj (ACE* or Focus* or Synergy* or Wave*)).tw,kw,dv,dm,my. | 1529 |
| 57 | "ACE+ 7".tw,kw,dv,dm,my. | 48 |
| 58 | "Harmonic 7".tw,kw,dv,dm,my. | 4 |
| 59 | "CS 14-C".tw,kw,dv,dm,my. | 7 |
| 60 | ("HD 1000i Shears" or "HD 1100i Shears").tw,kw,dv,dm,my. | 1 |
| 61 | HF005.tw,kw,dv,dm,my. | 1 |
| 62 | ultrasound surgery/ | 561 |
| 63 | ((ultrason* or ultrasound*) adj2 surg* adj3 (device* or instrument* or procedur* or technique*)).tw,kw. | 627 |
| 64 | or/50-63 | 11293 |
| 65 | exp animal experimentation/ or exp models animal/ or exp animal experiment/ or nonhuman/ or exp vertebrate/ | 54605942 |
| 66 | exp human/ or exp human experimentation/ or exp human experiment/ | 43951019 |
| 67 | 65 not 66 | 10656711 |
| 68 | 64 not 67 | 10493 |
| 69 | editorial.pt. | 1309331 |
| 70 | letter.pt. not (letter.pt. and randomized controlled trial/) | 2371477 |
| 71 | 68 not (69 or 70) | 10314 |
| 72 | meta-analysis/ | 387636 |
| 73 | "systematic review"/ | 513113 |
| 74 | "meta analysis (topic)"/ | 48101 |
| 75 | (meta-analy* or metanaly* or metaanaly* or met analy* or integrative research or integrative review* or integrative overview* or research integration or research overview* or collaborative review*).tw,kw. | 560732 |
| 76 | (systematic review* or systematic overview* or evidence-based review* or evidence-based overview* or (evidence adj3 (review* or overview*)) or meta-review* or meta-overview* or meta-synthes* or rapid review* or "review of reviews" or technology assessment* or HTA or HTAs).tw,kw. | 669776 |
| 77 | biomedical technology assessment/ | 26337 |
| 78 | (cochrane or health technology assessment or evidence report).jw. | 56513 |
| 79 | ((indirect* or mixed or multi-treatment*) adj2 compar*).tw,kw. | 18089 |
| 80 | ((network* or network-based) adj (MA or MAs)).kw,tw. | 52 |
| 81 | or/72-80 | 1173448 |
| 82 | 71 and 81 [SYSTEMATIC REVIEWS] | 285 |
| 83 | randomized controlled trial/ or controlled clinical trial/ | 1529222 |
| 84 | exp "clinical trial (topic)"/ | 379891 |
| 85 | (randomi#ed or randomly or RCT$1 or placebo*).tw,kw. | 3880334 |
| 86 | ((singl* or doubl* or trebl* or tripl*) adj (mask* or blind* or dumm*)).tw,kw. | 783547 |
| 87 | trial.ti. | 975860 |
| 88 | or/83-87 | 4810965 |
| 89 | 71 and 88 [RCTs] | 1817 |
| 90 | exp controlled clinical trial/ | 1531626 |
| 91 | exp "controlled clinical trial (topic)"/ | 227623 |
| 92 | (control* adj2 trial*).tw,kw. | 1440830 |
| 93 | or/90-92 | 2778137 |
| 94 | 71 and 93 [non-RCTs] | 1196 |
| 95 | 82 or 89 or 94 [ALL STUDY DESIGNS] | 1951 |
| 96 | (201909* or 201910* or 201911* or 201912* or 2020* or 2021*).dc. | 4585969 |
| 97 | 95 and 96 [UPDATE PERIOD] | 96 |
| 98 | limit 97 to yr="2019 -Current" | 88 |
| 99 | 98 use oemezd | 88 |
| 100 | (harmonic* adj2 (blade? or dissect* or hook? or incis* or scalpel? or shear? or scalpel?)).ti,ab,kw. | 3356 |
| 101 | (ultrasonic* adj2 (blade? or dissect* or hook? or incis* or scalpel? or shear? or scalpel?)).ti,ab,kw. | 3106 |
| 102 | (ultrasound* adj2 (blade? or dissect* or hook? or incis* or scalpel? or shear? or scalpel?)).ti,ab,kw. | 2291 |
| 103 | ultracision?.ti,ab,kw. | 468 |
| 104 | ultra-cision?.ti,ab,kw. | 7 |
| 105 | (harmonic* adj (ACE* or Focus* or Synergy* or Wave*)).ti,ab,kw. | 1314 |
| 106 | "ACE+ 7".ti,ab,kw. | 39 |
| 107 | "Harmonic 7".ti,ab,kw. | 4 |
| 108 | "CS 14-C".ti,ab,kw. | 7 |
| 109 | ("HD 1000i Shears" or "HD 1100i Shears").ti,ab,kw. | 0 |
| 110 | HF005.ti,ab,kw. | 1 |
| 111 | Ultrasonic Surgical Procedures/ | 1050 |
| 112 | ((ultrason* or ultrasound*) adj2 surg* adj3 (device* or instrument* or procedur* or technique*)).ti,ab,kw. | 621 |
| 113 | or/100-112 | 10690 |
| 114 | (201908* or 201909* or 201910* or 201911* or 201912* or 2020* or 2021*).up. | 40004095 |
| 115 | 113 and 114 [UPDATE PERIOD] | 5367 |
| 116 | limit 115 to yr="2019 -Current" | 1610 |
| 117 | 116 use cctr | 158 |
| 118 | (harmonic* adj2 (blade? or dissect* or hook? or incis* or scalpel? or shear? or scalpel?)).ti,ab,kw. | 3356 |
| 119 | (ultrasonic* adj2 (blade? or dissect* or hook? or incis* or scalpel? or shear? or scalpel?)).ti,ab,kw. | 3106 |
| 120 | (ultrasound* adj2 (blade? or dissect* or hook? or incis* or scalpel? or shear? or scalpel?)).ti,ab,kw. | 2291 |
| 121 | ultracision?.ti,ab,kw. | 468 |
| 122 | ultra-cision?.ti,ab,kw. | 7 |
| 123 | (harmonic* adj (ACE* or Focus* or Synergy* or Wave*)).ti,ab,kw. | 1314 |
| 124 | "ACE+ 7".ti,ab,kw. | 39 |
| 125 | "Harmonic 7".ti,ab,kw. | 4 |
| 126 | "CS 14-C".ti,ab,kw. | 7 |
| 127 | ("HD 1000i Shears" or "HD 1100i Shears").ti,ab,kw. | 0 |
| 128 | HF005.ti,ab,kw. | 1 |
| 129 | ((ultrason* or ultrasound*) adj2 surg* adj3 (device* or instrument* or procedur* or technique*)).ti,ab,kw. | 621 |
| 130 | or/118-129 | 9936 |
| 131 | (201909* or 201910* or 201911* or 201912* or 2020* or 2021*).up. | 39995148 |
| 132 | 130 and 131 [UPDATE PERIOD] | 5012 |
| 133 | limit 132 to yr="2019 -Current" | 1523 |
| 134 | 133 use coch | 0 |
| 135 | 49 or 99 or 117 or 134 [All Databases] | 293 |
| **136** | **remove duplicates from 135** | **218** |

- 1. Search Strategy for September 16, 2019, search

MEDLINE

Database: Ovid MEDLINE(R) and Epub Ahead of Print, In-Process & Other Non-Indexed Citations and Daily <1946 to September 13, 2019>

Search Strategy:

--------------------------------------------------------------------------------

1 (harmonic* adj2 (blade? or dissect* or hook? or incis* or scalpel? or shear? or scalpel?)).tw,kw. (1012)

2 (ultrasonic* adj2 (blade? or dissect* or hook? or incis* or scalpel? or shear? or scalpel?)).tw,kw. (1037)

3 (ultrasound* adj2 (blade? or dissect* or hook? or incis* or scalpel? or shear? or scalpel?)).tw,kw. (602)

4 ultracision?.tw,kw. (133)

5 ultra-cision?.tw,kw. (1)

6 (harmonic* adj (ACE* or Focus* or Synergy* or Wave*)).tw,kw. (541)

7 ""ACE+ 7"".tw,kw. (13)

8 ""Harmonic 7"".tw,kw. (2)

9 ""CS 14-C"".tw,kw. (1)

10 ""HD 1000i Shears"".tw,kw. (0)

11 HF005.tw,kw. (1)

12 Ultrasonic Surgical Procedures/ (373)

13 ((ultrason* or ultrasound*) adj2 surg* adj3 (device* or instrument* or procedur* or technique*)).tw,kw. (168)

14 or/1-13 (3340)

15 exp Animals/ not (exp Animals/ and Humans/) (4615338)

16 14 not 15 (3018)

17 (comment or editorial or interview or news or newspaper article).pt. (1376103)

18 (letter not (letter and randomized controlled trial)).pt. (1037155)

19 16 not (17 or 18) (2940)

20 limit 19 to systematic reviews (27)

21 meta analysis.pt. (104506)

22 exp meta-analysis as topic/ (18180)

23 (meta-analy* or metanaly* or metaanaly* or met analy* or integrative research or integrative review* or integrative overview* or research integration or research overview* or collaborative review*).tw,kw. (159739)

24 (systematic review* or systematic overview* or evidence-based review* or evidence-based overview* or (evidence adj3 (review* or overview*)) or meta-review* or meta-overview* or meta-synthes* or rapid review* or ""review of reviews"" or technology assessment* or HTA or HTAs).tw,kw. (197411)

25 exp Technology assessment, biomedical/ (10800)

26 (cochrane or health technology assessment or evidence report).jw. (16013)

27 ((indirect* or mixed or multi-treatment*) adj2 compar*).tw,kw. (4699)

28 ((network* or network-based) adj (MA or MAs)).kw,tw. (7)

29 or/21-28 (328776)

30 19 and 29 (60)

31 20 or 30 [SYSTEMATIC REVIEWS] (60)

32 (controlled clinical trial or randomized controlled trial).pt. (577610)

33 clinical trials as topic.sh. (188368)

34 exp Randomized Controlled Trials as Topic/ (128909)

35 (randomi#ed or randomly or RCT$1 or placebo*).tw,kw. (923046)

36 ((singl* or doubl* or trebl* or tripl*) adj (mask* or blind* or dumm*)).tw,kw. (166222)

37 trial.ti. (204608)

38 or/32-37 (1341282)

39 19 and 38 [RCTs] (388)

40 controlled clinical trial.pt. (93251)

41 Controlled Clinical Trial/ or Controlled Clinical Trials as Topic/ (98658)

42 (control* adj2 trial*).tw,kw. (251237)

43 Non-Randomized Controlled Trials as Topic/ (529)

44 (nonrandom* or non-random* or quasi-random* or quasi-experiment*).tw,kw. (52649)

45 (nRCT or nRCTs or non-RCT$1).tw,kw. (801)

46 Controlled Before-After Studies/ (418)

47 (control* adj3 (""before and after"" or ""before after"")).tw,kw. (4077)

48 Interrupted Time Series Analysis/ (651)

49 (time series adj3 interrupt*).tw,kw. (2668)

50 (pre- adj3 post-).tw,kw. (76170)

51 (pretest adj3 posttest).tw,kw. (5123)

52 Historically Controlled Study/ (157)

53 (control* adj2 stud$3).tw,kw. (220840)

54 Control Groups/ (1629)

55 (control* adj2 group$1).tw,kw. (475194)

56 trial.ti. (204608)

57 or/40-56 (1151248)

58 19 and 57 [NON-RCTs] (264)

59 exp Cohort Studies/ (1897110)

60 cohort$1.tw,kw. (533643)

61 Retrospective Studies/ (769174)

62 (longitudinal or prospective or retrospective).tw,kw. (1181258)

63 ((followup or follow-up) adj (study or studies)).tw,kw. (48225)

64 Observational study.pt. (66800)

65 (observation$2 adj (study or studies)).tw,kw. (97284)

66 ((population or population-based) adj (study or studies or analys#s)).tw,kw. (22919)

67 ((multidimensional or multi-dimensional) adj (study or studies)).tw,kw. (108)

68 Comparative Study.pt. (1839329)

69 ((comparative or comparison) adj (study or studies)).tw,kw. (105377)

70 exp Case-Control Studies/ (1018163)

71 ((case-control* or case-based or case-comparison) adj (study or studies)).tw,kw. (100668)

72 or/59-71 (4310291)

73 19 and 72 [OBSERVATIONAL STUDIES] (1118)

74 31 or 39 or 58 or 73 [ALL STUDY DESIGNS] (1263)

75 (2018052* or 201806* or 201807* or 201808* or 201809* or 201810* or 201811* or 201812* or 2019*).dt. (1690227)

76 74 and 75 [UPDATE PERIOD] (89)

77 remove duplicates from 76 (89)

***************************

Embase

Database: Embase <1974 to 2019 September 13>

Search Strategy:

--------------------------------------------------------------------------------

1 harmonic.dv. (760)

2 (harmonic* adj2 (blade? or dissect* or hook? or incis* or scalpel? or shear? or scalpel?)).tw,kw. (1808)

3 (ultrason* adj2 (blade? or dissect* or hook? or incis* or scalpel? or shear? or scalpel?)).tw,kw. (1644)

4 (ultrasound* adj2 (blade? or dissect* or hook? or incis* or scalpel? or shear? or scalpel?)).tw,kw. (875)

5 ultracision?.tw,kw. (269)

6 ultra-cision?.tw,kw. (5)

7 (harmonic* adj (ACE* or Focus* or Synergy* or Wave*)).tw,kw. (487)

8 ""ACE+ 7"".tw,kw. (24)

9 ""Harmonic 7"".tw,kw. (1)

10 ""CS 14-C"".tw,kw. (0)

11 ""HD 1000i Shears"".tw,kw. (0)

12 HF005.tw,kw. (0)

13 ultrasound surgery/ (489)

14 ((ultrason* or ultrasound*) adj2 surg* adj3 (device* or instrument* or procedur* or technique*)).tw,kw. (232)

15 or/1-14 (5531)

16 exp animal experimentation/ or exp models animal/ or exp animal experiment/ or nonhuman/ or exp vertebrate/ (26059336)

17 exp human/ or exp human experimentation/ or exp human experiment/ (20162472)

18 16 not 17 (5897796)

19 15 not 18 (5127)

20 editorial.pt. (632109)

21 letter.pt. not (letter.pt. and randomized controlled trial/) (1081934)

22 19 not (20 or 21) (5030)

23 meta-analysis/ (171623)

24 ""systematic review""/ (219394)

25 ""meta analysis (topic)""/ (41160)

26 (meta-analy* or metanaly* or metaanaly* or met analy* or integrative research or integrative review* or integrative overview* or research integration or research overview* or collaborative review*).tw,kw. (213212)

27 (systematic review* or systematic overview* or evidence-based review* or evidence-based overview* or (evidence adj3 (review* or overview*)) or meta-review* or meta-overview* or meta-synthes* or rapid review* or ""review of reviews"" or technology assessment* or HTA or HTAs).tw,kw. (246520)

28 biomedical technology assessment/ (13777)

29 (cochrane or health technology assessment or evidence report).jw. (22487)

30 ((indirect* or mixed or multi-treatment*) adj2 compar*).tw,kw. (7648)

31 ((network* or network-based) adj (MA or MAs)).kw,tw. (16)

32 or/23-31 (502922)

33 22 and 32 [SYSTEMATIC REVIEWS] (127)

34 randomized controlled trial/ or controlled clinical trial/ (756756)

35 exp ""clinical trial (topic)""/ (307580)

36 (randomi#ed or randomly or RCT$1 or placebo*).tw,kw. (1303778)

37 ((singl* or doubl* or trebl* or tripl*) adj (mask* or blind* or dumm*)).tw,kw. (230711)

38 trial.ti. (281471)

39 or/34-38 (1848056)

40 22 and 39 [RCTs] (633)

41 exp controlled clinical trial/ (757181)

42 exp ""controlled clinical trial (topic)""/ (175743)

43 (control* adj2 trial*).tw,kw. (342071)

44 (nonrandom* or non-random* or quasi-random* or quasi-experiment*).tw,kw. (66529)

45 (nRCT or nRCTs or non-RCT$1).tw,kw. (1143)

46 (control* adj3 (""before and after"" or ""before after"")).tw,kw. (5352)

47 time series analysis/ (24048)

48 (time series adj3 interrupt*).tw,kw. (3384)

49 (pre- adj3 post-).tw,kw. (137594)

50 (pretest adj3 posttest).tw,kw. (5988)

51 controlled study/ (6946411)

52 (control* adj2 stud$3).tw,kw. (298526)

53 control group/ (110810)

54 (control* adj2 group$1).tw,kw. (685584)

55 trial.ti. (281471)

56 or/41-55 (7812963)

57 22 and 56 [NON-RCTs] (1326)

58 cohort analysis/ (508485)

59 cohort$1.tw,kw. (916352)

60 retrospective study/ (825782)

61 longitudinal study/ (130705)

62 prospective study/ (551332)

63 (longitudinal or prospective or retrospective).tw,kw. (1819760)

64 follow up/ (1457279)

65 ((followup or follow-up) adj (study or studies)).tw,kw. (65726)

66 observational study/ (178877)

67 (observation$2 adj (study or studies)).tw,kw. (155691)

68 population research/ (101912)

69 ((population or population-based) adj (study or studies or analys#s)).tw,kw. (23765)

70 ((multidimensional or multi-dimensional) adj (study or studies)).tw,kw. (139)

71 exp comparative study/ (1347913)

72 ((comparative or comparison) adj (study or studies)).tw,kw. (126665)

73 exp case control study/ (164255)

74 ((case-control* or case-based or case-comparison) adj (study or studies)).tw,kw. (132243)

75 or/58-74 (5172337)

76 22 and 75 [OBSERVATIONAL STUDIES] (2027)

77 33 or 40 or 57 or 76 [ALL STUDY DESIGNS] (2584)

78 (2018052* or 201806* or 201807* or 201808* or 201809* or 201810* or 201811* or 201812* or 2019*).dc. (2505562)

79 77 and 78 [UPDATE PERIOD] (364)

80 remove duplicates from 79 (364)

***************************

CENTRAL

Database: EBM Reviews - Cochrane Central Register of Controlled Trials <August 2019>

Search Strategy:

--------------------------------------------------------------------------------

1 (harmonic* adj2 (blade? or dissect* or hook? or incis* or scalpel? or shear? or scalpel?)).ti,ab,kw. (296)

2 (ultrasonic* adj2 (blade? or dissect* or hook? or incis* or scalpel? or shear? or scalpel?)).ti,ab,kw. (208)

3 (ultrasound* adj2 (blade? or dissect* or hook? or incis* or scalpel? or shear? or scalpel?)).ti,ab,kw. (100)

4 ultracision?.ti,ab,kw. (47)

5 ultra-cision?.ti,ab,kw. (1)

6 (harmonic* adj (ACE* or Focus* or Synergy* or Wave*)).ti,ab,kw. (69)

7 ""ACE+ 7"".ti,ab,kw. (2)

8 ""Harmonic 7"".ti,ab,kw. (0)

9 ""CS 14-C"".ti,ab,kw. (0)

10 ""HD 1000i Shears"".ti,ab,kw. (0)

11 HF005.ti,ab,kw. (0)

12 Ultrasonic Surgical Procedures/ (47)

13 ((ultrason* or ultrasound*) adj2 surg* adj3 (device* or instrument* or procedur* or technique*)).ti,ab,kw. (110)

14 or/1-13 (682)

15 (""201806"" or ""201807"" or ""201808"" or ""201809"" or ""201810"" or ""201811"" or ""201812"" or 2019*).up. (1170615)

16 14 and 15 [UPDATE PERIOD] (514)

***************************

Cochrane DSR

Database: EBM Reviews - Cochrane Database of Systematic Reviews <2005 to September 11, 2019>

Search Strategy:

--------------------------------------------------------------------------------

1 (harmonic* adj2 (blade? or dissect* or hook? or incis* or scalpel? or shear? or scalpel?)).ti,ab,kw. (2)

2 (ultrasonic* adj2 (blade? or dissect* or hook? or incis* or scalpel? or shear? or scalpel?)).ti,ab,kw. (1)

3 (ultrasound* adj2 (blade? or dissect* or hook? or incis* or scalpel? or shear? or scalpel?)).ti,ab,kw. (0)

4 ultracision?.ti,ab,kw. (0)

5 ultra-cision?.ti,ab,kw. (0)

6 (harmonic* adj (ACE* or Focus* or Synergy* or Wave*)).ti,ab,kw. (0)

7 ""ACE+ 7"".ti,ab,kw. (0)

8 ""Harmonic 7"".ti,ab,kw. (0)

9 ""CS 14-C"".ti,ab,kw. (0)

10 ""HD 1000i Shears"".ti,ab,kw. (0)

11 HF005.ti,ab,kw. (0)

12 [Ultrasonic Surgical Procedures/] (0)

13 ((ultrason* or ultrasound*) adj2 surg* adj3 (device* or instrument* or procedur* or technique*)).ti,ab,kw. (0)

14 or/1-13 (3)

15 (""201806"" or ""201807"" or ""201808"" or ""201809"" or ""201810"" or ""201811"" or ""201812"" or 2019*).up. (1266)

16 14 and 15 [UPDATE PERIOD] (0)

***************************"

- 1. Search Strategy for June 2, 2018, search

Harmonic Scalpel

Update

2018 Jun 2

MEDLINE

Database: Ovid MEDLINE(R) Epub Ahead of Print, In-Process & Other Non-Indexed Citations, Ovid MEDLINE(R) Daily and Ovid MEDLINE(R) <1946 to Present>

Search Strategy:

--------------------------------------------------------------------------------

1 (harmonic* adj2 (blade? or dissect* or hook? or incis* or scalpel? or shear? or scalpel?)).tw,kw. (979)

2 (ultrasonic* adj2 (blade? or dissect* or hook? or incis* or scalpel? or shear? or scalpel?)).tw,kw. (978)

3 (ultrasound* adj2 (blade? or dissect* or hook? or incis* or scalpel? or shear? or scalpel?)).tw,kw. (489)

4 ultracision?.tw,kw. (131)

5 ultra-cision?.tw,kw. (1)

6 (harmonic* adj (ACE* or Focus* or Synergy* or Wave*)).tw,kw. (501)

7 "ACE+ 7".tw,kw. (12)

8 "Harmonic 7".tw,kw. (2)

9 "CS 14-C".tw,kw. (1)

10 "HD 1000i Shears".tw,kw. (0)

11 HF005.tw,kw. (1)

12 Ultrasonic Surgical Procedures/ (336)

13 ((ultrason* or ultrasound*) adj2 surg* adj3 (device* or instrument* or procedur* or technique*)).tw,kw. (156)

14 or/1-13 (3072)

15 exp Animals/ not (exp Animals/ and Humans/) (4467392)

16 14 not 15 (2772)

17 (comment or editorial or interview or news or newspaper article).pt. (1261767)

18 (letter not (letter and randomized controlled trial)).pt. (984089)

19 16 not (17 or 18) (2699)

20 limit 19 to systematic reviews (57)

21 meta analysis.pt. (89435)

22 exp meta-analysis as topic/ (16644)

23 (meta-analy* or metanaly* or metaanaly* or met analy* or integrative research or integrative review* or integrative overview* or research integration or research overview* or collaborative review*).tw,kw. (133550)

24 (systematic review* or systematic overview* or evidence-based review* or evidence-based overview* or (evidence adj3 (review* or overview*)) or meta-review* or meta-overview* or meta-synthes* or rapid review* or "review of reviews" or technology assessment* or HTA or HTAs).tw,kw. (164954)

25 exp Technology assessment, biomedical/ (10370)

26 (cochrane or health technology assessment or evidence report).jw. (15579)

27 ((indirect* or mixed or multi-treatment*) adj2 compar*).tw,kw. (4167)

28 ((network* or network-based) adj (MA or MAs)).kw,tw. (5)

29 or/21-28 (283759)

30 19 and 29 (52)

31 20 or 30 [SYSTEMATIC REVIEWS] (66)

32 (controlled clinical trial or randomized controlled trial).pt. (550633)

33 clinical trials as topic.sh. (183938)

34 exp Randomized Controlled Trials as Topic/ (118960)

35 (randomi#ed or randomly or RCT$1 or placebo*).tw,kw. (848099)

36 ((singl* or doubl* or trebl* or tripl*) adj (mask* or blind* or dumm*)).tw,kw. (157384)

37 trial.ti. (183664)

38 or/32-37 (1252197)

39 19 and 38 [RCTs] (367)

40 controlled clinical trial.pt. (92463)

41 Controlled Clinical Trial/ or Controlled Clinical Trials as Topic/ (97761)

42 (control* adj2 trial*).tw,kw. (223352)

43 Non-Randomized Controlled Trials as Topic/ (363)

44 (nonrandom* or non-random* or quasi-random* or quasi-experiment*).tw,kw. (47879)

45 (nRCT or nRCTs or non-RCT$1).tw,kw. (659)

46 Controlled Before-After Studies/ (329)

47 (control* adj3 ("before and after" or "before after")).tw,kw. (3771)

48 Interrupted Time Series Analysis/ (439)

49 (time series adj3 interrupt*).tw,kw. (2145)

50 (pre- adj3 post-).tw,kw. (67166)

51 (pretest adj3 posttest).tw,kw. (4539)

52 Historically Controlled Study/ (142)

53 (control* adj2 stud$3).tw,kw. (204951)

54 Control Groups/ (1589)

55 (control* adj2 group$1).tw,kw. (435640)

56 trial.ti. (183664)

57 or/40-56 (1057195)

58 19 and 57 [NON-RCTs] (239)

59 exp Cohort Studies/ (1750857)

60 cohort$1.tw,kw. (462871)

61 Retrospective Studies/ (690404)

62 (longitudinal or prospective or retrospective).tw,kw. (1064237)

63 ((followup or follow-up) adj (study or studies)).tw,kw. (45896)

64 Observational study.pt. (48558)

65 (observation$2 adj (study or studies)).tw,kw. (83058)

66 ((population or population-based) adj (study or studies or analys#s)).tw,kw. (21804)

67 ((multidimensional or multi-dimensional) adj (study or studies)).tw,kw. (103)

68 Comparative Study.pt. (1800484)

69 ((comparative or comparison) adj (study or studies)).tw,kw. (99200)

70 exp Case-Control Studies/ (921302)

71 ((case-control* or case-based or case-comparison) adj (study or studies)).tw,kw. (92367)

72 or/59-71 (4044600)

73 19 and 72 [OBSERVATIONAL STUDIES] (1026)

74 31 or 39 or 58 or 73 [ALL STUDY DESIGNS] (1158)

75 (2016 10* or 2016 11* or 2016 12* or 2017* or 2018*).dt. (2082398)

76 74 and 75 [UPDATE] (118)

***************************

Embase

Database: Embase <1988 to 2018 Week 23>

Search Strategy:

--------------------------------------------------------------------------------

1 harmonic.dv. (698)

2 (harmonic* adj2 (blade? or dissect* or hook? or incis* or scalpel? or shear? or scalpel?)).tw,kw. (1702)

3 (ultrason* adj2 (blade? or dissect* or hook? or incis* or scalpel? or shear? or scalpel?)).tw,kw. (1457)

4 (ultrasound* adj2 (blade? or dissect* or hook? or incis* or scalpel? or shear? or scalpel?)).tw,kw. (666)

5 ultracision?.tw,kw. (262)

6 ultra-cision?.tw,kw. (5)

7 (harmonic* adj (ACE* or Focus* or Synergy* or Wave*)).tw,kw. (426)

8 "ACE+ 7".tw,kw. (17)

9 "Harmonic 7".tw,kw. (1)

10 "CS 14-C".tw,kw. (0)

11 "HD 1000i Shears".tw,kw. (0)

12 HF005.tw,kw. (0)

13 ultrasound surgery/ (438)

14 ((ultrason* or ultrasound*) adj2 surg* adj3 (device* or instrument* or procedur* or technique*)).tw,kw. (181)

15 or/1-14 (4845)

16 exp animal experimentation/ or exp models animal/ or exp animal experiment/ or nonhuman/ or exp vertebrate/ (21024156)

17 exp human/ or exp human experimentation/ or exp human experiment/ (16637849)

18 16 not 17 (4386851)

19 15 not 18 (4506)

20 editorial.pt. (537963)

21 letter.pt. not (letter.pt. and randomized controlled trial/) (890504)

22 19 not (20 or 21) (4416)

23 meta-analysis/ (144922)

24 "systematic review"/ (169493)

25 "meta analysis (topic)"/ (37802)

26 (meta-analy* or metanaly* or metaanaly* or met analy* or integrative research or integrative review* or integrative overview* or research integration or research overview* or collaborative review*).tw,kw. (174698)

27 (systematic review* or systematic overview* or evidence-based review* or evidence-based overview* or (evidence adj3 (review* or overview*)) or meta-review* or meta-overview* or meta-synthes* or rapid review* or "review of reviews" or technology assessment* or HTA or HTAs).tw,kw. (199126)

28 biomedical technology assessment/ (12145)

29 (cochrane or health technology assessment or evidence report).jw. (23475)

30 ((indirect* or mixed or multi-treatment*) adj2 compar*).tw,kw. (6295)

31 ((network* or network-based) adj (MA or MAs)).kw,tw. (12)

32 or/23-31 (421024)

33 22 and 32 [SYSTEMATIC REVIEWS] (111)

34 randomized controlled trial/ or controlled clinical trial/ (669537)

35 exp "clinical trial (topic)"/ (268812)

36 (randomi#ed or randomly or RCT$1 or placebo*).tw,kw. (1124252)

37 ((singl* or doubl* or trebl* or tripl*) adj (mask* or blind* or dumm*)).tw,kw. (188604)

38 trial.ti. (229380)

39 or/34-38 (1609111)

40 22 and 39 [RCTs] (582)

41 exp controlled clinical trial/ (669537)

42 exp "controlled clinical trial (topic)"/ (152286)

43 (control* adj2 trial*).tw,kw. (292305)

44 (nonrandom* or non-random* or quasi-random* or quasi-experiment*).tw,kw. (57154)

45 (nRCT or nRCTs or non-RCT$1).tw,kw. (951)

46 (control* adj3 ("before and after" or "before after")).tw,kw. (4572)

47 time series analysis/ (20825)

48 (time series adj3 interrupt*).tw,kw. (2632)

49 (pre- adj3 post-).tw,kw. (113709)

50 (pretest adj3 posttest).tw,kw. (4838)

51 controlled study/ (5922766)

52 (control* adj2 stud$3).tw,kw. (257956)

53 control group/ (110613)

54 (control* adj2 group$1).tw,kw. (581162)

55 trial.ti. (229380)

56 or/41-55 (6667644)

57 22 and 56 [NON-RCTs] (1104)

58 cohort analysis/ (375608)

59 cohort$1.tw,kw. (762061)

60 retrospective study/ (644408)

61 longitudinal study/ (111100)

62 prospective study/ (445722)

63 (longitudinal or prospective or retrospective).tw,kw. (1537040)

64 follow up/ (1256424)

65 ((followup or follow-up) adj (study or studies)).tw,kw. (53029)

66 observational study/ (140834)

67 (observation$2 adj (study or studies)).tw,kw. (128730)

68 population research/ (90562)

69 ((population or population-based) adj (study or studies or analys#s)).tw,kw. (20133)

70 ((multidimensional or multi-dimensional) adj (study or studies)).tw,kw. (109)

71 exp comparative study/ (1009667)

72 ((comparative or comparison) adj (study or studies)).tw,kw. (97010)

73 exp case control study/ (143835)

74 ((case-control* or case-based or case-comparison) adj (study or studies)).tw,kw. (115985)

75 or/58-74 (4245719)

76 22 and 75 [OBSERVATIONAL STUDIES] (1767)

77 33 or 40 or 57 or 76 [ALL STUDY DESIGNS] (2223)

78 ("20160924" or "20160925" or "20160926" or "20160927" or "20160928" or "20160929" or "20160930" or 201610* or 201611* or 201612* or 2017* or 2018*).dc. (2948783)

79 77 and 78 (427)

***************************

Cochrane Library

Search Name: Harmonic Scalpel

Date Run: 02/06/18 16:12:49.43

Description: Cornerstone - 2016 Oct 1

ID Search Hits

#1 (harmonic* near/2 (blade* or dissect* or hook* or incis* or scalpel* or shear* or scalpel*)):ti,ab,kw 275

#2 (ultrason* near/2 (blade* or dissect* or hook* or incis* or scalpel* or shear* or scalpel*)):ti,ab,kw 202

#3 (ultrasound* near/2 (blade* or dissect* or hook* or incis* or scalpel* or shear* or scalpel*)):ti,ab,kw 80

#4 ultracision*:ti,ab,kw 45

#5 ultra-cision*:ti,ab,kw 1

#6 (harmonic* next (ACE* or Focus* or Synergy* or Wave*)):ti,ab,kw 62

#7 "ACE+ 7":ti,ab,kw 1

#8 "Harmonic 7":ti,ab,kw 0

#9 "CS 14-C":ti,ab,kw 0

#10 "HD 1000i Shears":ti,ab,kw 0

#11 HF005:ti,ab,kw 0

#12 [mh ^"Ultrasonic Surgical Procedures"] 53

#13 (((ultrason* or ultrasound*) near/2 surg*) near/3 (device* or instrument* or procedur* or technique*)):ti,ab,kw 177

#14 {or #1-#13} Publication Year from 2016 to 2018 151

DSR – 5

CENTRAL - 146

- 1. Search Strategy for October 1, 2016, search

Harmonic Scalpel

Final Strategies

2016 Oct 1

OVID Multifile

Database: Embase <1988 to 2016 Week 39>, Epub Ahead of Print, In-Process & Other Non-Indexed Citations, Ovid MEDLINE(R) Daily and Ovid MEDLINE(R) <1946 to Present>

Search Strategy:

--------------------------------------------------------------------------------

1 (harmonic* adj2 (blade? or dissect* or hook? or incis* or scalpel? or shear? or scalpel?)).tw,kw. (2454)

2 (ultrasonic* adj2 (blade? or dissect* or hook? or incis* or scalpel? or shear? or scalpel?)).tw,kw. (2161)

3 (ultrasound* adj2 (blade? or dissect* or hook? or incis* or scalpel? or shear? or scalpel?)).tw,kw. (838)

4 ultracision?.tw,kw. (376)

5 ultra-cision?.tw,kw. (5)

6 (harmonic* adj (ACE* or Focus* or Synergy* or Wave*)).tw,kw. (812)

7 "ACE+ 7".tw,kw. (21)

8 "Harmonic 7".tw,kw. (3)

9 "CS 14-C".tw,kw. (1)

10 "HD 1000i Shears".tw,kw. (0)

11 HF005.tw,kw. (1)

12 Ultrasonic Surgical Procedures/ (654)

13 ((ultrason* or ultrasound*) adj2 surg* adj3 (device* or instrument* or procedur* or technique*)).tw,kw. (306)

14 or/1-13 (6556)

15 exp Animals/ not (exp Animals/ and Humans/) (13727370)

16 14 not 15 (4336)

17 (comment or editorial or interview or news or newspaper article).pt. (1671785)

18 (letter not (letter and randomized controlled trial)).pt. (1762745)

19 16 not (17 or 18) (4233)

20 limit 19 to systematic reviews [Limit not valid in Embase; records were retained] (1899)

21 meta analysis.pt. (73990)

22 exp meta-analysis as topic/ (49955)

23 (meta-analy* or metanaly* or metaanaly* or met analy* or integrative research or integrative review* or integrative overview* or research integration or research overview* or collaborative review*).tw,kw. (242010)

24 (systematic review* or systematic overview* or evidence-based review* or evidence-based overview* or (evidence adj3 (review* or overview*)) or meta-review* or meta-overview* or meta-synthes* or rapid review* or "review of reviews" or technology assessment* or HTA or HTAs).tw,kw. (282350)

25 exp Technology assessment, biomedical/ (20902)

26 (cochrane or health technology assessment or evidence report).jw. (34521)

27 ((indirect* or mixed or multi-treatment*) adj2 compar*).tw,kw. (8665)

28 ((network* or network-based) adj (MA or MAs)).kw,tw. (11)

29 or/21-28 (519420)

30 19 and 29 (65)

31 20 or 30 [SYSTEMATIC REVIEWS] (1908)

32 (controlled clinical trial or randomized controlled trial).pt. (519165)

33 clinical trials as topic.sh. (179761)

34 exp Randomized Controlled Trials as Topic/ (231226)

35 (randomi#ed or randomly or RCT$1 or placebo*).tw,kw. (1740069)

36 ((singl* or doubl* or trebl* or tripl*) adj (mask* or blind* or dumm*)).tw,kw. (316622)

37 trial.ti. (354659)

38 or/32-37 (2254804)

39 19 and 38 [RCTs] (498)

40 controlled clinical trial.pt. (91760)

41 Controlled Clinical Trial/ or Controlled Clinical Trials as Topic/ (540950)

42 (control* adj2 trial*).tw,kw. (436814)

43 Non-Randomized Controlled Trials as Topic/ (10101)

44 (nonrandom* or non-random* or quasi-random* or quasi-experiment*).tw,kw. (91083)

45 (nRCT or nRCTs or non-RCT$1).tw,kw. (1182)

46 Controlled Before-After Studies/ (167992)

47 (control* adj3 ("before and after" or "before after")).tw,kw. (7222)

48 Interrupted Time Series Analysis/ (152036)

49 (time series adj3 interrupt*).tw,kw. (3539)

50 (pre- adj3 post-).tw,kw. (147710)

51 (pretest adj3 posttest).tw,kw. (7838)

52 Historically Controlled Study/ (187204)

53 (control* adj2 stud$3).tw,kw. (415123)

54 Control Groups/ (255317)

55 (control* adj2 group$1).tw,kw. (895570)

56 trial.ti. (354659)

57 or/40-56 (2531267)

58 19 and 57 [NON-RCTs] (397)

59 exp Cohort Studies/ (1888851)

60 cohort$1.tw,kw. (988452)

61 Retrospective Studies/ (841566)

62 (longitudinal or prospective or retrospective).tw,kw. (2212090)

63 ((followup or follow-up) adj (study or studies)).tw,kw. (91014)

64 Observational study.pt. (26695)

65 (observation$2 adj (study or studies)).tw,kw. (167950)

66 ((population or population-based) adj (study or studies or analys#s)).tw,kw. (38289)

67 ((multidimensional or multi-dimensional) adj (study or studies)).tw,kw. (184)

68 Comparative Study.pt. (1770466)

69 ((comparative or comparison) adj (study or studies)).tw,kw. (178703)

70 exp Case-Control Studies/ (952195)

71 ((case-control* or case-based or case-comparison) adj (study or studies)).tw,kw. (185277)

72 or/59-71 (5817869)

73 19 and 72 [OBSERVATIONAL STUDIES] (1301)

74 31 or 39 or 58 or 73 [ALL STUDY DESIGNS] (2894)

75 74 use ppez (1044) [MEDLINE RECORDS]

76 harmonic.dv. (603)

77 (harmonic* adj2 (blade? or dissect* or hook? or incis* or scalpel? or shear? or scalpel?)).tw,kw. (2454)

78 (ultrason* adj2 (blade? or dissect* or hook? or incis* or scalpel? or shear? or scalpel?)).tw,kw. (2300)

79 (ultrasound* adj2 (blade? or dissect* or hook? or incis* or scalpel? or shear? or scalpel?)).tw,kw. (838)

80 ultracision?.tw,kw. (376)

81 ultra-cision?.tw,kw. (5)

82 (harmonic* adj (ACE* or Focus* or Synergy* or Wave*)).tw,kw. (812)

83 "ACE+ 7".tw,kw. (21)

84 "Harmonic 7".tw,kw. (3)

85 "CS 14-C".tw,kw. (1)

86 "HD 1000i Shears".tw,kw. (0)

87 HF005.tw,kw. (1)

88 ultrasound surgery/ (387)

89 ((ultrason* or ultrasound*) adj2 surg* adj3 (device* or instrument* or procedur* or technique*)).tw,kw. (306)

90 or/76-89 (6828)

91 exp animal experimentation/ or exp models animal/ or exp animal experiment/ or nonhuman/ or exp vertebrate/ (39067187)

92 exp human/ or exp human experimentation/ or exp human experiment/ (31426764)

93 91 not 92 (7641574)

94 90 not 93 (6291)

95 editorial.pt. (906336)

96 letter.pt. not (letter.pt. and randomized controlled trial/) (1757763)

97 94 not (95 or 96) (6158)

98 meta-analysis/ (221672)

99 "systematic review"/ (139042)

100 "meta analysis (topic)"/ (34527)

101 (meta-analy* or metanaly* or metaanaly* or met analy* or integrative research or integrative review* or integrative overview* or research integration or research overview* or collaborative review*).tw,kw. (242010)

102 (systematic review* or systematic overview* or evidence-based review* or evidence-based overview* or (evidence adj3 (review* or overview*)) or meta-review* or meta-overview* or meta-synthes* or rapid review* or "review of reviews" or technology assessment* or HTA or HTAs).tw,kw. (282350)

103 biomedical technology assessment/ (19793)

104 (cochrane or health technology assessment or evidence report).jw. (34521)

105 ((indirect* or mixed or multi-treatment*) adj2 compar*).tw,kw. (8665)

106 ((network* or network-based) adj (MA or MAs)).kw,tw. (11)

107 or/98-106 (561546)

108 97 and 107 [SYSTEMATIC REVIEWS] (132)

109 randomized controlled trial/ or controlled clinical trial/ (1109541)

110 exp "clinical trial (topic)"/ (256945)

111 (randomi#ed or randomly or RCT$1 or placebo*).tw,kw. (1740069)

112 ((singl* or doubl* or trebl* or tripl*) adj (mask* or blind* or dumm*)).tw,kw. (316622)

113 trial.ti. (354659)

114 or/109-113 (2404037)

115 97 and 114 [RCTs] (821)

116 exp controlled clinical trial/ (1109665)

117 exp "controlled clinical trial (topic)"/ (125448)

118 (control* adj2 trial*).tw,kw. (436814)

119 (nonrandom* or non-random* or quasi-random* or quasi-experiment*).tw,kw. (91083)

120 (nRCT or nRCTs or non-RCT$1).tw,kw. (1182)

121 (control* adj3 ("before and after" or "before after")).tw,kw. (7222)

122 time series analysis/ (23164)

123 (time series adj3 interrupt*).tw,kw. (3539)

124 (pre- adj3 post-).tw,kw. (147710)

125 (pretest adj3 posttest).tw,kw. (7838)

126 controlled study/ (5135971)

127 (control* adj2 stud$3).tw,kw. (415123)

128 control group/ (255317)

129 (control* adj2 group$1).tw,kw. (895570)

130 trial.ti. (354659)

131 or/116-130 (6991487)

132 97 and 131 [NON-RCTs] (1248)

133 cohort analysis/ (495917)

134 cohort$1.tw,kw. (988452)

135 retrospective study/ (1095490)

136 longitudinal study/ (205538)

137 prospective study/ (803888)

138 (longitudinal or prospective or retrospective).tw,kw. (2212090)

139 follow up/ (1160020)

140 ((followup or follow-up) adj (study or studies)).tw,kw. (91014)

141 observational study/ (145370)

142 (observation$2 adj (study or studies)).tw,kw. (167950)

143 population research/ (87063)

144 ((population or population-based) adj (study or studies or analys#s)).tw,kw. (38289)

145 ((multidimensional or multi-dimensional) adj (study or studies)).tw,kw. (184)

146 exp comparative study/ (2681728)

147 ((comparative or comparison) adj (study or studies)).tw,kw. (178703)

148 exp case control study/ (952195)

149 ((case-control* or case-based or case-comparison) adj (study or studies)).tw,kw. (185277)

150 or/133-149 (7156712)

151 97 and 150 [OBSERVATIONAL STUDIES] (2311)

152 108 or 115 or 132 or 151 [ALL STUDY DESIGNS] (2829)

153 152 use emed (1897) [EMBASE RECORDS]

154 75 or 153 [BOTH DATABASES] (2941)

155 remove duplicates from 154 (2081) [TOTAL UNIQUE RECORDS]

156 155 use ppez [MEDLINE UNIQUE RECORDS] (1016)

157 155 use emed [EMBASE UNIQUE RECORDS] (1065)

***************************

Cochrane Library

Search Name: Harmonic Scalpel

Date Run: 01/10/16 12:00:11.30

Description: Cornerstone - 2016 Oct 1

ID Search Hits

#1 (harmonic* near/2 (blade* or dissect* or hook* or incis* or scalpel* or shear* or scalpel*)):ti,ab,kw 213

#2 (ultrason* near/2 (blade* or dissect* or hook* or incis* or scalpel* or shear* or scalpel*)):ti,ab,kw 157

#3 (ultrasound* near/2 (blade* or dissect* or hook* or incis* or scalpel* or shear* or scalpel*)):ti,ab,kw 54

#4 ultracision*:ti,ab,kw 38

#5 ultra-cision*:ti,ab,kw 1

#6 (harmonic* next (ACE* or Focus* or Synergy* or Wave*)):ti,ab,kw 44

#7 "ACE+ 7":ti,ab,kw 1

#8 "Harmonic 7":ti,ab,kw 0

#9 "CS 14-C":ti,ab,kw 0

#10 "HD 1000i Shears":ti,ab,kw 0

#11 HF005:ti,ab,kw 0

#12 [mh ^"Ultrasonic Surgical Procedures"] 36

#13 (((ultrason* or ultrasound*) near/2 surg*) near/3 (device* or instrument* or procedur* or technique*)):ti,ab,kw 107

#14 {or #1-#13} 458

DSR – 6

DARE – 11

CENTRAL – 417

HTA – 4

NHS EED – 20 [*did not download*]

ANIMAL STUDIES

OVID Multifile

Database: Embase <1988 to 2016 Week 39>, Epub Ahead of Print, In-Process & Other Non-Indexed Citations, Ovid MEDLINE(R) Daily and Ovid MEDLINE(R) <1946 to Present>

Search Strategy:

--------------------------------------------------------------------------------

1 (harmonic* adj2 (blade? or dissect* or hook? or incis* or scalpel? or shear? or scalpel?)).tw,kw. (2454)

2 (ultrasonic* adj2 (blade? or dissect* or hook? or incis* or scalpel? or shear? or scalpel?)).tw,kw. (2161)

3 (ultrasound* adj2 (blade? or dissect* or hook? or incis* or scalpel? or shear? or scalpel?)).tw,kw. (838)

4 ultracision?.tw,kw. (376)

5 ultra-cision?.tw,kw. (5)

6 (harmonic* adj (ACE* or Focus* or Synergy* or Wave*)).tw,kw. (812)

7 "ACE+ 7".tw,kw. (21)

8 "Harmonic 7".tw,kw. (3)

9 "CS 14-C".tw,kw. (1)

10 "HD 1000i Shears".tw,kw. (0)

11 HF005.tw,kw. (1)

12 Ultrasonic Surgical Procedures/ (654)

13 ((ultrason* or ultrasound*) adj2 surg* adj3 (device* or instrument* or procedur* or technique*)).tw,kw. (306)

14 or/1-13 (6556)

15 exp Animals/ not (exp Animals/ and Humans/) (13727370)

16 14 not 15 (4336)

17 (comment or editorial or interview or news or newspaper article).pt. (1671785)

18 (letter not (letter and randomized controlled trial)).pt. (1762745)

19 16 not (17 or 18) (4233)

20 limit 19 to systematic reviews [Limit not valid in Embase; records were retained] (1899)

21 meta analysis.pt. (73990)

22 exp meta-analysis as topic/ (49955)

23 (meta-analy* or metanaly* or metaanaly* or met analy* or integrative research or integrative review* or integrative overview* or research integration or research overview* or collaborative review*).tw,kw. (242010)

24 (systematic review* or systematic overview* or evidence-based review* or evidence-based overview* or (evidence adj3 (review* or overview*)) or meta-review* or meta-overview* or meta-synthes* or rapid review* or "review of reviews" or technology assessment* or HTA or HTAs).tw,kw. (282350)

25 exp Technology assessment, biomedical/ (20902)

26 (cochrane or health technology assessment or evidence report).jw. (34521)

27 ((indirect* or mixed or multi-treatment*) adj2 compar*).tw,kw. (8665)

28 ((network* or network-based) adj (MA or MAs)).kw,tw. (11)

29 or/21-28 (519420)

30 19 and 29 (65)

31 20 or 30 [SYSTEMATIC REVIEWS] (1908)

32 (controlled clinical trial or randomized controlled trial).pt. (519165)

33 clinical trials as topic.sh. (179761)

34 exp Randomized Controlled Trials as Topic/ (231226)

35 (randomi#ed or randomly or RCT$1 or placebo*).tw,kw. (1740069)

36 ((singl* or doubl* or trebl* or tripl*) adj (mask* or blind* or dumm*)).tw,kw. (316622)

37 trial.ti. (354659)

38 or/32-37 (2254804)

39 19 and 38 [RCTs] (498)

40 controlled clinical trial.pt. (91760)

41 Controlled Clinical Trial/ or Controlled Clinical Trials as Topic/ (540950)

42 (control* adj2 trial*).tw,kw. (436814)

43 Non-Randomized Controlled Trials as Topic/ (10101)

44 (nonrandom* or non-random* or quasi-random* or quasi-experiment*).tw,kw. (91083)

45 (nRCT or nRCTs or non-RCT$1).tw,kw. (1182)

46 Controlled Before-After Studies/ (167992)

47 (control* adj3 ("before and after" or "before after")).tw,kw. (7222)

48 Interrupted Time Series Analysis/ (152036)

49 (time series adj3 interrupt*).tw,kw. (3539)

50 (pre- adj3 post-).tw,kw. (147710)

51 (pretest adj3 posttest).tw,kw. (7838)

52 Historically Controlled Study/ (187204)

53 (control* adj2 stud$3).tw,kw. (415123)

54 Control Groups/ (255317)

55 (control* adj2 group$1).tw,kw. (895570)

56 trial.ti. (354659)

57 or/40-56 (2531267)

58 19 and 57 [NON-RCTs] (397)

59 exp Cohort Studies/ (1888851)

60 cohort$1.tw,kw. (988452)

61 Retrospective Studies/ (841566)

62 (longitudinal or prospective or retrospective).tw,kw. (2212090)

63 ((followup or follow-up) adj (study or studies)).tw,kw. (91014)

64 Observational study.pt. (26695)

65 (observation$2 adj (study or studies)).tw,kw. (167950)

66 ((population or population-based) adj (study or studies or analys#s)).tw,kw. (38289)

67 ((multidimensional or multi-dimensional) adj (study or studies)).tw,kw. (184)

68 Comparative Study.pt. (1770466)

69 ((comparative or comparison) adj (study or studies)).tw,kw. (178703)

70 exp Case-Control Studies/ (952195)

71 ((case-control* or case-based or case-comparison) adj (study or studies)).tw,kw. (185277)

72 or/59-71 (5817869)

73 19 and 72 [OBSERVATIONAL STUDIES] (1301)

74 31 or 39 or 58 or 73 [ALL STUDY DESIGNS] (2894)

75 14 not (17 or 18) (6413)

76 limit 75 to systematic reviews [Limit not valid in Embase; records were retained] (3804)

77 75 and 29 (120)

78 76 or 77 (3813)

79 75 and 38 (775)

80 75 and 57 (625)

81 75 and 72 (1834)

82 or/78-81 (4926)

83 82 not 74 [ANIMAL STUDIES] (2032)

84 83 use ppez (127) [MEDLINE RECORDS]

85 harmonic.dv. (603)

86 (harmonic* adj2 (blade? or dissect* or hook? or incis* or scalpel? or shear? or scalpel?)).tw,kw. (2454)

87 (ultrason* adj2 (blade? or dissect* or hook? or incis* or scalpel? or shear? or scalpel?)).tw,kw. (2300)

88 (ultrasound* adj2 (blade? or dissect* or hook? or incis* or scalpel? or shear? or scalpel?)).tw,kw. (838)

89 ultracision?.tw,kw. (376)

90 ultra-cision?.tw,kw. (5)

91 (harmonic* adj (ACE* or Focus* or Synergy* or Wave*)).tw,kw. (812)

92 "ACE+ 7".tw,kw. (21)

93 "Harmonic 7".tw,kw. (3)

94 "CS 14-C".tw,kw. (1)

95 "HD 1000i Shears".tw,kw. (0)

96 HF005.tw,kw. (1)

97 ultrasound surgery/ (387)

98 ((ultrason* or ultrasound*) adj2 surg* adj3 (device* or instrument* or procedur* or technique*)).tw,kw. (306)

99 or/85-98 (6828)

100 exp animal experimentation/ or exp models animal/ or exp animal experiment/ or nonhuman/ or exp vertebrate/ (39067187)

101 exp human/ or exp human experimentation/ or exp human experiment/ (31426764)

102 100 not 101 (7641574)

103 99 not 102 (6291)

104 editorial.pt. (906336)

105 letter.pt. not (letter.pt. and randomized controlled trial/) (1757763)

106 103 not (104 or 105) (6158)

107 meta-analysis/ (221672)

108 "systematic review"/ (139042)

109 "meta analysis (topic)"/ (34527)

110 (meta-analy* or metanaly* or metaanaly* or met analy* or integrative research or integrative review* or integrative overview* or research integration or research overview* or collaborative review*).tw,kw. (242010)

111 (systematic review* or systematic overview* or evidence-based review* or evidence-based overview* or (evidence adj3 (review* or overview*)) or meta-review* or meta-overview* or meta-synthes* or rapid review* or "review of reviews" or technology assessment* or HTA or HTAs).tw,kw. (282350)

112 biomedical technology assessment/ (19793)

113 (cochrane or health technology assessment or evidence report).jw. (34521)

114 ((indirect* or mixed or multi-treatment*) adj2 compar*).tw,kw. (8665)

115 ((network* or network-based) adj (MA or MAs)).kw,tw. (11)

116 or/107-115 (561546)

117 106 and 116 [SYSTEMATIC REVIEWS] (132)

118 randomized controlled trial/ or controlled clinical trial/ (1109541)

119 exp "clinical trial (topic)"/ (256945)

120 (randomi#ed or randomly or RCT$1 or placebo*).tw,kw. (1740069)

121 ((singl* or doubl* or trebl* or tripl*) adj (mask* or blind* or dumm*)).tw,kw. (316622)

122 trial.ti. (354659)

123 or/118-122 (2404037)

124 106 and 123 [RCTs] (821)

125 exp controlled clinical trial/ (1109665)

126 exp "controlled clinical trial (topic)"/ (125448)

127 (control* adj2 trial*).tw,kw. (436814)

128 (nonrandom* or non-random* or quasi-random* or quasi-experiment*).tw,kw. (91083)

129 (nRCT or nRCTs or non-RCT$1).tw,kw. (1182)

130 (control* adj3 ("before and after" or "before after")).tw,kw. (7222)

131 time series analysis/ (23164)

132 (time series adj3 interrupt*).tw,kw. (3539)

133 (pre- adj3 post-).tw,kw. (147710)

134 (pretest adj3 posttest).tw,kw. (7838)

135 controlled study/ (5135971)

136 (control* adj2 stud$3).tw,kw. (415123)

137 control group/ (255317)

138 (control* adj2 group$1).tw,kw. (895570)

139 trial.ti. (354659)

140 or/125-139 (6991487)

141 106 and 140 [NON-RCTs] (1248)

142 cohort analysis/ (495917)

143 cohort$1.tw,kw. (988452)

144 retrospective study/ (1095490)

145 longitudinal study/ (205538)

146 prospective study/ (803888)

147 (longitudinal or prospective or retrospective).tw,kw. (2212090)

148 follow up/ (1160020)

149 ((followup or follow-up) adj (study or studies)).tw,kw. (91014)

150 observational study/ (145370)

151 (observation$2 adj (study or studies)).tw,kw. (167950)

152 population research/ (87063)

153 ((population or population-based) adj (study or studies or analys#s)).tw,kw. (38289)

154 ((multidimensional or multi-dimensional) adj (study or studies)).tw,kw. (184)

155 exp comparative study/ (2681728)

156 ((comparative or comparison) adj (study or studies)).tw,kw. (178703)

157 exp case control study/ (952195)

158 ((case-control* or case-based or case-comparison) adj (study or studies)).tw,kw. (185277)

159 or/142-158 (7156712)

160 106 and 159 [OBSERVATIONAL STUDIES] (2311)

161 117 or 124 or 141 or 160 [ALL STUDY DESIGNS] (2829)

162 99 not (104 or 105) (6695)

163 162 and 116 (132)

164 162 and 123 (866)

165 162 and 140 (1390)

166 162 and 159 (2515)

167 or/163-166 (3130)

168 167 not 161 [ANIMAL STUDIES] (301)

169 168 use emed [EMBASE RECORDS] (176)

170 84 or 169 [BOTH DATABASES] (303)

171 remove duplicates from 170 (213) [TOTAL UNIQUE RECORDS]

172 171 use ppez [MEDLINE UNIQUE RECORDS] (123)

173 171 use emed [EMBASE UNIQUE RECORDS] (90)

***************************

1. Supplementary Tables

Supplementary Table 1: PRISMA Checklist

| **Section/topic** | **#** | **Checklist item** | **Reported on page #** |
| --- | --- | --- | --- |
| **TITLE** | | |  |
| Title | 1 | Identify the report as a systematic review, meta-analysis, or both. | Page 1 |
| **ABSTRACT** | | |  |
| Structured summary | 2 | Provide a structured summary including, as applicable: background; objectives; data sources; study eligibility criteria, participants, and interventions; study appraisal and synthesis methods; results; limitations; conclusions and implications of key findings; systematic review registration number. | Pages 2 |
| **INTRODUCTION** | | |  |
| Rationale | 3 | Describe the rationale for the review in the context of what is already known. | Page 4 |
| Objectives | 4 | Provide an explicit statement of questions being addressed with reference to participants, interventions, comparisons, outcomes, and study design (PICOS). | Pages 5-6 |
| **METHODS** | | |  |
| Protocol and registration | 5 | Indicate if a review protocol exists, if and where it can be accessed (e.g., Web address), and, if available, provide registration information including registration number. | NA |
| Eligibility criteria | 6 | Specify study characteristics (e.g., PICOS, length of follow-up) and report characteristics (e.g., years considered, language, publication status) used as criteria for eligibility, giving rationale. | Pages 5-6 |
| Information sources | 7 | Describe all information sources (e.g., databases with dates of coverage, contact with study authors to identify additional studies) in the search and date last searched. | Page 4 |
| Search | 8 | Present full electronic search strategy for at least one database, including any limits used, such that it could be repeated. | Appendix A |
| Study selection | 9 | State the process for selecting studies (i.e., screening, eligibility, included in systematic review, and, if applicable, included in the meta-analysis). | Pages 5-6 |
| Data collection process | 10 | Describe method of data extraction from reports (e.g., piloted forms, independently, in duplicate) and any processes for obtaining and confirming data from investigators. | Pages 5-6 |
| Data items | 11 | List and define all variables for which data were sought (e.g., PICOS, funding sources) and any assumptions and simplifications made. | Page 5 |
| Risk of bias in individual studies | 12 | Describe methods used for assessing risk of bias of individual studies (including specification of whether this was done at the study or outcome level), and how this information is to be used in any data synthesis. | Page 6 |
| Summary measures | 13 | State the principal summary measures (e.g., risk ratio, difference in means). | Page 5 |
| Synthesis of results | 14 | Describe the methods of handling data and combining results of studies, if done, including measures of consistency (e.g., I^2^) for each meta-analysis. | NA |
| **Section/topic** | **#** | **Checklist item** | **Reported on page #** |
| Risk of bias across studies | 15 | Specify any assessment of risk of bias that may affect the cumulative evidence (e.g., publication bias, selective reporting within studies). | Page 6 |
| Additional analyses | 16 | Describe methods of additional analyses (e.g., sensitivity or subgroup analyses, meta-regression), if done, indicating which were pre-specified. | NA |
| **RESULTS** | | |  |
| Study selection | 17 | Give numbers of studies screened, assessed for eligibility, and included in the review, with reasons for exclusions at each stage, ideally with a flow diagram. | Figure 1 |
| Study characteristics | 18 | For each study, present characteristics for which data were extracted (e.g., study size, PICOS, follow-up period) and provide the citations. | Tables 1 and 2 Suppl. Tables 2, 3, 7 |
| Risk of bias within studies | 19 | Present data on risk of bias of each study and, if available, any outcome level assessment (see item 12). | Suppl Tables 4-6 |
| Results of individual studies | 20 | For all outcomes considered (benefits or harms), present, for each study: (a) simple summary data for each intervention group (b) effect estimates and confidence intervals, ideally with a forest plot. | Figure 2, Suppl table 7 |
| Synthesis of results | 21 | Present results of each meta-analysis done, including confidence intervals and measures of consistency. | NA |
| Risk of bias across studies | 22 | Present results of any assessment of risk of bias across studies (see Item 15). | Suppl tables 4-6 |
| Additional analysis | 23 | Give results of additional analyses, if done (e.g., sensitivity or subgroup analyses, meta-regression [see Item 16]). | NA |
| **DISCUSSION** | | |  |
| Summary of evidence | 24 | Summarize the main findings including the strength of evidence for each main outcome; consider their relevance to key groups (e.g., healthcare providers, users, and policy makers). | Pages 13-19 |
| Limitations | 25 | Discuss limitations at study and outcome level (e.g., risk of bias), and at review-level (e.g., incomplete retrieval of identified research, reporting bias). | Pages 18-19 |
| Conclusions | 26 | Provide a general interpretation of the results in the context of other evidence, and implications for future research. | Page 19 |
| **FUNDING** | | |  |
| Funding | 27 | Describe sources of funding for the systematic review and other support (e.g., supply of data); role of funders for the systematic review. | Page 20 |

*From:*  Moher D, Liberati A, Tetzlaff J, Altman DG, The PRISMA Group (2009). Preferred Reporting Items for Systematic Reviews and Meta-Analyses: The PRISMA Statement. PLoS Med 6(7): e1000097. doi:10.1371/journal.pmed1000097

For more information, visit: **www.prisma-statement.org**

Supplementary Table 2 Summary of additional RCTs

| **Study** | **Procedure** | | **Comparator Type** | | **Total Population Size (n)** | | **Operating Time (min)** | | | | | | **Length of Stay** | | | | | | **Interoperative blood loss (mL)** | | | | | | **Drainage Volume (mL)** | | | | | | **Pain (VAS)** | | | | | | **Overall Complications (% of patients)** | | | | | |
| --- | --- | --- | --- | --- | --- | --- | --- | --- | --- | --- | --- | --- | --- | --- | --- | --- | --- | --- | --- | --- | --- | --- | --- | --- | --- | --- | --- | --- | --- | --- | --- | --- | --- | --- | --- | --- | --- | --- | --- | --- | --- | --- |
|  |  |  |  |  |  |  | **HS** | | **CD** | | **p** | | **HS** | | **CD** | | **p** | | **HS** | | **CD** | | **p** | | **HS** | | **CD** | | **p** | | **HS** | | **CD** | | **p** | | **HS** | | **CD** | | **p** | |
| **Conventional Comparators** | | | | | | | | | | | | | | | | | | | | | | | | | | | | | | | | | | | | | | | | | | |
| Bessa 2011 (1) | | Cholecystectomy | | Conventional | | 40 | | **55†*** | | **82.5†** | | **0** | | 2† | | 2† | | 0.075 | | **50†*** | | **120†** | | **0** | | - | | - | | - | | - | | - | | - | | - | | - | | - |
| Liao 2016 (2) | | Laparoscopic cholecystectomy | | Conventional | | 198 | | 54.9 | | 51.7 | | 0.079 | | 3 | | 2.9 | | 0.315 | | 14.2 | | 13.7 | | 0.367 | | - | | - | | - | | - | | - | | - | | - | | - | | - |
| Mattila 2016 (3) | | Laparoscopic cholecystectomy | | Conventional | | 167 | | 45 | | 45 | | 0.95 | | - | | - | | - | | 13 | | 16 | | 0.45 | | - | | - | | - | | - | | - | | - | | 3.00% | | 5.00% | | 71.00% |
| Abdelhady 2017 (4) | | Cholecystectomy | | Conventional | | 60 | | **48.4*** | | **58.6** | | **0.032** | | **1.1*** | | **1.33** | | **0.046** | | - | | - | | - | | - | | - | | - | | - | | - | | - | | - | | - | | - |
| Sanawan 2017 (5) | | Cholecystectomy | | Conventional | | 150 | | **28.93*** | | **40.07** | | **<0.0001** | | 1.37 | | 1.62 | | 0.708 | | **5†*** | | **10†** | | **0.001** | | **15†*** | | **30†** | | **<0.0001** | | 3† | | 3† | | NR | | - | | - | | - |
| Shabbir 2017 (6) | | Cholecystectomy | | Conventional | | 120 | | 34.05 | | 31.97 | | NR | | - | | - | | - | | - | | - | | - | | - | | - | | - | | - | | - | | - | | - | | - | | - |
| Ahmed 2019 (7) | | Cholecystectomy | | Conventional | | 144 | | **28.74*** | | **43.71** | | **<0.001** | | 1.1 | | 1.19 | | >0.05 | | **3.13*** | | **7.14** | | **<0.001** | | - | | - | | - | | - | | - | | - | | - | | - | | - |
| Awale 2019 (8) | | Cholecystectomy | | Conventional | | 112 | | **35.91** | | **41.12** | | **0.054** | | 1† | | 1† | | 0.23 | | - | | - | | - | | - | | - | | - | | **3.91*** | | **5.31** | | **<0.001** | | 3.77% | | 8.47% | | 44.00% |
| Wilhelm 2011 (9) | | Open left hemicolectomy or total gastrectomy and LND | | Conventional | | 201 | | 170 | | 178 | | 0.405 | | 12† | | 12† | | 0.671 | | 350† | | 400† | | 0.882 | | - | | - | | - | | - | | - | | - | | 29.00% | | 28.70% | | 77.10% |
| Sista 2013 (10) | | Colectomy and LND | | Conventional | | 211 | | **103*** | | **131** | | **<0.05** | | 8 | | 11 | | NS | | - | | - | | - | | **125*** | | **155** | | **<0.05** | | 4 | | 4 | | NR | | **9%*** | | **23.00%** | | **<0.05** |
| Kawabata 2016 (11) | | Open gastrectomy and LND | | Conventional | | 237 | | 141 | | 147 | | 0.243 | | 18.6 | | 17.6 | | 0.986 | | 365 | | 336 | | 0.434 | | 172 | | 179 | | 0.583 | | - | | - | | - | | 26.80% | | 17.50% | | 8.50% |
| Oh 2017 (12) | | Open distal gastrectomy and LND | | Conventional | | 49 | | 167.7 | | 177.54 | | 0.244 | | 10.3 | | 8.5 | | 0.954 | | **339.8*** | | **428.6** | | **0.021** | | 774 | | 639.4 | | 0.303 | | - | | - | | - | | 28.00% | | 30.40% | | 85.30% |
| Kim 2018 (13) | | Gastrectomy | | Conventional | | 253 | | - | | - | | - | | - | | - | | - | | - | | - | | - | | - | | - | | - | | - | | - | | - | | 4.70% | | 4.00% | | 100.00% |
| Tsunoda 2011 (14) | | Hemorrhoidectomy | | Conventional | | 60 | | **31†** | | **16†*** | | **<0.0001** | | 2† | | 2† | | 0.275 | | **4.6†** | | **0.9†*** | | **0.001** | | - | | - | | - | | - | | - | | - | | - | | - | | - |
| Peker 2013 (15) | | Hemorrhoidectomy | | Conventional | | 46 | | **12.6*** | | **22.3** | | **<0.001** | | - | | - | | - | | - | | - | | - | | - | | - | | - | | - | | - | | - | | - | | - | | - |
| Bilgin 2014 (16) | | Hemorrhoidectomy | | Conventional | | 99 | | **17*** | | **22** | | **<0.05** | | 2.4 | | 2.6 | | NS | | - | | - | | - | | - | | - | | - | | **6** | | **1*** | | **<0.05** | | - | | - | | - |
| Bulus 2014 (17) | | Hemorrhoidectomy | | Conventional | | 151 | | **16.8*** | | **25.5** | | **0.001** | | - | | - | | - | | - | | - | | - | | - | | - | | - | | **5.4*** | | **6.8** | | **0.001** | | - | | - | | - |
| Shoukat 2016 (18) | | Hemorrhoidectomy | | Conventional | | 260 | | **2.77*** | | **3.89** | | **<0.001** | | - | | - | | - | | - | | - | | - | | - | | - | | - | | **2.77*** | | **3.89** | | **<0.0001** | | - | | - | | - |
| Ahmad 2021 (19) | | Hemorrhoidectomy | | Conventional | | 60 | | **20.8*** | | **26.5** | | **0.001** | | - | | - | | - | | - | | - | | - | | - | | - | | - | | - | | - | | - | | - | | - | | - |
| Nawaz 2015 (20) | | Modified radical mastectomy | | Conventional | | 80 | | - | | - | | - | | - | | - | | - | | - | | - | | - | | **167.75*** | | **310** | | **<0.001** | | - | | - | | - | | - | | - | | - |
| Mittal 2017 (21) | | Modified radical mastectomy | | Conventional | | 50 | | **140.4** | | **99.8*** | | **<0.001** | | 14.32 | | 19.04 | | 0.621 | | **426*** | | **502** | | **0.005** | | **431.6*** | | **594.2** | | **0.013** | | 5.08 | | 5.2 | | 0.778 | | - | | - | | - |
| Shanmugam 2017 (22) | | Modified radical mastectomy | | Conventional | | 100 | | **134.8** | | **101.8*** | | **<0.001** | | 14.1 | | 13.5 | | 0.68 | | 189.6 | | 211.4 | | 0.06 | | 1163.3 | | 1091.2 | | 0.84 | | 4.69 | | 4.97 | | 0.87 | | - | | - | | - |
| Archana 2018 (23) | | Modified radical mastectomy | | Conventional | | 240 | | **112.33*** | | **151.38** | | **0.001** | | - | | - | | - | | **200.13*** | | **276.25** | | **0.002** | | **470*** | | **937.5** | | **0.002** | | - | | - | | - | | - | | - | | - |
| Faisal 2018 (24) | | Modified radical mastectomy | | Conventional | | 72 | | **157.8** | | **105*** | | **<0.0001** | | - | | - | | - | | **69.4*** | | **255.5** | | **<0.002** | | **1277.8*** | | **3300** | | **<0.002** | | - | | - | | - | | 13.88% | | 38.88% | | NR |
| Sarwar 2016 (25) | | Modified radical mastectomy | | Conventional | | 100 | | - | | - | | - | | - | | - | | - | | **79.52*** | | **206.34** | | **<0.001** | | - | | - | | - | | - | | - | | - | | - | | - | | - |
| Salama 2020 (26) | | Axillary dissection | | Conventional | | 40 | | **86*** | | **104** | | **<0.001** | | - | | - | | - | | **45*** | | **96** | | **<0.001** | | **847*** | | **1596** | | **<0.001** | | - | | - | | - | | - | | - | | - |
| Deori 2021 (27) | | Axillary dissection | | Conventional | | 70 | | **30.86*** | | **40.63** | | **<0.001** | | - | | - | | - | | - | | - | | - | | **161*** | | **219** | | **<0.001** | | 5.63 | | 5.8 | | 0.462 | | - | | - | | - |
| Mathialagan 2016 (28) | | Selective neck dissection | | Conventional | | 40 | | - | | - | | - | | - | | - | | - | | - | | - | | - | | - | | - | | - | | 2.15 | | 3.3 | | 0.137 | | - | | - | | - |
| Verma 2017 (29) | | Neck dissection | | Conventional | | 40 | | 102 | | 97 | | 0.486 | | 5.85 | | 5.05 | | 0.357 | | **115.55*** | | **127.73** | | **0.007** | | 30 | | 23.5 | | 0.066 | | 2.55 | | 2.5 | | 0.456 | | - | | - | | - |
| Schneider 2018 (30) | | Neck dissection | | Conventional | | 30 | | - | | - | | - | | - | | - | | - | | - | | - | | - | | - | | - | | - | | **4.47*** | | **5.2** | | **0.005** | | - | | - | | - |
| Vaira 2021 (31) | | Neck dissection | | Conventional | | 48 | | 69.1 | | 71.2 | | 0.647 | | 22.17 | | 18.75 | | 0.151 | | **225*** | | **351.2** | | **0.003** | | 375 | | 398.25 | | 0.362 | | - | | - | | - | | - | | - | | - |
| He 2011 (32) | | Total thyroidectomy | | Conventional | | 105 | | **102.8*** | | **150.1** | | **<0.05** | | **5.8*** | | **6.7** | | **<0.05** | | - | | - | | - | | **202.7*** | | **299.7** | | **<0.05** | | - | | - | | - | | - | | - | | - |
| Mourad 2011 (33) | | Total thyroidectomy | | Conventional | | 68 | | **57*** | | **80** | | **<0.001** | | - | | - | | - | | **28*** | | **52** | | **<0.001** | | 35 | | 38 | | 0.87 | | - | | - | | - | | - | | - | | - |
| Yener 2014 (34) | | Total thyroidectomy and lobectomy | | Conventional | | 85 | | **44.9*** | | **69.5** | | **<0.001** | | 2.1 | | 3.2 | | NS | | - | | - | | - | | **37.4*** | | **56.1** | | **<0.001** | | - | | - | | - | | - | | - | | - |
| Aziz 2016 (35) | | Thyroidectomy | | Conventional | | 160 | | **76.7*** | | **117** | | **<0.001** | | - | | - | | - | | - | | - | | - | | - | | - | | - | | - | | - | | - | | - | | - | | - |
| Docimo 2016 (36) | | Total thyroidectomy | | Conventional | | 93 | | 53.97 | | 80.73 | | NR | | 2.59 | | 2.59 | | NR | | - | | - | | - | | **74.31*** | | **97.9** | | **<0.04** | | - | | - | | - | | - | | - | | - |
| Su 2016 (37) | | Total Thyroidectomy | | Conventional | | 93 | | **59.8*** | | **71.6** | | **<0.05** | | **4.2*** | | **4.7** | | **<0.05** | | **41.9*** | | **50.1** | | **<0.05** | | **98.6*** | | **88.4** | | **<0.05** | | - | | - | | - | | - | | - | | - |
|  |  | Total Thyroidectomy | | Conventional (electrocautery) | | 76 | | 59.8 | | 63.1 | | >0.05 | | 4.2 | | 4.6 | | >0.05 | | 41.9 | | 39.8 | | >0.05 | | 98.6 | | 110.2 | | >0.05 | | - | | - | | - | | - | | - | | - |
|  |  | Single Thyroid Lobectomy | | Conventional | | 107 | | **40.2*** | | **46.6** | | **<0.05** | | **3.6*** | | **4.1** | | **<0.05** | | **21.1*** | | **30.2** | | **<0.05** | | **49.5*** | | **55.1** | | **<0.05** | | - | | - | | - | | - | | - | | - |
|  |  | Single Thyroid Lobectomy | | Conventional (electrocautery) | | 98 | | 40.2 | | 39.5 | | >0.05 | | 3.6 | | 3.2 | | >0.05 | | 21.1 | | 24.9 | | >0.05 | | 49.5 | | 41.2 | | >0.05 | | - | | - | | - | | - | | - | | - |
|  |  | Total Thyroidectomy + unilateral LND | | Conventional | | 120 | | **57.1*** | | **72.3** | | **<0.05** | | **4.7*** | | **5.1** | | **<0.05** | | **45.9*** | | **60.4** | | **<0.05** | | **78.6*** | | **82.8** | | **<0.05** | | - | | - | | - | | - | | - | | - |
|  |  | Total Thyroidectomy + unilateral LND | | Conventional (electrocautery) | | 110 | | 57.1 | | 61.2 | | >0.05 | | 4.7 | | 4.9 | | >0.05 | | 45.9 | | 57.9 | | >0.05 | | 78.6 | | 63.1 | | >0.05 | | - | | - | | - | | - | | - | | - |
|  |  | Total thyroidectomy + bilateral LND | | Conventional | | 44 | | **62.3*** | | **75.4** | | **<0.04** | | **5.8*** | | **6.1** | | **<0.05** | | **55.8*** | | **60.4** | | **<0.05** | | **135.1*** | | **154.6** | | **<0.05** | | - | | - | | - | | - | | - | | - |
|  |  | Total thyroidectomy + bilateral LND | | Conventional (electrocautery) | | 46 | | 62.3 | | 71.4 | | >0.05 | | 5.8 | | 5.2 | | >0.05 | | 55.8 | | 57.9 | | >0.05 | | 135.1 | | 126.2 | | >0.05 | | - | | - | | - | | - | | - | | - |
| Anandaravi 2017 (38) | | Near total thyroidectomy | | Conventional | | 34 | | **67.21*** | | **109.6** | | **0.001** | | 3.28 | | 4.2 | | 0.083 | | - | | - | | - | | **28.21*** | | **45.5** | | **0.006** | | - | | - | | - | | - | | - | | - |
|  |  | Hemithyroidectomy | | Conventional | | 34 | | **53.4*** | | **85.25** | | **0.004** | | 3 | | 3 | | NR | | - | | - | | - | | **16*** | | **36.25** | | **0.008** | | - | | - | | - | | - | | - | | - |
| Basurto-Kuba 2017 (39) | | Total thyroidectomy and thyroid lobectomy | | Conventional | | 100 | | **67.32*** | | **88.06** | | **0.0001** | | 2.04 | | 2 | | 0.912 | | **47.2*** | | **68.9** | | **0.025** | | - | | - | | - | | - | | - | | - | | - | | - | | - |
| Shaaban 2017 (40) | | Total thyroidectomy | | Conventional | | 120 | | **76.9*** | | **97.3** | | **<0.001** | | 1.8 | | 1.6 | | 0.342 | | 103.1 | | 102.3 | | 0.884 | | - | | - | | - | | - | | - | | - | | - | | - | | - |
| Uludag 2017 (41) | | Total thyroidectomy | | Conventional | | 40 | | **40.3*** | | **54** | | **0.001** | | - | | - | | - | | - | | - | | - | | - | | - | | - | | - | | - | | - | | - | | - | | - |
| Ahmad 2018 (42) | | Total or near-total thyroidectomy | | Conventional | | 120 | | **44.91*** | | **73.86** | | **0** | | **2.31*** | | **3.47** | | **0** | | - | | - | | - | | **36.61*** | | **58.11** | | **0** | | - | | - | | - | | - | | - | | - |
| Jamil 2019a (43) | | Thyroidectomy | | Conventional | | 60 | | - | | - | | - | | **2.9*** | | **4.2** | | **0.00001** | | - | | - | | - | | - | | - | | - | | - | | - | | - | | - | | - | | - |
| Jamil 2019b (44) | | Thyroidectomy | | Conventional | | 60 | | - | | - | | - | | - | | - | | - | | **70.03*** | | **96.43** | | **0.00002** | | - | | - | | - | | - | | - | | - | | - | | - | | - |
| Kadem 2019 (45) | | Thyroidectomy | | Conventional | | 60 | | **65.5*** | | **50.1** | | **0.001** | | 1.01 | | 1.07 | | 0.32 | | - | | - | | - | | 63.02 | | 62.5 | | 0.33 | | - | | - | | - | | - | | - | | - |
| Ansari 2020 (46) | | Thyroid Surgery (Any type) | | Conventional | | 100 | | **84.38*** | | **98.16** | | **<0.001** | | **2.48*** | | **3.12** | | **<0.001** | | **64.5*** | | **76** | | **0.003** | | - | | - | | - | | - | | - | | - | | - | | - | | - |
| Buzdar 2020 (47) | | Total Thyroidectomy | | Conventional | | 94 | | **41.94*** | | **70.45** | | **<0.001** | | **2.74*** | | **3.89** | | **<0.001** | | - | | - | | - | | - | | - | | - | | - | | - | | - | | - | | - | | - |
| Ali 2011 (48) | | Tonsillectomy | | Conventional | | 60 | | **4.2** | | **3.57*** | | **<0.05** | | - | | - | | - | | 2.4 | | 3.43 | | 0.1 | | - | | - | | - | | 4.97 | | 5.43 | | 0.096 | | - | | - | | - |
| Pajic-Penavic 2013 (49) | | Tonsillectomy | | Conventional | | 100 | | - | | - | | - | | - | | - | | - | | - | | - | | - | | - | | - | | - | | 3.7 | | 3.52 | | 0.1201 | | - | | - | | - |
| Arbin 2017 (50) | | Tonsillectomy | | Conventional | | 40 | | - | | - | | - | | - | | - | | - | | - | | - | | - | | - | | - | | - | | 6.08 | | 7.03 | | 0.65 | | - | | - | | - |
| Karimi 2017 (51) | | Tonsillectomy | | Conventional | | 64 | | **7.12*** | | **12.46** | | **<0.0001** | | - | | - | | - | | **9.59*** | | **74.38** | | **<0.0001** | | - | | - | | - | | 3.88 | | 6.19 | | NS | | - | | - | | - |
| Basu 2019 (52) | | Tonsillectomy | | Conventional | | 128 | | **20.15*** | | **36.96** | | **<0.001** | | - | | - | | - | | **42.43*** | | **125.62** | | **<0.001** | | - | | - | | - | | 2.15 | | 2.54 | | 0.0568 | | - | | - | | - |
| Sah 2019 (53) | | Tonsillectomy | | Conventional | | 38 | | - | | - | | - | | - | | - | | - | | 13.94 | | 13.91 | | 0.974 | | - | | - | | - | | **3.38*** | | 4.77 | | <0.001 | | - | | - | | - |
| Kwek 2020 (54) | | Tonsillectomy | | Conventional | | 20 | | 25† | | 25† | | 0.564 | | - | | - | | - | | - | | - | | - | | - | | - | | - | | - | | - | | - | | - | | - | | - |
| Subasi 2021 (55) | | Tonsillectomy | | Conventional (Coblation) | | 57 | | **6*** | | **23.4** | | **0** | | - | | - | | - | | - | | - | | - | | - | | - | | - | | 3.3 | | 3.6 | | 0.85 | | - | | - | | - |
|  |  |  |  | Conventional (Cold dissection) | | 58 | | **6*** | | **21.2** | | **0** | | - | | - | | - | | - | | - | | - | | - | | - | | - | | 3.3 | | 3.6 | | 0.85 | | - | | - | | - |
| **ABP Comparators** | | | | | | | | | | | | | | | | | | | | | | | | | | | | | | | | | | | | | | | | | | |
| Peker 2013 (15) | | Hemorrhoidectomy | | Advanced bipolar | | 46 | | 12.6 | | 12.67 | | NR | | - | | - | | - | | - | | - | | - | | - | | - | | - | | - | | - | | - | | - | | - | | - |
| Hwang 2014 (56) | | Open thyroidectomy with central LND | | Advanced bipolar | | 126 | | 106.6 | | 104.4 | | 0.52 | | 3.1 | | 3.3 | | 0.5 | | - | | - | | - | | **123.6** | | **106.8*** | | **0.01** | | - | | - | | - | | - | | - | | - |
| Ciftci 2016 (57) | | Total thyroidectomy | | Advanced bipolar | | 201 | | **72*** | | **84** | | **<0.01** | | 2 | | 2 | | NS | | - | | - | | - | | - | | - | | - | | 2.6 | | 2.4 | | NS | | - | | - | | - |
| Uludag 2017 (41) | | Total thyroidectomy | | Advanced bipolar | | 50 | | 40.3 | | 44.5 | | NR | | **2.31*** | | **3.47** | | **0** | | - | | - | | - | | - | | - | | - | | - | | - | | - | | - | | - | | - |
| Back 2019 (58) | | Thyroidectomy | | Advanced bipolar (Thunderbeat) | | 50 | | **18.08** | | **16.68*** | | **0.031** | | 2.24 | | 2.4 | | 0.263 | | - | | - | | - | | 38.88 | | 42.24 | | 0.716 | | - | | - | | - | | - | | - | | - |
|  |  |  |  | Advanced bipolar (LigaSure) | | 50 | | **18.08*** | | **19.42** | | **0.031** | | 2.24 | | 2.36 | | 0.263 | | - | | - | | - | | 38.88 | | 41.32 | | 0.716 | | - | | - | | - | | - | | - | | - |
| Papavramidis 2020 (59) | | Total Thyroidectomy | | Advanced bipolar (LigaSure Small Jaw) | | 90 | | 82.88 | | 81.79 | | NS | | - | | - | | - | | - | | - | | - | | - | | - | | - | | 3.67 | | 3.8 | | NS | | - | | - | | - |
|  |  |  |  | Advanced bipolar (Thunderbeat) | | 90 | | **82.88** | | **73.02*** | | **<0.001** | | - | | - | | - | | - | | - | | - | | - | | - | | - | | 3.67 | | 3.75 | | NS | | - | | - | | - |
|  |  |  |  | Advanced bipolar (LigaSure Exact) | | 90 | | **82.88** | | **50.39*** | | **<0.001** | | - | | - | | - | | - | | - | | - | | - | | - | | - | | 3.67 | | 3.73 | | NS | | - | | - | | - |
| Kim 2021 (60) | | Open Thyroidectomy | | Advanced bipolar | | 200 | | 54.2 | | 50.2 | | 0.238 | | 2.5 | | 2.5 | | 0.875 | | - | | - | | - | | - | | - | | - | | - | | - | | - | | - | | - | | - |

Additional RCTs are those that were either published after the most recent systematic review and meta-analyses or those that were published during the search period for the included meta-analyses but were not included by them. Mean values are provided for HS and CD unless otherwise stated.

Asterisks (*) indicate a significant association (p < 0.05). Asterisks are assigned to the intervention/comparator with the significant advantage. Statistically significant outcomes are also bolded.

† Indicates a median value

Abbreviations: CD = comparator device; HS = Harmonic scalpel; LND = lymph node dissection; NR = not reported; NS = not significant; VAS = visual analogue scale.

Supplementary Table 3: Summary of included orphan RCTs

| **Study** | **Procedure** | **Comparator Type** | **Total Population Size (n)** | **Operating Time (min)** | | | **Length of Stay (days)** | | | **Bleeding (mL)** | | | **Drainage Volume (mL)** | | | | **Pain (VAS)** | | | **Operative complications (% of patients)** | | | |
| --- | --- | --- | --- | --- | --- | --- | --- | --- | --- | --- | --- | --- | --- | --- | --- | --- | --- | --- | --- | --- | --- | --- | --- |
|  |  |  |  | **HS** | **CD** | **p** | **HS** | **CD** | **p** | **HS** | **CD** | **p** | | **HS** | CD | **p** | **HS** | **CD** | **p** | | **HS** | **CD** | **p** |
| **Conventional Comparators** | |  |  |  |  |  |  |  |  |  |  |  | |  |  |  |  |  |  | |  |  |  |
| Duscher 2019 (61) | Abdominoplasty | Conventional | 57 | 88 | 110 | NS | - | - | - | **100.2** | **39.3*** | **<0.01** | | - | - | - | - | - | - | | - | - | - |
| Qaiser 2021 (62) | Appendectomy | Conventional | 110 | **1.56*** | **21.07** | **<0.001** | - | - | - | **0‡*** | **24‡** | **<0.001** | | - | - | - | - | - | - | | - | - | - |
| Tremp 2012 (63) | Breast capsulectomy | Conventional | 10 | 31 | 38 | 0.28 | - | - | - | - | - | - | | 182 | 220 | 0.42 | 2.7 | 2.4 | 0.58 | | - | - | - |
| Burdette 2011 (64) | Breast reduction | Conventional | 62 | **40.1** | **31*** | **0.02** | - | - | - | - | - | - | | - | - | - | 4.5 | 3.8 | 0.14 | | - | - | - |
| Hanyong 2015 (65) | Hepatectomy | Conventional | 160 | 120.9 | 125.6 | 0.516 | **8.7*** | **9.7** | **0.045** | **348*** | **420** | **0.042** | | - | - | - | - | - | - | | **22.5%*** | **41.3%** | **0.011** |
| Sultan 2019 (66) | Hepatectomy | Conventional | 72 | **360*** | **440** | **0.001** | 6 | 7 | 0.332 | 300 | 500 | 0.113 | | - | - | - | - | - | - | | 22.2% | 27.8% | 0.952 |
| El Shobary 2017 (67) | Parenchymal liver transection | Conventional | 80 | 472† | 465† | 0.85 | 8† | 7† | 0.16 | 500† | 400† | 0.21 | | 550.4† | 560† | 0.74 | - | - | - | | **22.5%*** | **45.0%** | **0.03** |
| Olmez 2012 (68) | Liver transplant | Conventional | 16 | 34 | 37 | 0.6 | - | - | - | - | - | - | | - | - | - | - | - | - | | - | - | - |
| Fitz-Gerald 2013 (69) | Hysterectomy | Conventional | 40 | 97.4 | 91.6 | 0.63 | 2.5 | 2.5 | 0.89 | **62.6*** | **136.1** | **0.006** | | - | - | - | - | - | - | | - | - | - |
| Choi 2018 (70) | Total Hysterectomy | Conventional | 40 | 68 | 59 | 0.081 | 2.2 | 2.3 | 0.799 | 51.4 | 46 | 0.82 | | - | - | - | - | - | - | | 0.0% | 10.0% | 0.487 |
| Rothmund 2013 (71) | Laparoscopic supracervical hysterectomy | Conventional | 60 | 8.3 | 8.8 | 0.31 | - | - | - | - | - | - | | - | - | - | - | - | - | | - | - | - |
| Litta 2010 (72) | Uterine myomectomy | Conventional | 160 | **71.8*** | **88.8** | **0** | **2.3*** | **2.7** | **<0.0001** | **135.2*** | **182.8** | **0.004** | | - | - | - | **4.4*** | **5.6** | **0.0001** | | - | - | - |
| Uzunoglu 2012 (73) | Pancreatectomy and lymphadenectomy | Conventional | 101 | 316† | 319† | 0.95 | 15† | 15† | 0.76 | 350† | 350† | 0.67 | | - | - | - | - | - | - | | 56.1% | 53.3% | 0.7 |
| Landoni 2021 (74) | Distal Pancreatectomy | Conventional | 145 | - | - | - | 8 | 8 | 0.88 | - | - | - | | - | - | - | - | - | - | | - | - | - |
| He 2014 (75) | Parathyroidectomy | Conventional | 56 | **58.5*** | **99.6** | **<0.05** | 10.8 | 11.5 | >0.05 | **20*** | **108.6** | **<0.05** | | **132.7*** | **267.7** | **<0.05** | **3.9*** | **6.4** | **<0.05** | | - | - | - |
| Deganello 2014 (76) | Parotid surgery | Conventional | 130 | 146.9 | 151.6 | 0.8 | - | - | - | - | - | - | | 69 | 78 | 0.5 | - | - | - | | - | - | - |
| Uysal 2019 (77) | Radial Artery Harvesting | Conventional | 45 | **16*** | **25.1** | **0.001** | - | - | - | - | - | - | | **22.3*** | **35** | **0.0001** | - | - | - | | - | - | - |
| Fritz 2016 (78) | Resection of advanced oral cancer (OSCC) | Conventional | 34 | 140 | 159 | 0.21 | 14 | 15 | NS | **260*** | **403** | **0.08** | | 160 | 119 | NS | - | - | - | | - | - | - |
| **ABP Comparators** | |  |  |  |  |  |  |  |  |  |  |  | |  |  |  |  |  |  | |  |  |  |
| Pogorelic 2017 (79) | Appendectomy | Advanced bipolar (LigaSure) | 68 | **35†** | **30†*** | **0.046** | **4†** | **3†*** | **0.012** | - | - | - | | - | - | - | - | - | - | | 11.0% | 3.0% | 0.098 |
|  |  | Advanced bipolar (MiSeal) | 67 | **35†** | **25†*** | **0.046** | **4†** | **3†*** | **0.012** | - | - | - | | - | - | - | - | - | - | | 11.0% | 0.0% | 0.098 |
| Roy 2018 (80) | Hysterectomy | Advanced bipolar (LigaSure) | 40 | 48.8 | 46.5 | NR | 72.1 | 71.8 | NR | - | - | - | | - | - | - | - | - | - | | - | - | - |
|  |  | Advanced bipolar (Thunderbeat) | 40 | 48.8 | 49.5 | NR | 72.1 | 77.5 | NR | - | - | - | | - | - | - | - | - | - | | - | - | - |
| Tsamis 2015 (81) | Laparoscopic sleeve gastrectomy | Advanced bipolar | 94 | 40 | 45 | 0.199 | - | - | - | - | - | - | | - | - | - | - | - | - | | 17.6% | 4.7% | 0.051 |
| Pastore 2013 (82) | Prostatectomy | Advanced bipolar | 132 | 113.1 | 108.1 | 0.748 | - | - | - | 286 | 290 | 0.9132 | | - | - | - | - | - | - | | 22.5% | 19.5% | NR |
| Toishi 2014 (83) | Thoracoscopic lobectomy | Advanced bipolar | 58 | 239 | 258 | 0.5557 | - | - | - | 155 | 122 | 0.2965 | | 395 | 437 | 0.6625 | - | - | - | | 41.6% | 35.3% | 0.8986 |

Orphan RCTs are from procedures for which there was no eligible systematic review and meta-analyses identified for that procedure type. Mean values are provided for HS and CD unless otherwise stated.

Asterisks (*) indicate a significant association (p < 0.05). Asterisks are assigned to the intervention/comparator with the significant advantage. Statistically significant outcomes are also bolded.

† Indicates a median value

‡ Indicates the number of patients who experience bleeding

Abbreviations: CD = comparator device; HS = Harmonic scalpel; LTBR = low-temperature bipolar radiofrequency ablation of the tongue base; NR = not reported; NS = not significant; SMILE-R = submucosal minimally invasive lingual excision with radiofrequency; VAS = visual analogue scale.

Supplementary Table 4: Methodological quality assessment of included systematic reviews using AMSTAR-2

| **Study** | **1** | **2** | **3** | **4** | **5** | **6** | **7** | **8** | **9** | **10** | **11** | **12** | **13** | **14** | **15** | **16** | **Overall AMSTAR 2 Score** |
| --- | --- | --- | --- | --- | --- | --- | --- | --- | --- | --- | --- | --- | --- | --- | --- | --- | --- |
| **Aires 2018** | Yes | No | No | Partial Yes | No | No | No | No | Yes | No | No | Yes | No | No | No | No | Critically Low |
| **Alexiou 2011** | Yes | No | No | No | Yes | Yes | No | Partial Yes | Yes | No | No | No | Yes | Yes | Yes | Yes | Critically Low |
| **Balciscueta 2021** | Yes | Yes | No | Partial Yes | No | Yes | No | Partial Yes | Yes | No | No | Yes | Yes | Yes | No | Yes | Critically Low |
| **Cannizzaro 2016** | Yes | No | No | No | No | No | No | No | No | No | No | No | No | Yes | No | No | Critically Low |
| **Chen 2014** | Yes | No | No | No | Yes | Yes | No | Partial Yes | Yes^a^ | No | No^a^ | Yes | Yes | Yes | Yes | Yes | Critically Low |
| **Cheng 2016 (Mastectomy)** | Yes | No | No | Partial Yes | Yes | Yes | No | Partial Yes | Yes | No | No | Yes | Yes | Yes | No | No | Critically Low |
| **Cheng 2015 (Gastrectomy)** | Yes | No | No | No | Yes | Yes | No | Yes | Yes | No | No | Yes | Yes | No | No | No | Critically Low |
| **Cheng 2015 (Thyroidectomy)** | Yes | No | No | No | Yes | Yes | No | Yes | Yes | No | No | Yes | Yes | No | No | No | Critically Low |
| **Cheng 2016 (Thyroidectomy)** | Yes | No | No | Partial Yes | Yes | Yes | No | Partial Yes | Yes | No | No | Yes | Yes | Yes | No | No | Critically Low |
| **Cirocchi 2010** | Yes | No | No | Partial Yes | Yes | No | No | Partial Yes | No | No | No | No | No | No | No | Yes | Critically Low |
| **Ecker 2010** | Yes | Partial Yes | No | No | Yes | No | No | Partial Yes | Yes | No | No | No | Yes | Yes | Yes | Yes | Critically Low |
| **Hua 2019** | Yes | No | No | No | Yes | Yes | No | Partial Yes | Yes | No | No | Yes | Yes | Yes | Yes | Yes | Critically Low |
| **Jiang 2017** | Yes | No | No | Partial Yes | No | Yes | No | Partial Yes | Yes | No | No | Yes | Yes | Yes | Yes | Yes | Critically Low |
| **Kim 2021** | Yes | No | No | No | No | No | No | No | No | No | No | No | No | Yes | No | Yes | Critically Low |
| **Melck 2010** | Yes | No | No | Partial Yes | Yes | Yes | No | Partial Yes | No | Yes | No | Yes | Yes | Yes | Yes | No | Critically Low |
| **Mushaya 2014** | Yes | No | No | Partial Yes | No | Yes | No | Partial Yes | No | No | No | Yes | No | Yes | No | Yes | Critically Low |
| **Ren 2015** | Yes | Partial Yes | No | Partial Yes | No | Yes | No | No | Yes | No | No | No | Yes | Yes | Yes | Yes | Critically Low |
| **Revelli 2016** | Yes | No | No | No | Yes | Yes | No | No | Yes | No | No | No | No | No | Yes | Yes | Critically Low |
| **Sasi 2010** | Yes | No | No | No | No | No | No | Partial Yes | Yes | No | No | No | Yes | No | Yes | No | Critically Low |
| **Sun 2015** | Yes | No | No | Partial Yes | No | Yes | No | Partial Yes | Yes | No | No | No | No | Yes | Yes | Yes | Critically Low |
| **Tou 2011** | Yes | Yes | No | Partial Yes | Yes | Yes | Yes | Partial Yes | Yes | Yes | No | No | Yes | Yes | Yes | Yes | Low |
| **Upadhyaya 2016** | Yes | No | No | Partial Yes | Yes | No | No | Partial Yes | Yes | No | No | No | Yes | Yes | No | No | Critically Low |
| **Xiong 2012** | Yes | No | No | Partial Yes | Yes | Yes | No | Yes | Yes | No | No | Yes | Yes | Yes | Yes | Yes | Critically Low |
| **Zhang 2018** | Yes | No | No | No | No | No | No | Partial Yes | Yes^a^ | No | No^a^ | Yes | No | Yes | No | Yes | Critically Low |

1. Did the research questions and inclusion criteria for the review include the components of PICO?

2. Did the report of the review contain an explicit statement that the review methods were established prior to the conduct of the review and did the report justify any significant deviations from the protocol?

3. Did the review authors explain their selection of the study designs for inclusion in the review?

4. Did the review authors use a comprehensive literature search strategy?

5. Did the review authors perform study selection in duplicate?

6. Did the review authors perform data extraction in duplicate?

7. Did the review authors provide a list of excluded studies and justify the exclusions?

8. Did the review authors describe the included studies in adequate detail?

9. Did the review authors use a satisfactory technique for assessing the risk of bias (RoB) in individual studies that were included in the review?

10. Did the review authors report on the sources of funding for the studies included in the review?

11. If meta-analysis was performed did the review authors use appropriate methods for statistical combination of results?

12. If meta-analysis was performed, did the review authors assess the potential impact of RoB in individual studies on the results of the meta-analysis or other evidence synthesis?

13. Did the review authors account for RoB in individual studies when interpreting/ discussing the results of the review?

14. Did the review authors provide a satisfactory explanation for, and discussion of, any heterogeneity observed in the results of the review?

15. If they performed quantitative synthesis did the review authors carry out an adequate investigation of publication bias (small study bias) and discuss its likely impact on the results of the review?

16. Did the review authors report any potential sources of conflict of interest, including any funding they received for conducting the review?

a Chen, 2014 and Zhang, 2018 both included non-randomized studies, but stratified results by study design.

Supplementary Table 5: Grade assessment for included meta-analyses

| **Study** | **Starting score** | **Issues impacting GRADE score^1^** | | | | | **Overall score^2^** |
| --- | --- | --- | --- | --- | --- | --- | --- |
|  |  | **1. Risks of bias** | **2. Inconsistency** | **3. Indirectness** | **4. Imprecision** | **5. Publication bias** |  |
| **HEMORROIDECTOMY** |  |  |  |  |  |  |  |
| **Balciscueta 2021 - Pain** | HIGH | Per author-reported risk of bias assessments, no issues identified | I2 was >99% in both analyses included, with significant p values (<0.05) Downgraded | No issues identified | No issues identified | Publication bias not assessed.  Downgraded | Low |
| **Mushaya 2014 - Pain** | HIGH | Per author-reported risk of bias assessments, no issues identified | Q = 33.2; p=<0.001 Heterogeneity was unacceptably large, driven by two studies which showed rather large effect sizes Downgraded | No issues identified | No issues identified | No issues identified | Moderate |
| **Mushaya 2014 - Overall complications** | HIGH | Per author-reported risk of bias assessments, no issues identified | No issues identified | No issues identified | No issues identified | No issues identified | High |
| **Mushaya 2014 - Operating time** | HIGH | Per author-reported risk of bias assessments, no issues identified | Q = 121.3; P<0.001 Heterogeneity was unacceptably large, driven by two studies which showed rather large effect sizes Downgraded | No issues identified | mean difference = -1.84 (-3.6 to-0.13); p=0.035 Downgraded given that confidence interval is approaching threshold | No issues identified | Low |
| **Mushaya 2014 - Length of stay** | HIGH | Per author-reported risk of bias assessments, no issues identified | Q = 9.6; P<0.008 Heterogeneity was unacceptably large, driven by one study which showed a rather large effect size Downgraded | No issues identified | mean difference = -0.6 (-1.3 to 0.15); p=0.121 (not statistically significant). Only 3 studies included in analysis, sample size NR Downgraded | No issues identified | Low |
| **THYROIDECTOMY** |  |  |  |  |  |  |  |
| **Aires 2018 - Operating time** | HIGH | Per author-reported risk of bias assessments, no issues identified | I2 = 96%, with a significant p value (p<0.0001) Downgraded | No issues identified | No issues identified | No issues identified | Moderate |
| **Aires 2018 - Intraoperative blood loss** | HIGH | Per author-reported risk of bias assessments, no issues identified | I2 = 98%, with a significant p value (p<0.0001) Downgraded | No issues identified | No issues identified | No issues identified | Moderate |
| **Cannizzaro 2016 - Operating time** | HIGH | Several RCTs did not give a detailed description of their randomization and blinding procedures. There was no detailed information on methods of data acquisition and measurement. Downgraded | Heterogeneity mentioned but specific information NR; fixed-effect model used Downgraded | No issues identified | No issues identified | No issues identified | Low |
| **Cannizzaro 2016 - Blood Loss** | HIGH | Several RCTs did not give a detailed description of their randomization and blinding procedures. There was no detailed information on methods of data acquisition and measurement. Downgraded | Heterogeneity mentioned but specific information NR; fixed-effect model used Downgraded | No issues identified | No issues identified | No issues identified | Low |
| **Cannizzaro 2016 - Overall complications** | HIGH | Several RCTs did not give a detailed description of their randomization and blinding procedures. There was no detailed information on methods of data acquisition and measurement. Downgraded | Heterogeneity mentioned but specific information NR; fixed-effect model used Downgraded | No issues identified | Wide 95% CI: OR = 0.961 (0.638 to 1.447; p=.849); large sample size, n=2394 Downgraded | No issues identified | Very Low |
| **Cannizzaro 2016 - Pain** | HIGH | Several RCTs did not give a detailed description of their randomization and blinding procedures. There was no detailed information on methods of data acquisition and measurement. Downgraded | Heterogeneity mentioned but specific information NR; fixed-effect model used Downgraded | No issues identified | No issues identified | No issues identified | Low |
| **Cannizzaro 2016 - Length of stay** | HIGH | Several RCTs did not give a detailed description of their randomization and blinding procedures. There was no detailed information on methods of data acquisition and measurement. Downgraded | Heterogeneity mentioned but specific information NR; fixed-effect model used Downgraded | No issues identified | mean difference = 0.410 (-0.693 to -0.127; p=.005); large sample size, n=1819 Downgraded given that confidence interval is approaching threshold | No issues identified | Very Low |
| **Cheng 2015 - Operating time** | HIGH | Per author-reported risk of bias assessments, no issues identified | I2 = 96%, with a significant p value (p<0.00001) Downgraded | No issues identified | No issues identified | No issues identified | Moderate |
| **Cheng 2015 - Intraoperative blood loss** | HIGH | Per author-reported risk of bias assessments, no issues identified | I2 = 98%, with a significant p value (p<0.00001) Downgraded | No issues identified | No issues identified | No issues identified | Moderate |
| **Cheng 2015 - Pain** | HIGH | Per author-reported risk of bias assessments, no issues identified | I2 = 85%, with a significant p value (p=0.001) Downgraded | No issues identified | mean difference = -1.33 (-1.99 to -0.67); only 3 studies included, n=292 Downgraded | No issues identified | Low |
| **Cheng 2015 - Length of stay** | HIGH | Per author-reported risk of bias assessments, no issues identified | I2 = 98%, with a significant p value (p<0.00001) Downgraded | No issues identified | mean difference = -0.68 (-1.16 to -0.20); large sample size, n=1934 Downgraded given that confidence interval is approaching threshold | No issues identified | Low |
| **Cheng 2015 - Drainage volume** | HIGH | Per author-reported risk of bias assessments, no issues identified | I2 = 99%, with a significant p value (p<0.00001) Downgraded | No issues identified | mean difference = -29.38 (-52.46 to -6.30); only 3 studies included, n=292 Downgraded | No issues identified | Low |
| **Cirocchi 2010 - Operating time** | HIGH | Assessment of methodological quality mentioned in methods, no results reported, no clear rationale to downgrade | I2 = 84%, with a significant p value (p<0.00001) Downgraded | No issues identified | No issues identified | No issues identified | Moderate |
| **Ecker 2010 - Drainage volume** | HIGH | Per author-reported risk of bias assessments, no issues identified | I2 = 77%, with a significant p value (p=0.005) Downgraded | No issues identified | mean difference = -9.27 (-21.27 to 2.73); large sample size, n=519 Downgraded | The results of a funnel plot suggest asymmetry. Grey literature not searched. Downgraded | Very Low |
| **Ecker 2010 - Operating time** | HIGH | Per author-reported risk of bias assessments, no issues identified | I2 = 87%, with a significant p value (p<0.00001) Downgraded | No issues identified | No issues identified | The results of a funnel plot suggest asymmetry. Grey literature not searched. Downgraded | Low |
| **Ecker 2010 - Intraoperative blood loss** | HIGH | Per author-reported risk of bias assessments, no issues identified | I2 = 62%, with a significant p value (p=0.001) Downgraded | No issues identified | No issues identified | The results of a funnel plot suggest asymmetry. Grey literature not searched. Downgraded | Low |
| **Ecker 2010 - Overall complications** | HIGH | Per author-reported risk of bias assessments, no issues identified | I2 = 59%, with a significant p value (p=0.003) Downgraded | No issues identified | Wide 95% CI: OR = 0.97 (0.59 to 1.60); large sample size, n=1202 Downgraded | The results of a funnel plot suggest asymmetry. Grey literature not searched. Downgraded | Very Low |
| **Ecker 2010 - Pain** | HIGH | Per author-reported risk of bias assessments, no issues identified | I2 = 77%, with a significant p value (p=0.001) Downgraded | No issues identified | mean difference = -0.86 (-1.60 to -0.13); medium sample size, n=240 Downgraded given that confidence interval is approaching threshold | The results of a funnel plot suggest asymmetry. Grey literature not searched. Downgraded | Very Low |
| **Ecker 2010 - Length of stay** | HIGH | Per author-reported risk of bias assessments, no issues identified | No issues identified | No issues identified | mean difference = -0.12 (-0.25 to 0.00); large sample size, n=488 Downgraded given that confidence interval is approaching threshold | The results of a funnel plot suggest asymmetry. Grey literature not searched. Downgraded | Low |
| **Melck and Wiseman, 2010 - Operating time** | HIGH | Per author-reported risk of bias assessments, no issues identified | The χ2 test for heterogeneity was significant with a P-value of <.001 Downgraded | No issues identified | No issues identified | No issues identified | Moderate |
| **Revelli 2016 - Operating time** | HIGH | Per author-reported risk of bias assessments, no issues identified | I2 = >92%, with a significant p value (p<0.00001) Downgraded | No issues identified | No issues identified | No issues identified | Moderate |
| **Revelli 2016 - Length of stay** | HIGH | Per author-reported risk of bias assessments, no issues identified | I2 = >90%, with a significant p value (p<0.00001) Downgraded | No issues identified | mean difference = -0.17 (-0.54 to 0.20); large sample sizes Downgraded | No issues identified | Low |
| **Revelli 2016 - Intraoperative blood loss** | HIGH | Per author-reported risk of bias assessments, no issues identified | I2 = 93%, with a significant p value (p<0.00001) Downgraded | No issues identified | No issues identified | No issues identified | Moderate |
| **Revelli 2016 - Drainage volume** | HIGH | Per author-reported risk of bias assessments, no issues identified | I2 = >80%, with a significant p value (p<0.00001) Downgraded | No issues identified | mean difference = -12.90 (-22.83 to -2.98); large sample sizes Downgraded given that confidence interval is approaching threshold | No issues identified | Low |
| **Revelli 2016 - Pain** | HIGH | Per author-reported risk of bias assessments, no issues identified | harmonic focus: I2 = 92%, p=0.0003 Downgraded | No issues identified | No issues identified | No issues identified | Moderate |
| **Upadhyaya 2016 - Operating time** | HIGH | Per author-reported risk of bias assessments, no issues identified | I2 = >92%, with a significant p value (p<0.00001) Downgraded | No issues identified | mean difference = -8.79 (-15.91 to -1.67); large sample size, n=981 Downgraded given that confidence interval is approaching threshold | No issues identified | Low |
| **Upadhyaya 2016 - Intraoperative blood loss** | HIGH | Per author-reported risk of bias assessments, no issues identified | I2 = 75%, with a significant p value (p=0.04) Downgraded | No issues identified | mean difference = -6.07 (-21.75 to 9.61); only 2 studies included, n=140 Downgraded | No issues identified | Low |
| **Upadhyaya 2016 - Length of stay** | HIGH | Per author-reported risk of bias assessments, no issues identified | No issues identified | No issues identified | mean difference = -0.00 (-0.04 to 0.04); large sample size, n=751 Downgraded | No issues identified | Moderate |
| **TONSILLECTOMY** |  |  |  |  |  |  |  |
| **Alexiou 2011 - Operating time** | HIGH | Methodological quality assessed according to a modified Jadad score. 61% had a score of 3 or higher; only 4 studies had a low score of 1 (<12%) Downgraded | I2 = 100%, with a significant p value (p<0.001) Downgraded | No issues identified | mean difference = -0.10 (-6.26 to 6.05); large sample size, n=655 Downgraded | No issues identified | Very Low |
| **Alexiou 2011 - Intraoperative blood loss** | HIGH | Methodological quality assessed according to a modified Jadad score. 61% had a score of 3 or higher; only 4 studies had a low score of 1 (<12%) Downgraded | I2 = 98%, with a significant p value (p<0.001) Downgraded | No issues identified | No issues identified | No issues identified | Low |
| **Alexiou 2011 - Pain** | HIGH | Methodological quality assessed according to a modified Jadad score. 61% had a score of 3 or higher; only 4 studies had a low score of 1 (<12%) Downgraded | I2 = 95%, with a significant p value (p<0.001) Downgraded | No issues identified | standard mean difference = -0.38 (-1.20 to 0.43); large sample size, n=517 Downgraded | No issues identified | Very Low |
| **GASTRECTOMY** |  |  |  |  |  |  |  |
| **Chen 2014 - Operating time** | HIGH | Per author-reported risk of bias assessments, no issues identified | I2 = 91%, with a significant p value (p<0.00001) Downgraded | No issues identified | No issues identified | No issues identified | Moderate |
| **Chen 2014 - Overall complications** | HIGH | Per author-reported risk of bias assessments, no issues identified | No issues identified | No issues identified | Wide 95% CI: RR = 0.75 (0.44 to 1.26); large sample size, n=247 Downgraded | No issues identified | Moderate |
| **Chen 2014 - Intraoperative blood loss** | HIGH | Per author-reported risk of bias assessments, no issues identified | I2 = 93%, with a significant p value (p<0.00001) Downgraded | No issues identified | No issues identified | No issues identified | Moderate |
| **Chen 2014 - Drainage volume** | HIGH | Per author-reported risk of bias assessments, no issues identified | I2 = 84%, with a significant p value (p=0.01) Downgraded | No issues identified | No issues identified | No issues identified | Moderate |
| **Cheng 2015 - Operating time** | HIGH | Per author-reported risk of bias assessments, no issues identified | I2 = 91%, with a significant p value (p<0.00001) Downgraded | No issues identified | No issues identified | No issues identified | Moderate |
| **Cheng 2015 - Overall Complications** | HIGH | Per author-reported risk of bias assessments, no issues identified | No issues identified | No issues identified | Wide 95% CI: RR = 0.58 (0.3 to 1.0); large sample size, n=464 Downgrade | No issues identified | Moderate |
| **Cheng 2015 - Intraoperative blood loss** | HIGH | Per author-reported risk of bias assessments, no issues identified | I2 = 86%, with a significant p value (p<0.00001) Downgraded | No issues identified | No issues identified | No issues identified | Moderate |
| **Cheng 2015 - Drainage volume** | HIGH | Per author-reported risk of bias assessments, no issues identified | I2 = 94%, with a significant p value (p<0.00001) Downgraded | No issues identified | No issues identified | No issues identified | Moderate |
| **Cheng 2015 - Length of stay** | HIGH | Per author-reported risk of bias assessments, no issues identified | I2 = 65%, not statistically significant (p=0.06) Downgraded | No issues identified | Wide 95% CI: mean difference = -0.63 (-2.48 to 1.23); only 3 studies included, n=162 Downgraded | No issues identified | Low |
| **Sun 2015 - Operating time** | HIGH | Per author-reported risk of bias assessments, no issues identified | I2 = 95%, with a significant p value (p<0.00001) Downgraded | No issues identified | mean difference = -24.99 (-45.97 to -3.00); large sample size, n=487 Downgraded given that confidence interval is approaching threshold | No issues identified | Low |
| **Sun 2015 - Intraoperative blood loss** | HIGH | Per author-reported risk of bias assessments, no issues identified | I2 = 92%, with a significant p value (p<0.00001) Downgraded | No issues identified | No issues identified | No issues identified | Moderate |
| **Sun 2015 - Drainage volume** | HIGH | Per author-reported risk of bias assessments, no issues identified | I2 = 77%, with a significant p value (p=0.04) Downgraded | No issues identified | Wide 95% CI: mean difference = -292.32 (-708.32 to 123.69); only 2 studies included, n=293 Downgraded | No issues identified | Low |
| **Sun 2015 - Length of stay** | HIGH | Per author-reported risk of bias assessments, no issues identified | No issues identified | No issues identified | mean difference = -2.19 (-3.97 to-0.24); only 2 studies included, n=100 Downgraded | No issues identified | Moderate |
| **MASTECTOMY** |  |  |  |  |  |  |  |
| **Cheng 2016 - Intraoperative blood loss** | HIGH | Per author-reported risk of bias assessments, no issues identified | I2 = 99%, with a significant p value (p<0.00001) Downgraded | No issues identified | No issues identified | No issues identified | Moderate |
| **Cheng 2016 - Drainage volume** | HIGH | Per author-reported risk of bias assessments, no issues identified | I2 = 87%, with a significant p value (p<0.00001) Downgraded | No issues identified | No issues identified | No issues identified | Moderate |
| **Cheng 2016 - Length of stay** | HIGH | Per author-reported risk of bias assessments, no issues identified | I2 = 98%, with a significant p value (p<0.00001) Downgraded | No issues identified | mean difference = -1.38 (-2.38 to -0.38); only 4 studies included, n=370 Downgraded given that confidence interval is approaching threshold | No issues identified | Low |
| **Cheng 2016 - Operating time** | HIGH | Per author-reported risk of bias assessments, no issues identified | I2 = 83%, not statistically significant (p=0.09) Downgraded | No issues identified | mean difference = -5.07 (-10.98 to 0.83) Downgraded | No issues identified | Low |
| **Cheng 2016 - Overall complications** | HIGH | Per author-reported risk of bias assessments, no issues identified | I2 = 0%, and associated P value not reported; No action taken | No issues identified | RR = 0.48 (95% CI: 0.30–0.77); only 2 studies, sample size not reported Downgraded | No issues identified | Moderate |
| **Zhang 2018 - Intraoperative blood loss** | HIGH | Per author-reported risk of bias assessments, no issues identified | I2 = 95%, with a significant p value (p<0.00001) Downgraded | No issues identified | No issues identified | No issues identified | Moderate |
| **Zhang 2018 - Drainage volume** | HIGH | Per author-reported risk of bias assessments, no issues identified | I2 = 93%, with a significant p value (p<0.00001) Downgraded | No issues identified | mean difference = -134.13 (-216.55 to -51.71); large sample size, n=624 Downgraded given wide confidence interval | No issues identified | Low |
| **Zhang 2018 - Overall complications** | HIGH | Per author-reported risk of bias assessments, no issues identified | No issues identified | No issues identified | wide 95% CI: RR = 0.54 (0.28 to 1.03); only 2 studies included, n=297 Downgraded | No issues identified | Moderate |
| **Zhang 2018 - Operating time** | HIGH | Per author-reported risk of bias assessments, no issues identified | I2 = 81%, with a significant p value (p<0.00001) Downgraded | No issues identified | mean difference = -9.23 (-19.54 to 0.98); large sample size, n=678 Downgraded given that confidence interval is approaching threshold | No issues identified | Low |
| **Zhang 2018 - Length of stay** | HIGH | Per author-reported risk of bias assessments, no issues identified | I2 = 99%, with a significant p value (p<0.00001) Downgraded | No issues identified | mean difference= -1.35 (-2.37 to -0.34); large sample size, n=433 Downgraded given that confidence interval is approaching threshold | No issues identified | Low |
| **CHOLECYSTECTOMY** |  |  |  |  |  |  |  |
| **Jiang 2017 - Operating time** | HIGH | Assessed by authors | Assessed by authors | Assessed by authors | Assessed by authors | Assessed by authors | Low |
| **Jiang 2017 - Intraoperative blood loss** | HIGH | Assessed by authors | Assessed by authors | Assessed by authors | Assessed by authors | Assessed by authors | Low |
| **Jiang 2017 - Length of stay** | HIGH | Assessed by authors | Assessed by authors | Assessed by authors | Assessed by authors | Assessed by authors | Low |
| **Jiang 2017 - Pain** | HIGH | Assessed by authors | Assessed by authors | Assessed by authors | Assessed by authors | Assessed by authors | Moderate |
| **Sasi, 2010 - Operating time** | HIGH | Per author-reported risk of bias assessments, no issues identified | No issues identified | No issues identified | No issues identified | No issues identified | High |
| **Sasi, 2010 - Length of stay** | HIGH | Per author-reported risk of bias assessments, no issues identified | Information not reported; No action taken | No issues identified | weighted mean difference = -0.30 (-0.51 to -0.09); large sample size, n=469 Downgraded given that confidence interval is approaching threshold | No issues identified | Moderate |
| **Sasi, 2010 - Pain** | HIGH | Per author-reported risk of bias assessments, no issues identified | I2 = 97.6%, with a significant p value (p<0.00001) Downgraded | No issues identified | weighted mean difference = -0.94 (-1.06 to -0.82); only 2 studies included, n=273 Downgraded | No issues identified | Low |
| **Sasi, 2010 - Overall complications** | HIGH | Per author-reported risk of bias assessments, no issues identified | No issues identified | No issues identified | Wide 95% CI: OR = 0.45 (0.12 to 1.65); only 2 studies included, n=320 Downgraded | No issues identified | Moderate |
| **Xiong 2012 - Operating time** | HIGH | Per author-reported risk of bias assessments, no issues identified | I2 = 85%, with a significant p value (p=0.0002) Downgraded | No issues identified | No issues identified | No issues identified | Moderate |
| **Xiong 2012 - Intraoperative blood loss** | HIGH | Per author-reported risk of bias assessments, no issues identified | No issues identified | No issues identified | mean difference =-41.02 (-42.67 to -39.38); only 3 studies included, n=414 Downgraded | No issues identified | Moderate |
| **Xiong 2012 - Length of stay** | HIGH | Per author-reported risk of bias assessments, no issues identified | I2 = 93%, with a significant p value (p<0.00001) Downgraded | No issues identified | mean difference = -0.43 (-0.76 to -0.09); large sample size, n=703 Downgraded given that confidence interval is approaching threshold | No issues identified | Low |
| **Xiong 2012 - Pain** | HIGH | Per author-reported risk of bias assessments, no issues identified | No issues identified | No issues identified | No issues identified | No issues identified | High |
| **FLAP HARVESTING** |  |  |  |  |  |  |  |
| **Kim 2021 - Operating time** | HIGH | Per author-reported risk of bias assessments, no issues identified | I2 = 97%, with a significant p value (p<0.00001) Downgraded | No issues identified | No issues identified | No issues identified | Moderate |
| **Kim 2021 - Drainage volume** | HIGH | Per author-reported risk of bias assessments, no issues identified | I2 = 89%, with a significant p value (p<0.00001) Downgraded | No issues identified | Wide 95% CI: mean difference = -58.76 (-105.27 to -12.25); small sample size, n=124 Downgraded | No issues identified | Low |
| **Kim 2021 - Intraoperative blood loss** | HIGH | Per author-reported risk of bias assessments, no issues identified | I2 = 87%, not statistically significant (p=0.24) Downgraded | No issues identified | Wide 95% CI: mean difference = -44.68 (-119.19 to 29.82); only 3 studies included, n=98 Downgraded | No issues identified | Low |
| **NECK DISSECTION** |  |  |  |  |  |  |  |
| **Ren 2015 - Operating time** | HIGH | Per author-reported risk of bias assessments, no issues identified | I2 = 92%, with a significant p value (p<0.00001) Downgraded | No issues identified | No issues identified | No issues identified | Moderate |
| **Ren 2015 - Intraoperative blood loss** | HIGH | Per author-reported risk of bias assessments, no issues identified | I2 = 100%, with a significant p value (p<0.00001) Downgraded | No issues identified | Wide 95% CI: mean difference = -141.13 (-314.99 to 32.73); large sample size, n=304 Downgraded | No issues identified | Low |
| **Ren 2015 - Drainage volume** | HIGH | Per author-reported risk of bias assessments, no issues identified | I2 = 97%, with a significant p value (p<0.00001) Downgraded | No issues identified | mean difference = -64.86 (-110.40 to -19.32); large sample size, n=386 Downgraded given wideness of confidence interval | No issues identified | Low |
| **Ren 2015 - Length of stay** | HIGH | Per author-reported risk of bias assessments, no issues identified | No issues identified | No issues identified | Wide 95% CI: mean difference = -0.21 (-0.48 to 0.07); only 3 studies included, n=160 Downgraded | No issues identified | Moderate |
| **COLECTOMY** |  |  |  |  |  |  |  |
| **Tou 2011 - Intraoperative blood loss** | HIGH | Per author-reported risk of bias assessments, no issues identified | No issues identified | No issues identified | only 2 studies included in analyses, n~183 Downgraded | No issues identified | Moderate |
| **Tou 2011 - Overall complications** | HIGH | Per author-reported risk of bias assessments, no issues identified | No issues identified | No issues identified | Wide 95% CIs; only 3 studies included, n~200 Downgraded | No issues identified | Moderate |
| **Tou 2011 - Operating Time** | HIGH | Per author-reported risk of bias assessments, no issues identified | MES vs UCS: I2 = 87%, with a significant p value (p=0.01) Downgraded | No issues identified | only 2 studies included in analyses, n~183 Downgraded | No issues identified | Low |
| **Tou 2011 - Length of stay** | HIGH | Per author-reported risk of bias assessments, no issues identified | No issues identified | No issues identified | only 2 studies included in analyses, n~183 Downgraded | No issues identified | Moderate |

1 In the issues impacting GRADE score columns white cells indicate no issues identified for the topic whereas dark grey cells indicate an issue or issues for which the GRADE score was downgraded.

2 In the overall score column the cells are color coded in the following manner: red = very low, orange = low, yellow = moderate, and green = high.

Supplementary Table 6: NICE Checklist (2022) Assessment of Included RCTs

| Study | Was randomization carried out appropriately? | Was the concealment of treatment allocation adequate? | Were the groups similar at the outset of the study in terms of prognostic factors? | Were the care providers, participants, and the outcome assessors blind to treatment allocation? | Were the dropouts between groups balanced?^a^ | Is there a lack of evidence to suggest that the authors measured more outcomes than they reported?^a^ | Did the analysis include an intention-to-treat analysis? If so, was this appropriate and were appropriate methods used to account for missing data? | Did authors not have conflicts of interest?^a^ |
| --- | --- | --- | --- | --- | --- | --- | --- | --- |
| Bessa 2011 | U | Y | Y | N | Y | Y | Y | Y |
| Liao 2016 | Y | Y | Y | N | Y | Y | N | U |
| Mattila 2016 | Y | Y | Y | N | Y | Y | N | Y |
| Abdelhady 2017 | N | Y | Y | N | Y | Y | Y | Y |
| Sanawan 2017 | N | U | Y | N | Y | Y | Y | U |
| Shabbir 2017 | U | U | Y | N | Y | Y | Y | U |
| Ahmed 2019 | U | U | U | N | Y | Y | Y | Y |
| Awale 2019 | Y | Y | Y | N | Y | Y | Y | Y |
| Wilhelm 2011 | Y | Y | Y | N | Y | Y | Y | Y |
| Sista 2013 | Y | Y | Y | N | Y | Y | Y | U |
| Kawabata 2016 | U | U | Y | N | Y | Y | N | Y |
| Oh 2016 | U | U | Y | N | Y | Y | N | U |
| Kim 2018 | U | U | Y | N | Y | Y | N | Y |
| Tsunoda 2011 | Y | U | Y | N | Y | Y | Y | U |
| Peker 2013 | Y | Y | Y | N | Y | Y | Y | Y |
| Bilgin 2014 | U | U | Y | N | Y | Y | Y | Y |
| Bulus 2014 | U | U | U | N | Y | Y | Y | Y |
| Shoukat 2016 | U | U | U | N | U | Y | U | U |
| Ahmed 2021 | N | U | N | N | Y | Y | Y | Y |
| Nawaz 2015 | Y | U | U | N | Y | Y | Y | U |
| Mittal 2017 | U | U | Y | N | Y | Y | Y | Y |
| Shanmugam 2017 | U | U | Y | N | Y | Y | Y | Y |
| Archana 2018 | U | U | Y | U | Y | Y | Y | Y |
| Faisal 2018 | Y | Y | Y | N | Y | Y | Y | Y |
| Sarwar 2016 | N | U | U | N | Y | Y | Y | U |
| Salama 2020 | Y | Y | Y | N | Y | Y | Y | Y |
| Deori 2021 | U | Y | Y | N | U | Y | U | Y |
| Mathialagan 2016 | Y | U | Y | N | Y | Y | Y | Y |
| Verma 2017 | Y | U | Y | N | Y | Y | Y | Y |
| Schneider 2018 | Y | U | U | N | Y | Y | Y | Y |
| Vaira 2021 | U | U | Y | N | Y | Y | Y | Y |
| He 2011 | U | U | Y | N | Y | Y | Y | Y |
| Mourad 2011 | Y | Y | Y | N | Y | Y | Y | Y |
| Yener 2014 | U | U | Y | N | Y | Y | Y | U |
| Aziz 2016 | U | U | U | N | Y | Y | Y | Y |
| Docimo 2016 | U | U | Y | N | Y | Y | Y | Y |
| Su 2016 | Y | U | Y | N | Y | Y | Y | U |
| Anandaravi 2017 | U | U | U | N | Y | Y | Y | Y |
| Basurto-Kuba 2017 | U | U | Y | N | Y | Y | Y | Y |
| Shaaban 2017 | Y | Y | Y | N | Y | Y | Y | Y |
| Uludag 2017 | N | Y | Y | N | Y | Y | Y | Y |
| Ahmad 2018 | U | U | N | N | Y | Y | Y | U |
| Jamil 2019a | U | U | N | N | Y | Y | Y | Y |
| Jamil 2019b | U | U | N | N | Y | Y | Y | Y |
| Kadem 2019 | N | U | Y | N | Y | Y | Y | Y |
| Ansari 2020 | N | U | U | N | Y | Y | Y | U |
| Buzdar 2020 | U | U | U | N | Y | Y | Y | Y |
| Ali 2011 | Y | U | U | N | Y | Y | Y | Y |
| Pajic-Penavic 2013 | U | U | U | U | Y | Y | Y | Y |
| Arbin 2017 | U | Y | Y | N | U | Y | N | Y |
| Karimi 2017 | Y | U | U | N | Y | Y | Y | U |
| Basu 2019 | Y | U | U | N | Y | Y | N | Y |
| Sah 2019 | N | U | U | N | Y | Y | Y | U |
| Kwek 2020 | Y | Y | Y | N | Y | Y | Y | Y |
| Subasi 2021 | U | Y | U | N | Y | Y | Y | Y |
| Hwang 2014 | N | U | Y | N | Y | Y | Y | Y |
| Ciftci 2016 | U | Y | Y | N | Y | Y | Y | Y |
| Back 2019 | Y | Y | N | N | Y | Y | Y | Y |
| Papavramidis 2020 | N | Y | Y | N | Y | Y | Y | Y |
| Kim 2021 | Y | U | N | N | Y | Y | Y | Y |
| ***Orphan RCTs*** |  |  |  |  |  |  |  |  |
| Duscher 2019 | N | N | U | N | Y | Y | N | Y |
| Qaiser 2021 | Y | Y | U | N | N | Y | N | Y |
| Tremp 2012 | U | U | Y | N | Y | Y | Y | U |
| Burdette 2011 | U | Y | Y | N | Y | Y | Y | U |
| Hanyong 2015 | N | Y | Y | N | Y | Y | Y | Y |
| Sultan 2019 | U | Y | Y | N | Y | Y | Y | U |
| El Shobary 2017 | N | N | Y | N | Y | Y | Y | U |
| Olmez 2012 | N | N | Y | N | Y | Y | Y | U |
| Fitz-Gerald 2013 | Y | Y | Y | N | Y | Y | Y | U |
| Choi 2018 | Y | Y | Y | N | Y | Y | Y | Y |
| Rothmund 2013 | Y | U | U | N | Y | Y | Y | Y |
| Litta 2010 | Y | Y | Y | N | Y | Y | N | Y |
| Uzunoglu 2012 | Y | U | Y | N | Y | Y | N | Y |
| Landoni 2021 | Y | Y | U | N | Y | Y | N | Y |
| He 2014 | U | U | Y | N | Y | Y | Y | U |
| Deganello 2014 | U | U | Y | N | Y | Y | Y | U |
| Uysal 2019 | U | U | Y | N | Y | Y | Y | Y |
| Fritz 2016 | Y | Y | Y | N | Y | Y | N | Y |
| Pogorelic 2017 | N | U | Y | N | Y | Y | Y | Y |
| Roy 2018 | Y | Y | Y | N | Y | Y | Y | Y |
| Tsamis 2015 | Y | Y | Y | N | Y | Y | Y | Y |
| Pastore 2013 | U | U | Y | N | Y | Y | Y | Y |
| Toishi 2014 | N | U | Y | N | Y | Y | N | Y |

^a^ These questions were inverted so that Y was a positive response and N was negative response.

The cells for the NICE checklist assessments are color coded based on whether the study appropriately addressed the potential for bias in the following manner: Red “N” = potential bias was not addressed, yellow “U” = unclear whether potential bias was addressed, and green “Y” = potential bias was addressed.

Supplementary Table 7: Surgical outcomes from most comprehensive SLRs reporting on Harmonic versus conventional

| **Specialty** | **Sample Size** | **Effect (95% CI)** |
| --- | --- | --- |
| **Operating time** | | |
| Cholecystectomy (Jiang, 2017) | n = 1 323 | -14.86 (-21.45 to -8.27)* |
| Colectomy (Tou, 2011) | n = 186 | -26.2 (-62 to 9.6) |
| Flap Harvesting (Kim, 2022) | n=178 | -26.29 (-39.38 to -13.2)* |
| Gastrectomy (Cheng, 2015) | n = 781 | -27.5 (-42.2 to -12.81)* |
| Hemorrhoidectomy (Mushaya 2014) | n = 307 | -1.84 (-3.6 to -0.13)* |
| Mastectomy (Zhang, 2018) | n = 678 | -9.23 (-19.43 to 0.98) |
| Neck dissection (Ren, 2015) | n = 406 | -29.29 (-44.26 to -14.32)* |
| Thyroidectomy (Aires, 2018) | n = 5 298 | -25.76 (-31.6 to -19.91)* |
| Tonsillectomy (Alexiou, 2011) | n = 535 | -0.1 (-6.26 to 6.05) |
| **Overall complications** | | |
| Cholecystectomy (Sasi, 2010) | n = 320 | 0.45 (0.12 to 1.65) |
| Colectomy (Tou, 2011) | n = 209 | 0.78 (0.44 to 1.39) |
| Gastrectomy (Cheng, 2015) | n = 464 | 0.58 (0.33 to 1.02) |
| Hemorrhoidectomy (Mushaya 2014) | n = 468 | 0.45 (0.28 to 0.72)* |
| Mastectomy (Zhang, 2018) | n = 297 | 0.54 (0.28 to 1.03) |
| Thyroidectomy (Cannizzaro, 2016) | n = 1920 | 0.82 (0.47 to 1.41) |
| **Hospital Stay (days)** | | |
| Cholecystectomy (Jiang, 2017) | n = 992 | -0.37 (-0.61 to -0.14)* |
| Colectomy (Tou, 2011) | n = 186 | -0.42 (-0.84 to 0) |
| Gastrectomy (Cheng, 2015) | n = 162 | -0.63 (-2.48 to 1.23) |
| Hemorrhoidectomy (Mushaya 2014) | n = 190 | -0.6 (-1.3 to 0.15) |
| Mastectomy (Zhang, 2018) | n = 433 | -1.35 (-2.73 to -0.34)* |
| Neck dissection (Ren, 2015) | n = 160 | -0.21 (-0.48 to 0.07) |
| Thyroidectomy (Cannizzaro, 2016) | n = 1 535 | -0.536 (-0.797 to -0.275)* |
| **Blood loss** | | |
| Cholecystectomy (Jiang, 2017) | n = 594 | -47.24 (-79.57 to -14.9)* |
| Colectomy (Tou, 2011) | n = 186 | -42.09 (-62.02 to -22.16)* |
| Flap Harvesting (Kim, 2022) | n = 98 | -44.68 (-119.19 to 29.82) |
| Gastrectomy (Cheng, 2015) | n = 685 | -93.15 (-125.29 to -61)* |
| Mastectomy (Cheng, 2016) | n = 644 | -87.54 (-137.07 to -38.02)* |
| Neck dissection (Ren, 2015) | n = 304 | -141.13 (-314.99 to 32.73) |
| Thyroidectomy (Aires, 2018) | n = 699 | -36.97 (-39.69 to -34.26)* |
| Tonsillectomy (Alexiou, 2011) | n = 535 | -37.71 (-52.98 to -22.43)* |
| **Pain** | | |
| Cholecystectomy (Jiang, 2017) | n = 725 | -0.95 (-1.4 to -0.5)* |
| Hemorrhoidectomy (Balciscueta, 2021) | n = 278 | -1.63 (-3.11 to -0.15)* |
| Thyroidectomy (Cannizzaro, 2016) | n = 252 | -1.88 (-2.35 to -1.41)* |
| Tonsillectomy (Alexiou, 2011) | n = 517 | -0.38 (-1.2 to 0.43) |
| **Drainage Volume** | | |
| Flap Harvesting (Kim, 2022) | n = 124 | -58.76 (-105.27 to -12.25)* |
| Gastrectomy (Cheng, 2015) | n = 734 | -138.83 (-177.57 to -100.1)* |
| Mastectomy (Zhang, 2018) | n = 624 | -134.13 (-216.55 to -51.71)* |
| Neck dissection (Ren, 2015) | n = 386 | -64.86 (-110.4 to -19.32)* |
| Thyroidectomy (Aires, 2018) | n = 292 | -29.38 (-52.46 to -6.3)* |

Asterisks indicate statistically significant differences between Harmonic and comparators.

References

1. Bessa SS, Abdel-Razek AH, Sharaan MA, Bassiouni AE, El-Khishen MA, El-Kayal el SA. Laparoscopic cholecystectomy in cirrhotics: a prospective randomized study comparing the conventional diathermy and the harmonic scalpel for gallbladder dissection. J Laparoendosc Adv Surg Tech A. 2011;21(1):1-5.

2. Liao G, Wen S, Xie X, Wu Q. Harmonic scalpel versus monopolar electrocauterization in cholecystectomy. JSLS: Journal of the Society of Laparoendoscopic Surgeons. 2016;20(3):e2016.00037.

3. Mattila A, Mrena J, Kautiainen H, Nevantaus J, Kellokumpu I. Day-care laparoscopic cholecystectomy with diathermy hook versus fundus-first ultrasonic dissection: a randomized study. Surgical Endoscopy. 2016;30(9):3867-72.

4. Abdelhady MH, Salama AF. Clipped vs clipless laparoscopic cholecystectomy using the ultrasonically activated (harmonic) scalpel. World Journal of Laparoscopic Surgery. 2017;10(1):17-21.

5. Sanawan E, Qureshi AU, Qureshi SS, Cheema KM, Cheema MA. Effectiveness of Ultrasound Shear for Clipless Laparoscopic Cholecystectomy Versus Conventional Unipolar Electrocautery in Patients with Cholelithiasis. J Coll Physicians Surg Pak. 2017;27(10):611-5.

6. Shabbir A, Hussain S. Comparison of Gallbladder Perforation During Dissection from Liver Bed in Patients Undergoing Monopolar Electrocautery with Those Undergoing Ultrasonic Dissection during Lap. Cholecystectomy. Pak J Med Health Sci. 2016;10(4):1390-2.

7. Ahmed A, Jamil M, Anwer Q. Changing trends in laparoscopic cholecystectomy: Clip less versus conventional technique. Rawal Medical Journal. 2019;44(2):314-.

8. Awale L, Pandit N, Adhikary S. Clipless laparoscopic cholecystectomy: ultrasonic dissection vs conventional method. World Journal of Laparoscopic Surgery. 2019;12(3):121-5.

9. Wilhelm D, Szabo M, Glass F, Schuhmacher C, Friess H, Feussner H. Randomized controlled trial of ultrasonic dissection versus standard surgical technique in open left hemicolectomy or total gastrectomy. Journal of British Surgery. 2011;98(2):220-7.

10. Sista F, Abruzzese V, Schietroma M, Cecilia EM, Mattei A, Amicucci G. New harmonic scalpel versus conventional hemostasis in right colon surgery: a prospective randomized controlled clinical trial. Dig Surg. 2013;30(4-6):355-61.

11. Kawabata R, Takiguchi S, Kimura Y, Imamura H, Fujita J, Tamura S, et al. A randomized phase II study of the clinical effects of ultrasonically activated coagulating shears (Harmonic scalpel) in open gastrectomy for gastric cancer. Surgery today. 2016;46(5):561-8.

12. Oh SY, Choi B, Lee KG, Choe HN, Lee HJ, Suh YS, et al. Ultrasonically Activated Shears Reduce Blood Loss without Increasing Inflammatory Reactions in Open Distal Gastrectomy for Cancer: A Randomized Controlled Study. Ann Surg Oncol. 2017;24(2):494-501.

13. Kim SM, Bae J-M, Choi M-G, Lee JH, Sohn TS, Kim S. Oncological safety of use of ultrasonic activated shears in gastric cancer surgery: Long-term results of randomized controlled trial. Chinese Journal of Cancer Research. 2018;30(5):492.

14. Tsunoda A, Sada H, Sugimoto T, Kano N, Kawana M, Sasaki T, et al. Randomized controlled trial of bipolar diathermy vs ultrasonic scalpel for closed hemorrhoidectomy. World J Gastrointest Surg. 2011;3(10):147-52.

15. Peker K, Inal A, Gullu H, Gul D, Sahin M, Ozcan AD, et al. Comparison of vessel sealing systems with conventional. Iran Red Crescent Med J. 2013;15(6):488-96.

16. Bilgin Y, Hot S, Barlas IS, Akan A, Eryavuz Y. Short- and long-term results of harmonic scalpel hemorrhoidectomy versus stapler hemorrhoidopexy in treatment of hemorrhoidal disease. Asian J Surg. 2015;38(4):214-9.

17. Bulus H, Tas A, Coskun A, Kucukazman M. Evaluation of two hemorrhoidectomy techniques: harmonic scalpel and Ferguson's with electrocautery. Asian J Surg. 2014;37(1):20-3.

18. Shoukat HI, M; Ullah, S; Mirza, A; Dar, UF; Dar, UF. Comparison of Hemorrhoidectomy Using Bipolar Diathermy Vs Harmonic Scalpel. Pakistan Journal of Medical & Health Sciences. 2016;10(2):489-91.

19. Ahmad M, Abbas ST, Javaid A, Arshad N, Shair F. Comparison of harmonic scalpel versus Milligan Morgan technique in haemorrhoidectomy patients. Journal of the Pakistan Medical Association. 2021;71(10):2369-72.

20. Nawaz A, Waqar S, Khan A, Mansoor R, Butt UI, Ayyaz M. Harmonic Scalpel Versus Electrocautery in Axillary Dissection in Carcinoma Breast. J Coll Physicians Surg Pak. 2015;25(12):870-3.

21. Mittal P, Kumar A, Kaur S, Pandove PK, Singla RL, Singh J. A Comparative Study of the Use of Harmonic Scalpel versus Unipolar Cautery in Modified Radical Mastectomy. Niger J Surg. 2017;23(1):20-5.

22. Shanmugam S, Govindasamy G, Hussain SA, Rao PSH. Axillary dissection for breast cancer using electrocautery versus ultrasonic dissectors: A prospective randomized study. Indian J Cancer. 2017;54(3):543-6.

23. Archana A, Sureshkumar S, Vijayakumar C, Palanivel C. Comparing the Harmonic Scalpel with Electrocautery in Reducing Postoperative Flap Necrosis and Seroma Formation after Modified Radical Mastectomy in Carcinoma Breast Patients: A Double-Blind Prospective Randomized Control Trail. Cureus. 2018;10(4):e2476.

24. Faisal M, Fathy H, Shaban H, Abuelela ST, Marie A, Khaled I. A novel technique of harmonic tissue dissection reduces seroma formation after modified radical mastectomy compared to conventional electrocautery: a single-blind randomized controlled trial. Patient Saf Surg. 2018;12:8.

25. Sarwar G, Sheikh TH, Nadeem M. Comparison of blood loss between harmonic scalpel and monopolar electrocautery in modified radical mastectomy. PJMHS. 2016;10:649-51.

26. Salama AMF, Nawar AM, Zayed ME, Essa MS. Evaluation of ultrasonic axillary dissection in preservation of intercostobrachial nerve and lymphatic sealing in breast cancer patients: Randomized controlled trial. Ann Med Surg (Lond). 2020;60:255-60.

27. Deori A, Gupta N, Gupta AK, Yelamanchi R, Agrawal H, Durga CK. A Prospective Randomised Controlled Study Comparing Ultrasonic Dissector with Electrocautery for Axillary Dissection in Patients of Carcinoma Breast. Malays J Med Sci. 2021;28(1):97-104.

28. Mathialagan A, Verma RK, Panda NK. Comparison of spinal accessory dysfunction following neck dissection with harmonic scalpel and electrocautery–a randomized study. Oral Oncology. 2016;61:142-5.

29. Verma RK, Mathiazhagan A, Panda NK. Neck dissection with harmonic scalpel and electrocautery? A randomised study. Auris Nasus Larynx. 2017;44(5):590-5.

30. Schneider D, Goppold K, Kaemmerer PW, Schoen G, Woehlke M, Bschorer R. Use of ultrasonic scalpel and monopolar electrocautery for skin incisions in neck dissection: a prospective randomized trial. Oral Maxillofac Surg. 2018;22(2):169-75.

31. Vaira LA, De Riu G, Ligas E, Deiana G, Vacca G, Massarelli O, et al. Neck dissection with harmonic instruments and electrocautery: a prospective comparative study. Oral Maxillofac Surg. 2021;25(1):75-9.

32. He Q, Zhuang D, Zheng L, Zhou P, Chai J, Lv Z. Harmonic focus in total thyroidectomy plus level III-IV and VI dissection: a prospective randomized study. World J Surg Oncol. 2011;9:141.

33. Mourad M, Rulli F, Robert A, Scholtes JL, De Meyer M, De Pauw L. Randomized clinical trial on Harmonic Focus shears versus clamp-and-tie technique for total thyroidectomy. Am J Surg. 2011;202(2):168-74.

34. Yener O, Demir M, Yilmaz A, Yigitbasi R, Atak T. Harmonic scalpel compared to conventional hemostasis in thyroid surgery. Indian J Surg. 2014;76(1):66-9.

35. Aziz W, Khan MS, Assad S, Siddique G. Suture-less Thyroidectomy Using Harmonic Scalpel versus Conventional Thyroidectomy: A Randomized Controlled Trial. Journal of Pioneering Medical Sciences. 2016;6(2).

36. Docimo G, Tolone S, Conzo G, Limongelli P, Del Genio G, Parmeggiani D, et al. A gelatin–thrombin matrix topical hemostatic agent (Floseal) in combination with Harmonic scalpel is effective in patients undergoing total thyroidectomy: a prospective, multicenter, single-blind, randomized controlled trial. Surgical innovation. 2016;23(1):23-9.

37. Su L, Li J, Tang X, Sang J. Therapeutic Effects of Bipolar Coagulation Forceps on Open Thyroid Surgery. Rev Invest Clin. 2016;68(5):256-61.

38. Anandaravi B, Aslam MA, Nair PP. Prospective randomised study using focus harmonic scalpel versus conventional hemostasis for vessel ligation in open thyroid surgery. International Surgery Journal. 2017;4(4):1431-7.

39. Basurto-Kuba EOP, Robles-Estrada M, Hurtado-Lopez LM, EDM EO-D, Campos-Castillo C, Zaldivar-Ramirez FR, et al. Safety and Cost-Effectiveness in Thyroidectomy Using the HARMONIC Scalpel Compared to Traditional Hemostasis: A Controlled Clinical Assay. Surg Technol Int. 2017;30:141-7.

40. Shaaban A, Ramadan R. Sutureless thyroidectomy for controlled toxic goiter: a single-institute experience. The Egyptian Journal of Surgery. 2017;36(1):62-8.

41. Uludag SS, Teksoz S, Arikan AE, Tarhan O, Yener HM, Ozcan M, et al. Effect of energy-based devices on voice quality after total thyroidectomy. Eur Arch Otorhinolaryngol. 2017;274(5):2295-302.

42. Ahmad D, Imran K, Shakil S. Harmonic Scalpel Compared to Conventional Homeostasis in Thyroid Surgery. PAKISTAN JOURNAL OF MEDICAL & HEALTH SCIENCES. 2018;12(1):46-9.

43. Jamil A, Javed R, Ashraf S, Amin I, Shakoor S, Laique T. Comparison of Harmonic Scalpel Method With Conventional Procedure for Hospital Stay During Thyroidectomy among Pakistani Patients. Pakistan Journal of Medical Sciences. 2019;13(4):1296-8.

44. Jamil A, Javed R, Nadeem N, Shujaat K, Shakoor S, Babar A, et al. Comparison Of Harmonic Scalpel Method With Conventional Procedure For Intraoperative Blood Loss During Thyroidectomy Among Pakistani Patients. Pakistan Journal of Medical Sciences. 2019;13(4):1172-4.

45. Kadem SG, Alabbood MH. Safety and Efficacy of Bipolar Radiofrequency Ablation Device in Hemostasis during Thyroidectomy in Comparison with Ultrasonic Scalpel: A Comparative Study. Indian J Endocrinol Metab. 2019;23(1):76-80.

46. Ansari MSH, Iqbal H, Anjum Z. Outcomes of Harmonics Use in Thyroid Surgery: A Randomized Control Trial. Pakistan Journal of Medical Sciences. 2020;14(3):522-4.

47. Fida Buzdar MAK, Majeed Ullah Buzdar, Imran Asim, Hafeez Ullah Laghari and Rizwan Ahmad. Role of Harmonic Scalpel Versus Conventional Haemostasis Among Patients Undergoing Total Thyroidectomy. Medical Forum Monthly. 2020;31(5):7-9.

48. Ali NS, Ikram M, Akhtar S, Moghira I, Nawaz A, Arain A. Harmonic scalpel versus electrocautery tonsillectomy: a comparative study in adult patients. J Pak Med Assoc. 2011;61(3):256-9.

49. Pajić-Penavić I, Đanić D, Mrzljak-Vučinić N, Matić I, Vuković-Arar Ž, Dikanović M. Postoperative quality of life after two different methods of tonsillectomy. Wiener klinische Wochenschrift. 2013;125(17):524-8.

50. Arbin L, Enlund M, Knutsson J. Post-tonsillectomy pain after using bipolar diathermy scissors or the harmonic scalpel: a randomised blinded study. European Archives of Oto-Rhino-Laryngology. 2017;274(5):2281-5.

51. Karimi E, Safaee A, Bastaninejad S, Dabiran S, Masoumi E, Moravej Salehi F. A Comparison between Cold Dissection Tonsillectomy and Harmonic Scalpel Tonsillectomy. Iran J Otorhinolaryngol. 2017;29(95):313-7.

52. Basu S, Sengupta A, Dubey AB, Sengupta A. Harmonic Scalpel Versus Coblation Tonsillectomy A Comparative Study. Indian J Otolaryngol Head Neck Surg. 2019;71(4):498-503.

53. Sah MK, Neupane Y, Guragain RP. Comparison of Ultrasonic Device Versus Bipolar Diathermy Tonsillectomy in Children. J Nepal Health Res Counc. 2019;17(1):71-5.

54. Kwek WMJ, Chua S, Xu SH, Tan THL, Huang XY, Loh I, et al. Randomized controlled study comparing tonsillectomy safety and patient satisfaction outcomes between HARMONIC ACE(R) + shears and monopolar diathermy in an adult population - A pilot study. Am J Otolaryngol. 2020;41(5):102568.

55. Subasi B, Oghan F, Tasli H, Akbal S, Karaman NE. Comparison of three tonsillectomy techniques in children. Eur Arch Otorhinolaryngol. 2021;278(6):2011-5.

56. Hwang SO, Jung JH, Park HY, Kim WW. A Prospective, Randomized Study between the Small Jaw(R) and the Harmonic Focus(R) in Open Thyroidectomy. Otolaryngol Head Neck Surg. 2014;150(6):943-8.

57. Ciftci F, Sakalli E, Abdurrahman I, Guler B. Parathyroid function following total thyroidectomy using energy devices. Eur Arch Otorhinolaryngol. 2016;273(7):1905-11.

58. Back K, Hur N, Kim MJ, Choe J-H, Kim J-H, Kim JS. A prospective, randomized, controlled comparative study of three energy devices in open thyroid surgery: thunderbeat, harmonic, and ligasure. Journal of Endocrine Surgery. 2019;19(4):106-15.

59. Papavramidis TS, Pliakos I, Chorti A, Panidis S, Kotsovolis G, Stelmach V, et al. Comparing LigasureTM Exact dissector with other energy devices in total thyroidectomy: a pilot study. Gland Surgery. 2020;9(2):271.

60. Kim WW, Cho J, Jeon YS, Kim I, Jeong YJ, Choi J, et al. Prospective, randomized, comparative, multicenter study of the hybrid ultrasonic advanced bipolar device and the ultrasonic coagulating shears in open thyroidectomy. Surgical Innovation. 2021;28(1):41-7.

61. Duscher D, Aitzetmuller MM, Shan JJ, Wenny R, Brett EA, Staud CJ, et al. Comparison of Energy-Based Tissue Dissection Techniques in Abdominoplasty: A Randomized, Open-Label Study Including Economic Aspects. Aesthet Surg J. 2019;39(5):536-43.

62. Qaiser MU, Nazir A, Khan MS, Butt HK, Anwar M. Comparison of Ultrasonic Dissection and Suture Ligation for Mesoappendix in Laparoscopic Appendectomy. Cureus. 2021;13(4):e14316.

63. Tremp M, Di Summa PG, Schaakxs D, Rieger U, Raffoul W, Schaefer DJ, et al. Is ultracision knife safe and efficient for breast capsulectomy? A preliminary study. Aesthetic plastic surgery. 2012;36(4):888-93.

64. Burdette TE, Kerrigan CL, Homa KA. Harmonic scalpel versus electrocautery in breast reduction surgery: a randomized controlled trial. Plast Reconstr Surg. 2011;128(4):243e-9e.

65. Hanyong S, Wanyee L, Siyuan F, Hui L, Yuan Y, Chuan L, et al. A prospective randomized controlled trial: comparison of two different methods of hepatectomy. Eur J Surg Oncol. 2015;41(2):243-8.

66. Sultan AM, Shehta A, Salah T, Elshoubary M, Elghawalby AN, Said R, et al. Clamp-Crush Technique Versus Harmonic Scalpel for Hepatic Parenchymal Transection in Living Donor Hepatectomy: a Randomized Controlled Trial. J Gastrointest Surg. 2019;23(8):1568-77.

67. El Shobary M, El Nakeeb A, Sultan AM, Elghawalby A, Fathy O, Wahab MA, et al. Spray diathermy versus harmonic scalpel technique for hepatic parenchymal transection of living donor. Journal of Gastrointestinal Surgery. 2017;21(2):321-9.

68. Olmez A, Karabulut K, Aydin C, Kayaalp C, Yilmaz S. Comparison of harmonic scalpel versus conventional knot tying for transection of short hepatic veins at liver transplantation: prospective randomized study. Transplant Proc. 2012;44(6):1717-9.

69. Fitz-Gerald AL, Tan J, Chan KW, Polyakov A, Edwards GN, Najjar H, et al. Comparison of ultrasonic shears and traditional suture ligature for vaginal hysterectomy: randomized controlled trial. J Minim Invasive Gynecol. 2013;20(6):853-7.

70. Choi C, Do IG, Song T. Ultrasonic versus monopolar energy-based surgical devices in terms of surgical smoke and lateral thermal damage (ULMOST): a randomized controlled trial. Surg Endosc. 2018;32(11):4415-21.

71. Rothmund R, Szyrach M, Reda A, Enderle MD, Neugebauer A, Taran FA, et al. A prospective, randomized clinical comparison between UltraCision and the novel sealing and cutting device BiCision in patients with laparoscopic supracervical hysterectomy. Surg Endosc. 2013;27(10):3852-9.

72. Litta P, Fantinato S, Calonaci F, Cosmi E, Filippeschi M, Zerbetto I, et al. A randomized controlled study comparing harmonic versus electrosurgery in laparoscopic myomectomy. Fertil Steril. 2010;94(5):1882-6.

73. Uzunoglu FG, Stehr A, Fink JA, Vettorazzi E, Koenig A, Gawad KA, et al. Ultrasonic dissection versus conventional dissection techniques in pancreatic surgery: a randomized multicentre study. Annals of surgery. 2012;256(5):675-80.

74. Landoni L, De Pastena M, Fontana M, Malleo G, Esposito A, Casetti L, et al. A randomized controlled trial of stapled versus ultrasonic transection in distal pancreatectomy. Surgical Endoscopy. 2022;36(6):4033-41.

75. He Q, Zhuang D, Zheng L, Fan Z, Zhu J, Zhou P, et al. Harmonic focus compared with classic hemostasis during total parathyroidectomy in secondary hyperparathyroidism: a prospective randomized trial. Am Surg. 2014;80(12):E342-5.

76. Deganello A, Meccariello G, Busoni M, Parrinello G, Bertolai R, Gallo O. Dissection with harmonic scalpel versus cold instruments in parotid surgery. B-ent. 2014;10(3):175-8.

77. Uysal D, Gulmen S, Ozkan H, Saglam U, Etli M, Bircan S, et al. Comparison of Sharp Dissection, Electrocautery, and Ultrasonic Activated Scalpel with Regard to Endothelial Damage, Preparation Time, and Postoperative Bleeding During Radial Artery Harvesting. Braz J Cardiovasc Surg. 2019;34(6):667-73.

78. Fritz DK, Matthews TW, Chandarana SP, Nakoneshny SC, Dort JC. Harmonic scalpel impact on blood loss and operating time in major head and neck surgery: a randomized clinical trial. J Otolaryngol Head Neck Surg. 2016;45(1):58.

79. Pogorelic Z, Katic J, Mrklic I, Jeroncic A, Susnjar T, Jukic M, et al. Lateral thermal damage of mesoappendix and appendiceal base during laparoscopic appendectomy in children: comparison of the harmonic scalpel (Ultracision), bipolar coagulation (LigaSure), and thermal fusion technology (MiSeal). J Surg Res. 2017;212:101-7.

80. Roy KK, Gc N, Singhal S, Bharti J, Kumar S, Mitra DK, et al. Impact of energy devices on the post-operative systemic immune response in women undergoing total laparoscopic hysterectomy for benign disease of the uterus. J Turk Ger Gynecol Assoc. 2018;19(1):1-6.

81. Tsamis D, Natoudi M, Arapaki A, Flessas I, Papailiou I, Bramis K, et al. Using Ligasure™ or Harmonic Ace® in laparoscopic sleeve gastrectomies? A prospective randomized study. Obesity surgery. 2015;25(8):1454-7.

82. Pastore AL, Palleschi G, Silvestri L, Leto A, Sacchi K, Pacini L, et al. Prospective randomized study of radiofrequency versus ultrasound scalpels on functional outcomes of laparoscopic radical prostatectomy. Journal of Endourology. 2013;27(8):989-93.

83. Toishi M, Yoshida K, Agatsuma H, Sakaizawa T, Eguchi T, Saito G, et al. Usefulness of vessel-sealing devices for≤ 7 mm diameter vessels: a randomized controlled trial for human thoracoscopic lobectomy in primary lung cancer. Interactive cardiovascular and thoracic surgery. 2014;19(3):448-55.
